# Supplementary material for: Association between the composite dietary antioxidant index and constipation: Evidence from NHANES 2005–2010
Source: PLoS One. 2024 Sep 27;19(9):e0311168. doi: 10.1371/journal.pone.0311168 (PMC11432863; doi:10.1371/journal.pone.0311168)
Supplement: S1 File — (ZIP) [file pone.0311168.s001.zip › CDAI/all/PROJ2_10_tbl/PROJ2_10_tbl.htm]

|  |
| --- |
| BIANMI24 vs. CDAI23 |

Generalize additive models
Outcome: BIANMI24
Exposure: CDAI23
Linear terms effect

|  |  |  |  |  |  |  |  |
| --- | --- | --- | --- | --- | --- | --- | --- |
|  | Estimate | Std. Error | z value | Pr(>|z|) | exp(est) | 95%CI low | 95%CI upp |
| (Intercept) | -1.1634 | 0.6374 | -1.8254 | 0.0679 | 0.3124 | 0.0896 | 1.0896 |
| factor(DRINK10)2 | 0.1784 | 0.0762 | 2.3408 | 0.0192 | 1.1953 | 1.0294 | 1.3879 |
| factor(ZHONGZU3)2 | 0.3053 | 0.1314 | 2.3229 | 0.0202 | 1.357 | 1.0488 | 1.7556 |
| factor(ZHONGZU3)3 | 0.2241 | 0.105 | 2.1351 | 0.0328 | 1.2512 | 1.0185 | 1.5369 |
| factor(ZHONGZU3)4 | 0.5573 | 0.1127 | 4.9451 | 0 | 1.7459 | 1.3999 | 2.1774 |
| factor(ZHONGZU3)5 | 0.1001 | 0.1939 | 0.5161 | 0.6058 | 1.1052 | 0.7558 | 1.6162 |
| factor(XIYAN11)2 | -0.1588 | 0.1058 | -1.5007 | 0.1334 | 0.8531 | 0.6933 | 1.0498 |
| factor(XIYAN11)3 | 0.0887 | 0.0866 | 1.0241 | 0.3058 | 1.0927 | 0.9222 | 1.2948 |
| GAOXUEYA12 | 0.1836 | 0.0765 | 2.3995 | 0.0164 | 1.2016 | 1.0342 | 1.3961 |
| TANGNIAOBING13 | -0.009 | 0.1013 | -0.0889 | 0.9291 | 0.991 | 0.8126 | 1.2086 |
| FEIBING14 | -0.1038 | 0.0861 | -1.2059 | 0.2278 | 0.9014 | 0.7614 | 1.0671 |
| XINGZHANGBING15 | -0.3313 | 0.1191 | -2.782 | 0.0054 | 0.718 | 0.5685 | 0.9067 |
| GANBING16 | 0.231 | 0.1946 | 1.1872 | 0.2352 | 1.2598 | 0.8604 | 1.8447 |
| DANBAIZHI17 | 0.0044 | 0.0026 | 1.6928 | 0.0905 | 1.0044 | 0.9993 | 1.0096 |
| TANSHUI18 | 0.0062 | 0.0015 | 4.1223 | 0 | 1.0062 | 1.0033 | 1.0092 |
| XIANWEI19 | -0.0213 | 0.0065 | -3.2595 | 0.0011 | 0.979 | 0.9665 | 0.9916 |
| ZHIFANG20 | 0.0057 | 0.0037 | 1.556 | 0.1197 | 1.0057 | 0.9985 | 1.013 |
| SHUIFEN21 | -1e-04 | 0 | -3.3499 | 8e-04 | 0.9999 | 0.9998 | 0.9999 |
| NENGLIANG22 | -0.001 | 4e-04 | -2.6388 | 0.0083 | 0.999 | 0.9983 | 0.9998 |
| XINBIE1 | 0.8851 | 0.0803 | 11.0289 | 0 | 2.4233 | 2.0706 | 2.8361 |
| AGE2 | -0.0064 | 0.0026 | -2.4761 | 0.0133 | 0.9936 | 0.9886 | 0.9987 |
| factor(JIAOYU4)2 | -0.0581 | 0.0881 | -0.6587 | 0.5101 | 0.9436 | 0.7939 | 1.1215 |
| factor(JIAOYU4)3 | -0.4053 | 0.0858 | -4.725 | 0 | 0.6668 | 0.5636 | 0.7889 |
| factor(HUNYING5)2 | 0.0542 | 0.0823 | 0.6585 | 0.5102 | 1.0557 | 0.8984 | 1.2406 |
| factor(HUNYING5)3 | 0.0238 | 0.0934 | 0.2549 | 0.7988 | 1.0241 | 0.8528 | 1.2297 |
| PIR6 | -0.1363 | 0.0694 | -1.9635 | 0.0496 | 0.8726 | 0.7616 | 0.9998 |
| factor(BMI7)2 | -0.1792 | 0.0799 | -2.242 | 0.025 | 0.8359 | 0.7147 | 0.9777 |
| factor(BMI7)3 | -0.4259 | 0.0828 | -5.1416 | 0 | 0.6532 | 0.5553 | 0.7683 |
| YIYU8 | -0.6275 | 0.0969 | -6.4754 | 0 | 0.5339 | 0.4415 | 0.6456 |
| YUNDONG9 | -0.1298 | 0.1004 | -1.2934 | 0.1959 | 0.8782 | 0.7214 | 1.0692 |

Chi-square tests for linear terms

|  |  |  |  |
| --- | --- | --- | --- |
|  | df | Chi.sq | p-value |
| factor(DRINK10) | 1 | 5.4795 | 0.0192 |
| factor(ZHONGZU3) | 4 | 29.0877 | 0 |
| factor(XIYAN11) | 2 | 7.3581 | 0.0252 |
| GAOXUEYA12 | 1 | 5.7575 | 0.0164 |
| TANGNIAOBING13 | 1 | 0.0079 | 0.9291 |
| FEIBING14 | 1 | 1.4543 | 0.2278 |
| XINGZHANGBING15 | 1 | 7.7397 | 0.0054 |
| GANBING16 | 1 | 1.4093 | 0.2352 |
| DANBAIZHI17 | 1 | 2.8657 | 0.0905 |
| TANSHUI18 | 1 | 16.9938 | 0 |
| XIANWEI19 | 1 | 10.6245 | 0.0011 |
| ZHIFANG20 | 1 | 2.4212 | 0.1197 |
| SHUIFEN21 | 1 | 11.2217 | 8e-04 |
| NENGLIANG22 | 1 | 6.9631 | 0.0083 |
| XINBIE1 | 1 | 121.6371 | 0 |
| AGE2 | 1 | 6.131 | 0.0133 |
| factor(JIAOYU4) | 2 | 28.3356 | 0 |
| factor(HUNYING5) | 2 | 0.4551 | 0.7965 |
| PIR6 | 1 | 3.8552 | 0.0496 |
| factor(BMI7) | 2 | 26.6237 | 0 |
| YIYU8 | 1 | 41.9314 | 0 |
| YUNDONG9 | 1 | 1.673 | 0.1959 |

Approximate significance of smooth terms

|  |  |  |  |  |
| --- | --- | --- | --- | --- |
|  | edf | Ref.df | Chi.sq | p-value |
| s(CDAI23):factor(DRINK10)1 | 1.4042 | 1.7247 | 12.3804 | 0.0013 |
| s(CDAI23):factor(DRINK10)2 | 2.9298 | 3.7502 | 4.4524 | 0.3271 |

Model statistics

|  |  |
| --- | --- |
| N: | 10904 |
| Adj. r-square: | 0.0555 |
| Deviance explained: | 0.0809 |
| UBRE score (sp.criterion): | -0.3622 |
| Scale estimate: | 1 |
| family: | binomial |
| link function: | logit |
