# Supplementary material for: Association between the composite dietary antioxidant index and constipation: Evidence from NHANES 2005–2010
Source: PLoS One. 2024 Sep 27;19(9):e0311168. doi: 10.1371/journal.pone.0311168 (PMC11432863; doi:10.1371/journal.pone.0311168)
Supplement: S1 File — (ZIP) [file pone.0311168.s001.zip › CDAI/all/PROJ2_11_tbl/PROJ2_11_tbl.htm]

|  |
| --- |
| BIANMI24 vs. CDAI23 |

Generalize additive models
Outcome: BIANMI24
Exposure: CDAI23
Linear terms effect

|  |  |  |  |  |  |  |  |
| --- | --- | --- | --- | --- | --- | --- | --- |
|  | Estimate | Std. Error | z value | Pr(>|z|) | exp(est) | 95%CI low | 95%CI upp |
| (Intercept) | -1.3375 | 0.6415 | -2.085 | 0.0371 | 0.2625 | 0.0747 | 0.9229 |
| factor(XIYAN11)2 | -0.1898 | 0.1157 | -1.6398 | 0.1011 | 0.8271 | 0.6592 | 1.0378 |
| factor(XIYAN11)3 | 0.1214 | 0.0894 | 1.3572 | 0.1747 | 1.1291 | 0.9475 | 1.3454 |
| factor(ZHONGZU3)2 | 0.3076 | 0.1313 | 2.3423 | 0.0192 | 1.3602 | 1.0515 | 1.7596 |
| factor(ZHONGZU3)3 | 0.2182 | 0.105 | 2.0789 | 0.0376 | 1.2439 | 1.0126 | 1.528 |
| factor(ZHONGZU3)4 | 0.5572 | 0.1126 | 4.9495 | 0 | 1.7458 | 1.4001 | 2.1768 |
| factor(ZHONGZU3)5 | 0.1041 | 0.1938 | 0.5372 | 0.5911 | 1.1097 | 0.759 | 1.6224 |
| GAOXUEYA12 | 0.1852 | 0.0766 | 2.4198 | 0.0155 | 1.2035 | 1.0358 | 1.3983 |
| TANGNIAOBING13 | -0.0124 | 0.1012 | -0.1224 | 0.9026 | 0.9877 | 0.8099 | 1.2045 |
| FEIBING14 | -0.0987 | 0.0861 | -1.1464 | 0.2516 | 0.906 | 0.7653 | 1.0726 |
| XINGZHANGBING15 | -0.3316 | 0.1192 | -2.7811 | 0.0054 | 0.7178 | 0.5682 | 0.9067 |
| GANBING16 | 0.2236 | 0.1946 | 1.1488 | 0.2506 | 1.2505 | 0.854 | 1.8312 |
| DANBAIZHI17 | 0.0046 | 0.0026 | 1.7632 | 0.0779 | 1.0046 | 0.9995 | 1.0097 |
| TANSHUI18 | 0.0063 | 0.0015 | 4.1768 | 0 | 1.0063 | 1.0033 | 1.0093 |
| XIANWEI19 | -0.0209 | 0.0065 | -3.2189 | 0.0013 | 0.9793 | 0.9669 | 0.9919 |
| ZHIFANG20 | 0.0059 | 0.0037 | 1.617 | 0.1059 | 1.006 | 0.9987 | 1.0132 |
| SHUIFEN21 | -1e-04 | 0 | -3.3752 | 7e-04 | 0.9999 | 0.9998 | 0.9999 |
| NENGLIANG22 | -0.001 | 4e-04 | -2.6745 | 0.0075 | 0.999 | 0.9983 | 0.9997 |
| XINBIE1 | 0.8911 | 0.0803 | 11.0913 | 0 | 2.4378 | 2.0826 | 2.8535 |
| AGE2 | -0.0061 | 0.0026 | -2.3735 | 0.0176 | 0.9939 | 0.9889 | 0.9989 |
| factor(JIAOYU4)2 | -0.0537 | 0.0881 | -0.6098 | 0.542 | 0.9477 | 0.7973 | 1.1264 |
| factor(JIAOYU4)3 | -0.4041 | 0.0859 | -4.7068 | 0 | 0.6676 | 0.5642 | 0.7899 |
| factor(HUNYING5)2 | 0.0517 | 0.0824 | 0.6272 | 0.5305 | 1.053 | 0.896 | 1.2375 |
| factor(HUNYING5)3 | 0.0301 | 0.0933 | 0.3222 | 0.7473 | 1.0305 | 0.8582 | 1.2374 |
| PIR6 | -0.1369 | 0.0694 | -1.9724 | 0.0486 | 0.872 | 0.7611 | 0.9991 |
| factor(BMI7)2 | -0.1757 | 0.0799 | -2.1977 | 0.028 | 0.8389 | 0.7172 | 0.9812 |
| factor(BMI7)3 | -0.4155 | 0.0828 | -5.0165 | 0 | 0.66 | 0.5611 | 0.7763 |
| YIYU8 | -0.626 | 0.097 | -6.4547 | 0 | 0.5347 | 0.4421 | 0.6467 |
| YUNDONG9 | -0.1237 | 0.1003 | -1.2333 | 0.2175 | 0.8836 | 0.7259 | 1.0756 |
| DRINK10 | 0.1129 | 0.0727 | 1.5534 | 0.1203 | 1.1195 | 0.9709 | 1.2909 |

Chi-square tests for linear terms

|  |  |  |  |
| --- | --- | --- | --- |
|  | df | Chi.sq | p-value |
| factor(XIYAN11) | 2 | 9.8748 | 0.0072 |
| factor(ZHONGZU3) | 4 | 29.3926 | 0 |
| GAOXUEYA12 | 1 | 5.8556 | 0.0155 |
| TANGNIAOBING13 | 1 | 0.015 | 0.9026 |
| FEIBING14 | 1 | 1.3143 | 0.2516 |
| XINGZHANGBING15 | 1 | 7.7347 | 0.0054 |
| GANBING16 | 1 | 1.3197 | 0.2506 |
| DANBAIZHI17 | 1 | 3.109 | 0.0779 |
| TANSHUI18 | 1 | 17.4453 | 0 |
| XIANWEI19 | 1 | 10.3616 | 0.0013 |
| ZHIFANG20 | 1 | 2.6147 | 0.1059 |
| SHUIFEN21 | 1 | 11.3919 | 7e-04 |
| NENGLIANG22 | 1 | 7.1532 | 0.0075 |
| XINBIE1 | 1 | 123.016 | 0 |
| AGE2 | 1 | 5.6336 | 0.0176 |
| factor(JIAOYU4) | 2 | 28.4031 | 0 |
| factor(HUNYING5) | 2 | 0.4419 | 0.8017 |
| PIR6 | 1 | 3.8903 | 0.0486 |
| factor(BMI7) | 2 | 25.3368 | 0 |
| YIYU8 | 1 | 41.6636 | 0 |
| YUNDONG9 | 1 | 1.521 | 0.2175 |
| DRINK10 | 1 | 2.4131 | 0.1203 |

Approximate significance of smooth terms

|  |  |  |  |  |
| --- | --- | --- | --- | --- |
|  | edf | Ref.df | Chi.sq | p-value |
| s(CDAI23):factor(XIYAN11)1 | 1.0085 | 1.017 | 5.5393 | 0.019 |
| s(CDAI23):factor(XIYAN11)2 | 1.0005 | 1.0009 | 8.7879 | 0.003 |
| s(CDAI23):factor(XIYAN11)3 | 1.3391 | 1.6154 | 3.3404 | 0.211 |

Model statistics

|  |  |
| --- | --- |
| N: | 10904 |
| Adj. r-square: | 0.0544 |
| Deviance explained: | 0.0794 |
| UBRE score (sp.criterion): | -0.3613 |
| Scale estimate: | 1 |
| family: | binomial |
| link function: | logit |
