# Supplementary material for: Association between the composite dietary antioxidant index and constipation: Evidence from NHANES 2005–2010
Source: PLoS One. 2024 Sep 27;19(9):e0311168. doi: 10.1371/journal.pone.0311168 (PMC11432863; doi:10.1371/journal.pone.0311168)
Supplement: S1 File — (ZIP) [file pone.0311168.s001.zip › CDAI/all/PROJ2_12_tbl/PROJ2_12_tbl.htm]

|  |
| --- |
| BIANMI24 vs. CDAI23 |

Generalize additive models
Outcome: BIANMI24
Exposure: CDAI23
Linear terms effect

|  |  |  |  |  |  |  |  |
| --- | --- | --- | --- | --- | --- | --- | --- |
|  | Estimate | Std. Error | z value | Pr(>|z|) | exp(est) | 95%CI low | 95%CI upp |
| (Intercept) | -1.147 | 0.6302 | -1.8202 | 0.0687 | 0.3176 | 0.0924 | 1.0921 |
| factor(GAOXUEYA12)2 | 0.203 | 0.0791 | 2.5646 | 0.0103 | 1.225 | 1.049 | 1.4306 |
| factor(ZHONGZU3)2 | 0.3079 | 0.1314 | 2.3437 | 0.0191 | 1.3606 | 1.0517 | 1.7602 |
| factor(ZHONGZU3)3 | 0.2276 | 0.1049 | 2.1711 | 0.0299 | 1.2556 | 1.0224 | 1.5421 |
| factor(ZHONGZU3)4 | 0.5609 | 0.1126 | 4.9826 | 0 | 1.7522 | 1.4053 | 2.1847 |
| factor(ZHONGZU3)5 | 0.1075 | 0.1938 | 0.5548 | 0.579 | 1.1135 | 0.7616 | 1.6281 |
| TANGNIAOBING13 | -0.0099 | 0.1013 | -0.0977 | 0.9222 | 0.9902 | 0.8119 | 1.2076 |
| FEIBING14 | -0.1039 | 0.086 | -1.2086 | 0.2268 | 0.9013 | 0.7615 | 1.0667 |
| XINGZHANGBING15 | -0.3305 | 0.1192 | -2.7722 | 0.0056 | 0.7185 | 0.5688 | 0.9077 |
| GANBING16 | 0.2266 | 0.1945 | 1.1649 | 0.2441 | 1.2543 | 0.8567 | 1.8365 |
| DANBAIZHI17 | 0.0045 | 0.0026 | 1.7268 | 0.0842 | 1.0045 | 0.9994 | 1.0096 |
| TANSHUI18 | 0.0064 | 0.0015 | 4.2184 | 0 | 1.0064 | 1.0034 | 1.0094 |
| XIANWEI19 | -0.0208 | 0.0065 | -3.2014 | 0.0014 | 0.9795 | 0.9671 | 0.992 |
| ZHIFANG20 | 0.006 | 0.0037 | 1.6224 | 0.1047 | 1.006 | 0.9988 | 1.0132 |
| SHUIFEN21 | -1e-04 | 0 | -3.3212 | 9e-04 | 0.9999 | 0.9998 | 1 |
| NENGLIANG22 | -0.001 | 4e-04 | -2.7053 | 0.0068 | 0.999 | 0.9983 | 0.9997 |
| XINBIE1 | 0.8918 | 0.0804 | 11.0922 | 0 | 2.4395 | 2.0838 | 2.8558 |
| AGE2 | -0.0063 | 0.0026 | -2.4467 | 0.0144 | 0.9937 | 0.9887 | 0.9987 |
| factor(JIAOYU4)2 | -0.0514 | 0.0881 | -0.5832 | 0.5597 | 0.9499 | 0.7993 | 1.1289 |
| factor(JIAOYU4)3 | -0.3985 | 0.0858 | -4.6452 | 0 | 0.6713 | 0.5674 | 0.7943 |
| factor(HUNYING5)2 | 0.0511 | 0.0823 | 0.6212 | 0.5345 | 1.0525 | 0.8956 | 1.2368 |
| factor(HUNYING5)3 | 0.0236 | 0.0933 | 0.2529 | 0.8003 | 1.0239 | 0.8528 | 1.2293 |
| PIR6 | -0.1389 | 0.0694 | -2.0029 | 0.0452 | 0.8703 | 0.7597 | 0.997 |
| factor(BMI7)2 | -0.1774 | 0.0799 | -2.2196 | 0.0264 | 0.8375 | 0.716 | 0.9795 |
| factor(BMI7)3 | -0.4178 | 0.0828 | -5.0458 | 0 | 0.6585 | 0.5599 | 0.7745 |
| YIYU8 | -0.6271 | 0.0969 | -6.4738 | 0 | 0.5341 | 0.4418 | 0.6458 |
| YUNDONG9 | -0.1178 | 0.1003 | -1.1744 | 0.2402 | 0.8889 | 0.7303 | 1.082 |
| DRINK10 | 0.1104 | 0.0727 | 1.5173 | 0.1292 | 1.1167 | 0.9683 | 1.2878 |
| factor(XIYAN11)2 | -0.1457 | 0.1055 | -1.3807 | 0.1674 | 0.8644 | 0.7029 | 1.063 |
| factor(XIYAN11)3 | 0.0933 | 0.0865 | 1.079 | 0.2806 | 1.0978 | 0.9267 | 1.3005 |

Chi-square tests for linear terms

|  |  |  |  |
| --- | --- | --- | --- |
|  | df | Chi.sq | p-value |
| factor(GAOXUEYA12) | 1 | 6.5769 | 0.0103 |
| factor(ZHONGZU3) | 4 | 29.3504 | 0 |
| TANGNIAOBING13 | 1 | 0.0095 | 0.9222 |
| FEIBING14 | 1 | 1.4608 | 0.2268 |
| XINGZHANGBING15 | 1 | 7.6851 | 0.0056 |
| GANBING16 | 1 | 1.357 | 0.2441 |
| DANBAIZHI17 | 1 | 2.9817 | 0.0842 |
| TANSHUI18 | 1 | 17.7951 | 0 |
| XIANWEI19 | 1 | 10.2491 | 0.0014 |
| ZHIFANG20 | 1 | 2.6323 | 0.1047 |
| SHUIFEN21 | 1 | 11.0307 | 9e-04 |
| NENGLIANG22 | 1 | 7.3187 | 0.0068 |
| XINBIE1 | 1 | 123.0365 | 0 |
| AGE2 | 1 | 5.9863 | 0.0144 |
| factor(JIAOYU4) | 2 | 27.7807 | 0 |
| factor(HUNYING5) | 2 | 0.4087 | 0.8152 |
| PIR6 | 1 | 4.0117 | 0.0452 |
| factor(BMI7) | 2 | 25.6231 | 0 |
| YIYU8 | 1 | 41.9095 | 0 |
| YUNDONG9 | 1 | 1.3793 | 0.2402 |
| DRINK10 | 1 | 2.3021 | 0.1292 |
| factor(XIYAN11) | 2 | 6.9708 | 0.0306 |

Approximate significance of smooth terms

|  |  |  |  |  |
| --- | --- | --- | --- | --- |
|  | edf | Ref.df | Chi.sq | p-value |
| s(CDAI23):factor(GAOXUEYA12)1 | 1.0012 | 1.0024 | 7.7477 | 0.0054 |
| s(CDAI23):factor(GAOXUEYA12)2 | 1.0045 | 1.0089 | 3.9939 | 0.0465 |

Model statistics

|  |  |
| --- | --- |
| N: | 10904 |
| Adj. r-square: | 0.0538 |
| Deviance explained: | 0.0787 |
| UBRE score (sp.criterion): | -0.3611 |
| Scale estimate: | 1 |
| family: | binomial |
| link function: | logit |
