# Supplementary material for: Association between the composite dietary antioxidant index and constipation: Evidence from NHANES 2005–2010
Source: PLoS One. 2024 Sep 27;19(9):e0311168. doi: 10.1371/journal.pone.0311168 (PMC11432863; doi:10.1371/journal.pone.0311168)
Supplement: S1 File — (ZIP) [file pone.0311168.s001.zip › CDAI/all/PROJ2_13_tbl/PROJ2_13_tbl.htm]

|  |
| --- |
| BIANMI24 vs. CDAI23 |

Generalize additive models
Outcome: BIANMI24
Exposure: CDAI23
Linear terms effect

|  |  |  |  |  |  |  |  |
| --- | --- | --- | --- | --- | --- | --- | --- |
|  | Estimate | Std. Error | z value | Pr(>|z|) | exp(est) | 95%CI low | 95%CI upp |
| (Intercept) | -1.3675 | 0.6257 | -2.1854 | 0.0289 | 0.2548 | 0.0747 | 0.8685 |
| factor(TANGNIAOBING13)2 | 0.025 | 0.1121 | 0.223 | 0.8235 | 1.0253 | 0.8231 | 1.2772 |
| factor(ZHONGZU3)2 | 0.31 | 0.1314 | 2.3591 | 0.0183 | 1.3634 | 1.0538 | 1.7638 |
| factor(ZHONGZU3)3 | 0.2266 | 0.1049 | 2.1606 | 0.0307 | 1.2543 | 1.0213 | 1.5406 |
| factor(ZHONGZU3)4 | 0.5613 | 0.1126 | 4.9857 | 0 | 1.753 | 1.4059 | 2.1858 |
| factor(ZHONGZU3)5 | 0.1045 | 0.1939 | 0.539 | 0.5899 | 1.1101 | 0.7592 | 1.6233 |
| FEIBING14 | -0.1045 | 0.086 | -1.2153 | 0.2242 | 0.9008 | 0.761 | 1.0661 |
| XINGZHANGBING15 | -0.3304 | 0.1192 | -2.7718 | 0.0056 | 0.7186 | 0.5689 | 0.9078 |
| GANBING16 | 0.2287 | 0.1945 | 1.1758 | 0.2397 | 1.257 | 0.8585 | 1.8404 |
| DANBAIZHI17 | 0.0046 | 0.0026 | 1.7571 | 0.0789 | 1.0046 | 0.9995 | 1.0097 |
| TANSHUI18 | 0.0064 | 0.0015 | 4.2294 | 0 | 1.0064 | 1.0034 | 1.0094 |
| XIANWEI19 | -0.0209 | 0.0065 | -3.2152 | 0.0013 | 0.9794 | 0.967 | 0.9919 |
| ZHIFANG20 | 0.006 | 0.0037 | 1.6295 | 0.1032 | 1.006 | 0.9988 | 1.0133 |
| SHUIFEN21 | -1e-04 | 0 | -3.324 | 9e-04 | 0.9999 | 0.9998 | 1 |
| NENGLIANG22 | -0.001 | 4e-04 | -2.7162 | 0.0066 | 0.999 | 0.9983 | 0.9997 |
| XINBIE1 | 0.8919 | 0.0804 | 11.0928 | 0 | 2.4397 | 2.084 | 2.8561 |
| AGE2 | -0.0063 | 0.0026 | -2.4314 | 0.015 | 0.9938 | 0.9888 | 0.9988 |
| factor(JIAOYU4)2 | -0.0514 | 0.0881 | -0.5835 | 0.5595 | 0.9499 | 0.7992 | 1.1289 |
| factor(JIAOYU4)3 | -0.3988 | 0.0858 | -4.6487 | 0 | 0.6711 | 0.5673 | 0.794 |
| factor(HUNYING5)2 | 0.0528 | 0.0823 | 0.6413 | 0.5213 | 1.0542 | 0.8971 | 1.2388 |
| factor(HUNYING5)3 | 0.023 | 0.0933 | 0.2465 | 0.8053 | 1.0233 | 0.8522 | 1.2286 |
| PIR6 | -0.1386 | 0.0694 | -1.998 | 0.0457 | 0.8706 | 0.7599 | 0.9974 |
| factor(BMI7)2 | -0.1795 | 0.0799 | -2.2466 | 0.0247 | 0.8357 | 0.7145 | 0.9774 |
| factor(BMI7)3 | -0.4195 | 0.0827 | -5.0695 | 0 | 0.6574 | 0.559 | 0.7731 |
| YIYU8 | -0.6268 | 0.0969 | -6.47 | 0 | 0.5343 | 0.4419 | 0.646 |
| YUNDONG9 | -0.1186 | 0.1003 | -1.1823 | 0.2371 | 0.8882 | 0.7297 | 1.0811 |
| DRINK10 | 0.1104 | 0.0727 | 1.5178 | 0.1291 | 1.1167 | 0.9683 | 1.2878 |
| factor(XIYAN11)2 | -0.1455 | 0.1055 | -1.3792 | 0.1678 | 0.8646 | 0.703 | 1.0632 |
| factor(XIYAN11)3 | 0.0933 | 0.0864 | 1.0794 | 0.2804 | 1.0978 | 0.9267 | 1.3005 |
| GAOXUEYA12 | 0.1846 | 0.0765 | 2.4124 | 0.0158 | 1.2028 | 1.0352 | 1.3975 |

Chi-square tests for linear terms

|  |  |  |  |
| --- | --- | --- | --- |
|  | df | Chi.sq | p-value |
| factor(TANGNIAOBING13) | 1 | 0.0497 | 0.8235 |
| factor(ZHONGZU3) | 4 | 29.5087 | 0 |
| FEIBING14 | 1 | 1.477 | 0.2242 |
| XINGZHANGBING15 | 1 | 7.6826 | 0.0056 |
| GANBING16 | 1 | 1.3825 | 0.2397 |
| DANBAIZHI17 | 1 | 3.0874 | 0.0789 |
| TANSHUI18 | 1 | 17.8879 | 0 |
| XIANWEI19 | 1 | 10.3375 | 0.0013 |
| ZHIFANG20 | 1 | 2.6554 | 0.1032 |
| SHUIFEN21 | 1 | 11.0488 | 9e-04 |
| NENGLIANG22 | 1 | 7.3779 | 0.0066 |
| XINBIE1 | 1 | 123.0497 | 0 |
| AGE2 | 1 | 5.9117 | 0.015 |
| factor(JIAOYU4) | 2 | 27.8319 | 0 |
| factor(HUNYING5) | 2 | 0.4313 | 0.806 |
| PIR6 | 1 | 3.992 | 0.0457 |
| factor(BMI7) | 2 | 25.8472 | 0 |
| YIYU8 | 1 | 41.8611 | 0 |
| YUNDONG9 | 1 | 1.3978 | 0.2371 |
| DRINK10 | 1 | 2.3036 | 0.1291 |
| factor(XIYAN11) | 2 | 6.9619 | 0.0308 |
| GAOXUEYA12 | 1 | 5.8198 | 0.0158 |

Approximate significance of smooth terms

|  |  |  |  |  |
| --- | --- | --- | --- | --- |
|  | edf | Ref.df | Chi.sq | p-value |
| s(CDAI23):factor(TANGNIAOBING13)1 | 1.0012 | 1.0024 | 4.1535 | 0.0416 |
| s(CDAI23):factor(TANGNIAOBING13)2 | 1.0021 | 1.0043 | 6.4786 | 0.0111 |

Model statistics

|  |  |
| --- | --- |
| N: | 10904 |
| Adj. r-square: | 0.0538 |
| Deviance explained: | 0.0787 |
| UBRE score (sp.criterion): | -0.3611 |
| Scale estimate: | 1 |
| family: | binomial |
| link function: | logit |
