# Supplementary material for: Association between the composite dietary antioxidant index and constipation: Evidence from NHANES 2005–2010
Source: PLoS One. 2024 Sep 27;19(9):e0311168. doi: 10.1371/journal.pone.0311168 (PMC11432863; doi:10.1371/journal.pone.0311168)
Supplement: S1 File — (ZIP) [file pone.0311168.s001.zip › CDAI/all/PROJ2_14_tbl/PROJ2_14_tbl.htm]

|  |
| --- |
| BIANMI24 vs. CDAI23 |

Generalize additive models
Outcome: BIANMI24
Exposure: CDAI23
Linear terms effect

|  |  |  |  |  |  |  |  |
| --- | --- | --- | --- | --- | --- | --- | --- |
|  | Estimate | Std. Error | z value | Pr(>|z|) | exp(est) | 95%CI low | 95%CI upp |
| (Intercept) | -1.443 | 0.6315 | -2.2851 | 0.0223 | 0.2362 | 0.0685 | 0.8144 |
| factor(FEIBING14)2 | -0.0723 | 0.0933 | -0.7754 | 0.4381 | 0.9302 | 0.7747 | 1.1169 |
| factor(ZHONGZU3)2 | 0.3064 | 0.1314 | 2.3315 | 0.0197 | 1.3585 | 1.05 | 1.7576 |
| factor(ZHONGZU3)3 | 0.2236 | 0.1049 | 2.131 | 0.0331 | 1.2506 | 1.0181 | 1.5361 |
| factor(ZHONGZU3)4 | 0.5605 | 0.1126 | 4.9793 | 0 | 1.7516 | 1.4048 | 2.184 |
| factor(ZHONGZU3)5 | 0.1025 | 0.1939 | 0.5285 | 0.5971 | 1.1079 | 0.7577 | 1.62 |
| XINGZHANGBING15 | -0.3311 | 0.1192 | -2.7782 | 0.0055 | 0.7181 | 0.5685 | 0.9071 |
| GANBING16 | 0.2278 | 0.1945 | 1.1714 | 0.2415 | 1.2559 | 0.8578 | 1.8387 |
| DANBAIZHI17 | 0.0045 | 0.0026 | 1.7319 | 0.0833 | 1.0045 | 0.9994 | 1.0097 |
| TANSHUI18 | 0.0064 | 0.0015 | 4.2236 | 0 | 1.0064 | 1.0034 | 1.0094 |
| XIANWEI19 | -0.021 | 0.0065 | -3.2322 | 0.0012 | 0.9792 | 0.9669 | 0.9918 |
| ZHIFANG20 | 0.006 | 0.0037 | 1.6246 | 0.1042 | 1.006 | 0.9988 | 1.0133 |
| SHUIFEN21 | -1e-04 | 0 | -3.3283 | 9e-04 | 0.9999 | 0.9998 | 1 |
| NENGLIANG22 | -0.001 | 4e-04 | -2.7113 | 0.0067 | 0.999 | 0.9983 | 0.9997 |
| XINBIE1 | 0.8916 | 0.0804 | 11.0862 | 0 | 2.4391 | 2.0834 | 2.8556 |
| AGE2 | -0.0062 | 0.0026 | -2.4171 | 0.0156 | 0.9938 | 0.9888 | 0.9988 |
| factor(JIAOYU4)2 | -0.0517 | 0.0881 | -0.5864 | 0.5576 | 0.9497 | 0.7991 | 1.1286 |
| factor(JIAOYU4)3 | -0.3992 | 0.0857 | -4.6551 | 0 | 0.6709 | 0.5671 | 0.7937 |
| factor(HUNYING5)2 | 0.0513 | 0.0823 | 0.6232 | 0.5331 | 1.0526 | 0.8958 | 1.2369 |
| factor(HUNYING5)3 | 0.0248 | 0.0933 | 0.2661 | 0.7902 | 1.0251 | 0.8538 | 1.2309 |
| PIR6 | -0.1392 | 0.0694 | -2.0059 | 0.0449 | 0.8701 | 0.7595 | 0.9968 |
| factor(BMI7)2 | -0.1795 | 0.0799 | -2.2464 | 0.0247 | 0.8357 | 0.7146 | 0.9774 |
| factor(BMI7)3 | -0.4217 | 0.0828 | -5.096 | 0 | 0.6559 | 0.5577 | 0.7714 |
| YIYU8 | -0.6261 | 0.0969 | -6.461 | 0 | 0.5347 | 0.4422 | 0.6465 |
| YUNDONG9 | -0.1199 | 0.1003 | -1.1949 | 0.2321 | 0.8871 | 0.7287 | 1.0798 |
| DRINK10 | 0.1093 | 0.0727 | 1.5022 | 0.133 | 1.1154 | 0.9673 | 1.2863 |
| factor(XIYAN11)2 | -0.1444 | 0.1056 | -1.3673 | 0.1715 | 0.8656 | 0.7038 | 1.0646 |
| factor(XIYAN11)3 | 0.0964 | 0.0865 | 1.1145 | 0.2651 | 1.1011 | 0.9295 | 1.3045 |
| GAOXUEYA12 | 0.1848 | 0.0765 | 2.4142 | 0.0158 | 1.203 | 1.0354 | 1.3977 |
| TANGNIAOBING13 | -0.0113 | 0.1012 | -0.1116 | 0.9111 | 0.9888 | 0.8108 | 1.2058 |

Chi-square tests for linear terms

|  |  |  |  |
| --- | --- | --- | --- |
|  | df | Chi.sq | p-value |
| factor(FEIBING14) | 1 | 0.6012 | 0.4381 |
| factor(ZHONGZU3) | 4 | 29.5439 | 0 |
| XINGZHANGBING15 | 1 | 7.7187 | 0.0055 |
| GANBING16 | 1 | 1.3721 | 0.2415 |
| DANBAIZHI17 | 1 | 2.9993 | 0.0833 |
| TANSHUI18 | 1 | 17.8389 | 0 |
| XIANWEI19 | 1 | 10.4474 | 0.0012 |
| ZHIFANG20 | 1 | 2.6393 | 0.1042 |
| SHUIFEN21 | 1 | 11.0777 | 9e-04 |
| NENGLIANG22 | 1 | 7.3509 | 0.0067 |
| XINBIE1 | 1 | 122.9036 | 0 |
| AGE2 | 1 | 5.8422 | 0.0156 |
| factor(JIAOYU4) | 2 | 27.8878 | 0 |
| factor(HUNYING5) | 2 | 0.4154 | 0.8125 |
| PIR6 | 1 | 4.0238 | 0.0449 |
| factor(BMI7) | 2 | 26.1295 | 0 |
| YIYU8 | 1 | 41.7441 | 0 |
| YUNDONG9 | 1 | 1.4279 | 0.2321 |
| DRINK10 | 1 | 2.2567 | 0.133 |
| factor(XIYAN11) | 2 | 7.0773 | 0.0291 |
| GAOXUEYA12 | 1 | 5.8285 | 0.0158 |
| TANGNIAOBING13 | 1 | 0.0125 | 0.9111 |

Approximate significance of smooth terms

|  |  |  |  |  |
| --- | --- | --- | --- | --- |
|  | edf | Ref.df | Chi.sq | p-value |
| s(CDAI23):factor(FEIBING14)1 | 1.4523 | 1.8015 | 6.2139 | 0.0661 |
| s(CDAI23):factor(FEIBING14)2 | 1.0041 | 1.0082 | 5.8844 | 0.0155 |

Model statistics

|  |  |
| --- | --- |
| N: | 10904 |
| Adj. r-square: | 0.0539 |
| Deviance explained: | 0.0789 |
| UBRE score (sp.criterion): | -0.3611 |
| Scale estimate: | 1 |
| family: | binomial |
| link function: | logit |
