# Supplementary material for: Association between the composite dietary antioxidant index and constipation: Evidence from NHANES 2005–2010
Source: PLoS One. 2024 Sep 27;19(9):e0311168. doi: 10.1371/journal.pone.0311168 (PMC11432863; doi:10.1371/journal.pone.0311168)
Supplement: S1 File — (ZIP) [file pone.0311168.s001.zip › CDAI/all/PROJ2_15_tbl/PROJ2_15_tbl.htm]

## ½»»¥×÷ÓÃ¼ìÑé

|  |  |  |  |
| --- | --- | --- | --- |
| Model | XINGZHANGBING15: 1 | XINGZHANGBING15: 2 | P interaction |
| Crude | 0.837 (0.776, 0.901) <0.0001 | 0.904 (0.886, 0.923) <0.0001 | 0.0397 |
| Model II | 0.902 (0.837, 0.973) 0.0072 | 0.963 (0.934, 0.994) 0.0177 | 0.0723 |
| Model II\* | 0.895 (0.803, 0.996) 0.0426 | 0.964 (0.934, 0.995) 0.0213 | 0.1774 |

Results in table:
¦Â (95%CI) Pvalue / OR (95%CI) Pvalue
½á¹û±äÁ¿: BIANMI24
Î£ÏÕÒòËØ: CDAI23
Ð§Ó¦ÐÞÊÎÒò×Ó: XINGZHANGBING15
Model II µ÷ÕûÁË: GANBING16, DANBAIZHI17, TANSHUI18, XIANWEI19, ZHIFANG20, SHUIFEN21, NENGLIANG22, AGE2, XINBIE1, ZHONGZU3, JIAOYU4, HUNYING5, PIR6, BMI7, YIYU8, YUNDONG9, DRINK10, XIYAN11, GAOXUEYA12, TANGNIAOBING13, FEIBING14
Model II\* µ÷ÕûÁË: GANBING16, DANBAIZHI17, TANSHUI18, XIANWEI19, ZHIFANG20, SHUIFEN21, NENGLIANG22, AGE2, XINBIE1, ZHONGZU3, JIAOYU4, HUNYING5, PIR6, BMI7, YIYU8, YUNDONG9, DRINK10, XIYAN11, GAOXUEYA12, TANGNIAOBING13, FEIBING14 and the interaction terms for following variables: DANBAIZHI17, TANSHUI18, XIANWEI19, SHUIFEN21, NENGLIANG22
´Ë±íÓÃÒ×õÍ³¼ÆÈí¼þ (www.empowerstats.com) ºÍRÈí¼þÉú³É£¬Éú³ÉÈÕÆÚ£º 2024-06-24
¸÷Ä£ÐÍËùÓÃµÄÑù±¾Á¿

|  |  |  |  |  |  |
| --- | --- | --- | --- | --- | --- |
| Y | Strata | X | Model | 1 | 2 |
| BIANMI24 | Total | CDAI23 | Crude | 944 | 9960 |
| BIANMI24 | Total | CDAI23 | Model II | 944 | 9960 |
| BIANMI24 | Total | CDAI23 | Model II\* | 944 | 9960 |
