# Supplementary material for: Association between the composite dietary antioxidant index and constipation: Evidence from NHANES 2005–2010
Source: PLoS One. 2024 Sep 27;19(9):e0311168. doi: 10.1371/journal.pone.0311168 (PMC11432863; doi:10.1371/journal.pone.0311168)
Supplement: S1 File — (ZIP) [file pone.0311168.s001.zip › CDAI/all/PROJ2_16_tbl/PROJ2_16_tbl.htm]

## ½»»¥×÷ÓÃ¼ìÑé

|  |  |  |  |
| --- | --- | --- | --- |
| Model | GANBING16: 1 | GANBING16: 2 | P interaction |
| Crude | 0.870 (0.766, 0.989) 0.0334 | 0.900 (0.882, 0.918) <0.0001 | 0.6108 |
| Model II | 0.949 (0.835, 1.078) 0.4197 | 0.958 (0.929, 0.988) 0.0057 | 0.8793 |
| Model II\* | 0.975 (0.784, 1.212) 0.8164 | 0.958 (0.929, 0.988) 0.0058 | 0.8781 |

Results in table:
¦Â (95%CI) Pvalue / OR (95%CI) Pvalue
½á¹û±äÁ¿: BIANMI24
Î£ÏÕÒòËØ: CDAI23
Ð§Ó¦ÐÞÊÎÒò×Ó: GANBING16
Model II µ÷ÕûÁË: DANBAIZHI17, TANSHUI18, XIANWEI19, ZHIFANG20, SHUIFEN21, NENGLIANG22, AGE2, XINBIE1, ZHONGZU3, JIAOYU4, HUNYING5, PIR6, BMI7, YIYU8, YUNDONG9, DRINK10, XIYAN11, GAOXUEYA12, TANGNIAOBING13, FEIBING14, XINGZHANGBING15
Model II\* µ÷ÕûÁË: DANBAIZHI17, TANSHUI18, XIANWEI19, ZHIFANG20, SHUIFEN21, NENGLIANG22, AGE2, XINBIE1, ZHONGZU3, JIAOYU4, HUNYING5, PIR6, BMI7, YIYU8, YUNDONG9, DRINK10, XIYAN11, GAOXUEYA12, TANGNIAOBING13, FEIBING14, XINGZHANGBING15 and the interaction terms for following variables: DANBAIZHI17, TANSHUI18, XIANWEI19, ZHIFANG20, SHUIFEN21, NENGLIANG22, XINBIE1, ZHONGZU3, PIR6, BMI7, YIYU8
´Ë±íÓÃÒ×õÍ³¼ÆÈí¼þ (www.empowerstats.com) ºÍRÈí¼þÉú³É£¬Éú³ÉÈÕÆÚ£º 2024-06-24
¸÷Ä£ÐÍËùÓÃµÄÑù±¾Á¿

|  |  |  |  |  |  |
| --- | --- | --- | --- | --- | --- |
| Y | Strata | X | Model | 1 | 2 |
| BIANMI24 | Total | CDAI23 | Crude | 370 | 10534 |
| BIANMI24 | Total | CDAI23 | Model II | 370 | 10534 |
| BIANMI24 | Total | CDAI23 | Model II\* | 370 | 10534 |
