# Supplementary material for: Association between the composite dietary antioxidant index and constipation: Evidence from NHANES 2005–2010
Source: PLoS One. 2024 Sep 27;19(9):e0311168. doi: 10.1371/journal.pone.0311168 (PMC11432863; doi:10.1371/journal.pone.0311168)
Supplement: S1 File — (ZIP) [file pone.0311168.s001.zip › CDAI/all/PROJ2_1_tbl/PROJ2_1_tbl.htm]

|  |
| --- |
| BIANMI24 vs. CDAI23 |

Generalize additive models
Outcome: BIANMI24
Exposure: CDAI23
Linear terms effect

|  |  |  |  |  |  |  |  |
| --- | --- | --- | --- | --- | --- | --- | --- |
|  | Estimate | Std. Error | z value | Pr(>|z|) | exp(est) | 95%CI low | 95%CI upp |
| (Intercept) | -1.3225 | 0.6401 | -2.0662 | 0.0388 | 0.2665 | 0.076 | 0.9343 |
| XINBIE1 | 0.892 | 0.0804 | 11.094 | 0 | 2.44 | 2.0842 | 2.8564 |
| AGE2 | -0.0062 | 0.0026 | -2.4192 | 0.0156 | 0.9938 | 0.9888 | 0.9988 |
| factor(ZHONGZU3)2 | 0.3095 | 0.1314 | 2.3553 | 0.0185 | 1.3627 | 1.0533 | 1.763 |
| factor(ZHONGZU3)3 | 0.2269 | 0.1049 | 2.1636 | 0.0305 | 1.2547 | 1.0216 | 1.541 |
| factor(ZHONGZU3)4 | 0.5614 | 0.1126 | 4.9873 | 0 | 1.7531 | 1.406 | 2.1859 |
| factor(ZHONGZU3)5 | 0.1052 | 0.1938 | 0.5426 | 0.5874 | 1.1109 | 0.7598 | 1.6243 |
| factor(JIAOYU4)2 | -0.0522 | 0.0881 | -0.5927 | 0.5534 | 0.9491 | 0.7986 | 1.128 |
| factor(JIAOYU4)3 | -0.3987 | 0.0858 | -4.6491 | 0 | 0.6712 | 0.5674 | 0.7941 |
| factor(HUNYING5)2 | 0.0517 | 0.0823 | 0.6276 | 0.5303 | 1.053 | 0.8961 | 1.2374 |
| factor(HUNYING5)3 | 0.0238 | 0.0933 | 0.255 | 0.7987 | 1.0241 | 0.8529 | 1.2296 |
| PIR6 | -0.1394 | 0.0694 | -2.0094 | 0.0445 | 0.8699 | 0.7593 | 0.9966 |
| factor(BMI7)2 | -0.1791 | 0.0799 | -2.2421 | 0.025 | 0.836 | 0.7148 | 0.9777 |
| factor(BMI7)3 | -0.4202 | 0.0827 | -5.0781 | 0 | 0.6569 | 0.5586 | 0.7726 |
| YIYU8 | -0.6275 | 0.0969 | -6.4776 | 0 | 0.5339 | 0.4416 | 0.6456 |
| YUNDONG9 | -0.1195 | 0.1003 | -1.1919 | 0.2333 | 0.8874 | 0.729 | 1.0801 |
| DRINK10 | 0.11 | 0.0727 | 1.5119 | 0.1306 | 1.1162 | 0.9679 | 1.2872 |
| factor(XIYAN11)2 | -0.1454 | 0.1055 | -1.3777 | 0.1683 | 0.8647 | 0.7031 | 1.0634 |
| factor(XIYAN11)3 | 0.0949 | 0.0864 | 1.0984 | 0.272 | 1.0996 | 0.9282 | 1.3025 |
| GAOXUEYA12 | 0.1849 | 0.0765 | 2.4162 | 0.0157 | 1.2031 | 1.0355 | 1.3979 |
| TANGNIAOBING13 | -0.0116 | 0.1012 | -0.1142 | 0.9091 | 0.9885 | 0.8106 | 1.2054 |
| FEIBING14 | -0.1046 | 0.086 | -1.2164 | 0.2238 | 0.9007 | 0.761 | 1.066 |
| XINGZHANGBING15 | -0.3294 | 0.1191 | -2.7649 | 0.0057 | 0.7193 | 0.5695 | 0.9086 |
| GANBING16 | 0.2285 | 0.1945 | 1.175 | 0.24 | 1.2567 | 0.8584 | 1.8399 |
| DANBAIZHI17 | 0.0046 | 0.0026 | 1.7578 | 0.0788 | 1.0046 | 0.9995 | 1.0097 |
| TANSHUI18 | 0.0064 | 0.0015 | 4.2301 | 0 | 1.0064 | 1.0034 | 1.0094 |
| XIANWEI19 | -0.0209 | 0.0065 | -3.2186 | 0.0013 | 0.9793 | 0.967 | 0.9919 |
| ZHIFANG20 | 0.006 | 0.0037 | 1.6207 | 0.1051 | 1.006 | 0.9988 | 1.0132 |
| SHUIFEN21 | -1e-04 | 0 | -3.337 | 8e-04 | 0.9999 | 0.9998 | 0.9999 |
| NENGLIANG22 | -0.001 | 4e-04 | -2.7107 | 0.0067 | 0.999 | 0.9983 | 0.9997 |

Chi-square tests for linear terms

|  |  |  |  |
| --- | --- | --- | --- |
|  | df | Chi.sq | p-value |
| XINBIE1 | 1 | 123.0776 | 0 |
| AGE2 | 1 | 5.8523 | 0.0156 |
| factor(ZHONGZU3) | 4 | 29.5052 | 0 |
| factor(JIAOYU4) | 2 | 27.7772 | 0 |
| factor(HUNYING5) | 2 | 0.417 | 0.8118 |
| PIR6 | 1 | 4.0378 | 0.0445 |
| factor(BMI7) | 2 | 25.9437 | 0 |
| YIYU8 | 1 | 41.9596 | 0 |
| YUNDONG9 | 1 | 1.4205 | 0.2333 |
| DRINK10 | 1 | 2.2857 | 0.1306 |
| factor(XIYAN11) | 2 | 7.0585 | 0.0293 |
| GAOXUEYA12 | 1 | 5.8379 | 0.0157 |
| TANGNIAOBING13 | 1 | 0.013 | 0.9091 |
| FEIBING14 | 1 | 1.4797 | 0.2238 |
| XINGZHANGBING15 | 1 | 7.6445 | 0.0057 |
| GANBING16 | 1 | 1.3806 | 0.24 |
| DANBAIZHI17 | 1 | 3.09 | 0.0788 |
| TANSHUI18 | 1 | 17.894 | 0 |
| XIANWEI19 | 1 | 10.3592 | 0.0013 |
| ZHIFANG20 | 1 | 2.6268 | 0.1051 |
| SHUIFEN21 | 1 | 11.1357 | 8e-04 |
| NENGLIANG22 | 1 | 7.3477 | 0.0067 |

Approximate significance of smooth terms

|  |  |  |  |  |
| --- | --- | --- | --- | --- |
|  | edf | Ref.df | Chi.sq | p-value |
| s(CDAI23) | 1.0039 | 1.0078 | 7.7935 | 0.0054 |

Model statistics

|  |  |
| --- | --- |
| N: | 10904 |
| Adj. r-square: | 0.0539 |
| Deviance explained: | 0.0786 |
| UBRE score (sp.criterion): | -0.3612 |
| Scale estimate: | 1 |
| family: | binomial |
| link function: | logit |
