# Supplementary material for: Association between the composite dietary antioxidant index and constipation: Evidence from NHANES 2005–2010
Source: PLoS One. 2024 Sep 27;19(9):e0311168. doi: 10.1371/journal.pone.0311168 (PMC11432863; doi:10.1371/journal.pone.0311168)
Supplement: S1 File — (ZIP) [file pone.0311168.s001.zip › CDAI/all/PROJ2_2_tbl1/PROJ2_2_tbl1.htm]

## µ¥ÒòËØ·ÖÎö

|  |  |  |
| --- | --- | --- |
|  | Statistics | BIANMI24 |
| HULUOBUSU30 ËÄ·Ö×é | 1.500 ± 1.118 | 0.959 (0.901, 1.021) 0.19280 |

±íÖÐÊý¾Ý£º
¦Â (95%CI) Pvalue / OR (95%CI) Pvalue
½á¹û±äÁ¿: BIANMI24
±©Â¶±äÁ¿: HULUOBUSU30 ËÄ·Ö×é
µ÷Õû±äÁ¿: XINBIE1; AGE2; ZHONGZU3; JIAOYU4; HUNYING5; PIR6; BMI7; YIYU8; YUNDONG9; DRINK10; XIYAN11; GAOXUEYA12; TANGNIAOBING13; FEIBING14; XINGZHANGBING15; GANBING16; DANBAIZHI17; TANSHUI18; XIANWEI19; ZHIFANG20; SHUIFEN21; NENGLIANG22
´Ë±íÓÃÒ×õÍ³¼ÆÈí¼þ (www.empowerstats.com) ºÍRÈí¼þÉú³É£¬Éú³ÉÈÕÆÚ£º 2024-06-24
¸÷Ä£ÐÍËùÓÃµÄÑù±¾Á¿

|  |  |
| --- | --- |
| Exposure | BIANMI24 |
| HULUOBUSU30 ËÄ·Ö×é | 10904 |
