# Supplementary material for: Association between the composite dietary antioxidant index and constipation: Evidence from NHANES 2005–2010
Source: PLoS One. 2024 Sep 27;19(9):e0311168. doi: 10.1371/journal.pone.0311168 (PMC11432863; doi:10.1371/journal.pone.0311168)
Supplement: S1 File — (ZIP) [file pone.0311168.s001.zip › CDAI/all/PROJ2_2_tbl/PROJ2_2_tbl.htm]

|  |
| --- |
| BIANMI24 vs. CDAI23 |

Generalize additive models
Outcome: BIANMI24
Exposure: CDAI23
Linear terms effect

|  |  |  |  |  |  |  |  |
| --- | --- | --- | --- | --- | --- | --- | --- |
|  | Estimate | Std. Error | z value | Pr(>|z|) | exp(est) | 95%CI low | 95%CI upp |
| (Intercept) | -0.4818 | 0.6288 | -0.7662 | 0.4436 | 0.6177 | 0.1801 | 2.1184 |
| factor(XINBIE1)2 | 0.9391 | 0.0817 | 11.5018 | 0 | 2.5578 | 2.1795 | 3.0017 |
| AGE2 | -0.0063 | 0.0026 | -2.4559 | 0.0141 | 0.9937 | 0.9887 | 0.9987 |
| factor(ZHONGZU3)2 | 0.3076 | 0.1313 | 2.3419 | 0.0192 | 1.3601 | 1.0514 | 1.7594 |
| factor(ZHONGZU3)3 | 0.2277 | 0.1049 | 2.1712 | 0.0299 | 1.2557 | 1.0224 | 1.5422 |
| factor(ZHONGZU3)4 | 0.5598 | 0.1126 | 4.9724 | 0 | 1.7503 | 1.4037 | 2.1824 |
| factor(ZHONGZU3)5 | 0.103 | 0.1938 | 0.5312 | 0.5953 | 1.1085 | 0.7581 | 1.6208 |
| factor(JIAOYU4)2 | -0.0483 | 0.0881 | -0.5482 | 0.5836 | 0.9529 | 0.8018 | 1.1324 |
| factor(JIAOYU4)3 | -0.3971 | 0.0858 | -4.6288 | 0 | 0.6723 | 0.5682 | 0.7954 |
| factor(HUNYING5)2 | 0.0514 | 0.0822 | 0.625 | 0.532 | 1.0527 | 0.8961 | 1.2368 |
| factor(HUNYING5)3 | 0.0212 | 0.0933 | 0.2276 | 0.82 | 1.0215 | 0.8507 | 1.2265 |
| PIR6 | -0.1384 | 0.0694 | -1.9961 | 0.0459 | 0.8707 | 0.76 | 0.9975 |
| factor(BMI7)2 | -0.1753 | 0.0799 | -2.1935 | 0.0283 | 0.8392 | 0.7176 | 0.9815 |
| factor(BMI7)3 | -0.42 | 0.0827 | -5.076 | 0 | 0.6571 | 0.5587 | 0.7727 |
| YIYU8 | -0.6313 | 0.0968 | -6.5235 | 0 | 0.5319 | 0.44 | 0.643 |
| YUNDONG9 | -0.1228 | 0.1003 | -1.2237 | 0.2211 | 0.8845 | 0.7266 | 1.0767 |
| DRINK10 | 0.1152 | 0.0727 | 1.5853 | 0.1129 | 1.1221 | 0.9731 | 1.2939 |
| factor(XIYAN11)2 | -0.1454 | 0.1055 | -1.3788 | 0.168 | 0.8646 | 0.7032 | 1.0632 |
| factor(XIYAN11)3 | 0.0947 | 0.0864 | 1.0953 | 0.2734 | 1.0993 | 0.928 | 1.3023 |
| GAOXUEYA12 | 0.1835 | 0.0765 | 2.3994 | 0.0164 | 1.2014 | 1.0342 | 1.3957 |
| TANGNIAOBING13 | -0.0117 | 0.1012 | -0.1158 | 0.9078 | 0.9884 | 0.8106 | 1.2051 |
| FEIBING14 | -0.1052 | 0.086 | -1.2242 | 0.2209 | 0.9001 | 0.7606 | 1.0653 |
| XINGZHANGBING15 | -0.3278 | 0.1191 | -2.7528 | 0.0059 | 0.7205 | 0.5705 | 0.9099 |
| GANBING16 | 0.2218 | 0.1946 | 1.1398 | 0.2544 | 1.2483 | 0.8525 | 1.8278 |
| DANBAIZHI17 | 0.0051 | 0.0026 | 1.9448 | 0.0518 | 1.0051 | 1 | 1.0102 |
| TANSHUI18 | 0.0064 | 0.0015 | 4.2751 | 0 | 1.0065 | 1.0035 | 1.0094 |
| XIANWEI19 | -0.0208 | 0.0065 | -3.1993 | 0.0014 | 0.9794 | 0.967 | 0.992 |
| ZHIFANG20 | 0.006 | 0.0037 | 1.6378 | 0.1015 | 1.006 | 0.9988 | 1.0133 |
| SHUIFEN21 | -1e-04 | 0 | -3.2945 | 0.001 | 0.9999 | 0.9998 | 1 |
| NENGLIANG22 | -0.001 | 4e-04 | -2.7213 | 0.0065 | 0.999 | 0.9983 | 0.9997 |

Chi-square tests for linear terms

|  |  |  |  |
| --- | --- | --- | --- |
|  | df | Chi.sq | p-value |
| factor(XINBIE1) | 1 | 132.2912 | 0 |
| AGE2 | 1 | 6.0316 | 0.0141 |
| factor(ZHONGZU3) | 4 | 29.2674 | 0 |
| factor(JIAOYU4) | 2 | 27.7786 | 0 |
| factor(HUNYING5) | 2 | 0.4062 | 0.8162 |
| PIR6 | 1 | 3.9844 | 0.0459 |
| factor(BMI7) | 2 | 25.9674 | 0 |
| YIYU8 | 1 | 42.5566 | 0 |
| YUNDONG9 | 1 | 1.4974 | 0.2211 |
| DRINK10 | 1 | 2.513 | 0.1129 |
| factor(XIYAN11) | 2 | 7.0412 | 0.0296 |
| GAOXUEYA12 | 1 | 5.7571 | 0.0164 |
| TANGNIAOBING13 | 1 | 0.0134 | 0.9078 |
| FEIBING14 | 1 | 1.4988 | 0.2209 |
| XINGZHANGBING15 | 1 | 7.5781 | 0.0059 |
| GANBING16 | 1 | 1.2991 | 0.2544 |
| DANBAIZHI17 | 1 | 3.7824 | 0.0518 |
| TANSHUI18 | 1 | 18.2764 | 0 |
| XIANWEI19 | 1 | 10.2357 | 0.0014 |
| ZHIFANG20 | 1 | 2.6822 | 0.1015 |
| SHUIFEN21 | 1 | 10.8535 | 0.001 |
| NENGLIANG22 | 1 | 7.4053 | 0.0065 |

Approximate significance of smooth terms

|  |  |  |  |  |
| --- | --- | --- | --- | --- |
|  | edf | Ref.df | Chi.sq | p-value |
| s(CDAI23):factor(XINBIE1)1 | 1.0034 | 1.0067 | 16.0568 | 1e-04 |
| s(CDAI23):factor(XINBIE1)2 | 1.0009 | 1.0018 | 2.2394 | 0.1348 |

Model statistics

|  |  |
| --- | --- |
| N: | 10904 |
| Adj. r-square: | 0.0547 |
| Deviance explained: | 0.0799 |
| UBRE score (sp.criterion): | -0.3619 |
| Scale estimate: | 1 |
| family: | binomial |
| link function: | logit |
