# Supplementary material for: Association between the composite dietary antioxidant index and constipation: Evidence from NHANES 2005–2010
Source: PLoS One. 2024 Sep 27;19(9):e0311168. doi: 10.1371/journal.pone.0311168 (PMC11432863; doi:10.1371/journal.pone.0311168)
Supplement: S1 File — (ZIP) [file pone.0311168.s001.zip › CDAI/all/PROJ2_3_tbl1/PROJ2_3_tbl1.htm]

|  |
| --- |
| BIANMI24 vs. CDAI23 |

Generalize additive models
Outcome: BIANMI24
Exposure: CDAI23
Linear terms effect

|  |  |  |  |  |  |  |  |
| --- | --- | --- | --- | --- | --- | --- | --- |
|  | Estimate | Std. Error | z value | Pr(>|z|) | exp(est) | 95%CI low | 95%CI upp |
| (Intercept) | -1.5896 | 0.6147 | -2.5859 | 0.0097 | 0.204 | 0.0611 | 0.6806 |
| factor(AGE)2 | -0.0339 | 0.0884 | -0.3832 | 0.7015 | 0.9667 | 0.8129 | 1.1495 |
| factor(AGE)3 | -0.2273 | 0.1108 | -2.0509 | 0.0403 | 0.7967 | 0.6411 | 0.99 |
| factor(ZHONGZU3)2 | 0.3063 | 0.1314 | 2.3317 | 0.0197 | 1.3584 | 1.05 | 1.7574 |
| factor(ZHONGZU3)3 | 0.2106 | 0.1043 | 2.0203 | 0.0434 | 1.2344 | 1.0063 | 1.5143 |
| factor(ZHONGZU3)4 | 0.5545 | 0.1125 | 4.9285 | 0 | 1.7411 | 1.3965 | 2.1707 |
| factor(ZHONGZU3)5 | 0.1026 | 0.1939 | 0.5289 | 0.5969 | 1.108 | 0.7576 | 1.6204 |
| factor(JIAOYU4)2 | -0.0523 | 0.0881 | -0.5937 | 0.5527 | 0.949 | 0.7984 | 1.128 |
| factor(JIAOYU4)3 | -0.3959 | 0.0856 | -4.6263 | 0 | 0.6731 | 0.5691 | 0.796 |
| factor(HUNYING5)2 | 0.0457 | 0.0817 | 0.5592 | 0.576 | 1.0467 | 0.8919 | 1.2285 |
| factor(HUNYING5)3 | 0.0471 | 0.0928 | 0.5077 | 0.6117 | 1.0482 | 0.8739 | 1.2574 |
| PIR6 | -0.1446 | 0.0695 | -2.0815 | 0.0374 | 0.8653 | 0.7552 | 0.9916 |
| factor(BMI7)2 | -0.1873 | 0.08 | -2.3429 | 0.0191 | 0.8292 | 0.7089 | 0.9698 |
| factor(BMI7)3 | -0.427 | 0.0829 | -5.1498 | 0 | 0.6524 | 0.5546 | 0.7676 |
| YIYU8 | -0.6228 | 0.0972 | -6.4072 | 0 | 0.5364 | 0.4434 | 0.649 |
| YUNDONG9 | -0.1199 | 0.1003 | -1.1956 | 0.2319 | 0.887 | 0.7286 | 1.0797 |
| DRINK10 | 0.1086 | 0.0727 | 1.4929 | 0.1355 | 1.1147 | 0.9666 | 1.2855 |
| factor(XIYAN11)2 | -0.1491 | 0.1056 | -1.4117 | 0.158 | 0.8615 | 0.7004 | 1.0596 |
| factor(XIYAN11)3 | 0.0943 | 0.0865 | 1.0893 | 0.276 | 1.0988 | 0.9274 | 1.3019 |
| GAOXUEYA12 | 0.1913 | 0.0757 | 2.528 | 0.0115 | 1.2108 | 1.0439 | 1.4044 |
| TANGNIAOBING13 | -0.0132 | 0.1012 | -0.1303 | 0.8963 | 0.9869 | 0.8094 | 1.2033 |
| FEIBING14 | -0.1095 | 0.086 | -1.2729 | 0.203 | 0.8963 | 0.7573 | 1.0609 |
| XINGZHANGBING15 | -0.3296 | 0.1191 | -2.7666 | 0.0057 | 0.7192 | 0.5695 | 0.9084 |
| GANBING16 | 0.2408 | 0.1946 | 1.237 | 0.2161 | 1.2722 | 0.8687 | 1.8631 |
| DANBAIZHI17 | 0.0048 | 0.0026 | 1.8576 | 0.0632 | 1.0048 | 0.9997 | 1.01 |
| TANSHUI18 | 0.0064 | 0.0015 | 4.2647 | 0 | 1.0064 | 1.0035 | 1.0094 |
| XIANWEI19 | -0.0214 | 0.0065 | -3.3182 | 9e-04 | 0.9788 | 0.9665 | 0.9913 |
| ZHIFANG20 | 0.0059 | 0.0037 | 1.5952 | 0.1107 | 1.0059 | 0.9987 | 1.0131 |
| SHUIFEN21 | -1e-04 | 0 | -3.3579 | 8e-04 | 0.9999 | 0.9998 | 0.9999 |
| NENGLIANG22 | -0.001 | 4e-04 | -2.6853 | 0.0072 | 0.999 | 0.9983 | 0.9997 |
| XINBIE1 | 0.8964 | 0.0804 | 11.1518 | 0 | 2.4507 | 2.0935 | 2.8689 |

Chi-square tests for linear terms

|  |  |  |  |
| --- | --- | --- | --- |
|  | df | Chi.sq | p-value |
| factor(AGE) | 2 | 5.1763 | 0.0752 |
| factor(ZHONGZU3) | 4 | 29.4082 | 0 |
| factor(JIAOYU4) | 2 | 27.4568 | 0 |
| factor(HUNYING5) | 2 | 0.4773 | 0.7877 |
| PIR6 | 1 | 4.3326 | 0.0374 |
| factor(BMI7) | 2 | 26.6284 | 0 |
| YIYU8 | 1 | 41.0528 | 0 |
| YUNDONG9 | 1 | 1.4295 | 0.2319 |
| DRINK10 | 1 | 2.2289 | 0.1355 |
| factor(XIYAN11) | 2 | 7.1618 | 0.0279 |
| GAOXUEYA12 | 1 | 6.3906 | 0.0115 |
| TANGNIAOBING13 | 1 | 0.017 | 0.8963 |
| FEIBING14 | 1 | 1.6204 | 0.203 |
| XINGZHANGBING15 | 1 | 7.654 | 0.0057 |
| GANBING16 | 1 | 1.5302 | 0.2161 |
| DANBAIZHI17 | 1 | 3.4507 | 0.0632 |
| TANSHUI18 | 1 | 18.1881 | 0 |
| XIANWEI19 | 1 | 11.0105 | 9e-04 |
| ZHIFANG20 | 1 | 2.5446 | 0.1107 |
| SHUIFEN21 | 1 | 11.2752 | 8e-04 |
| NENGLIANG22 | 1 | 7.211 | 0.0072 |
| XINBIE1 | 1 | 124.3616 | 0 |

Approximate significance of smooth terms

|  |  |  |  |  |
| --- | --- | --- | --- | --- |
|  | edf | Ref.df | Chi.sq | p-value |
| s(CDAI23):factor(AGE)1 | 1.5128 | 1.8908 | 9.2071 | 0.0067 |
| s(CDAI23):factor(AGE)2 | 1.0012 | 1.0025 | 1.8374 | 0.176 |
| s(CDAI23):factor(AGE)3 | 1.0725 | 1.1421 | 4.3201 | 0.0551 |

Model statistics

|  |  |
| --- | --- |
| N: | 10904 |
| Adj. r-square: | 0.0542 |
| Deviance explained: | 0.0792 |
| UBRE score (sp.criterion): | -0.361 |
| Scale estimate: | 1 |
| family: | binomial |
| link function: | logit |
