# Supplementary material for: Association between the composite dietary antioxidant index and constipation: Evidence from NHANES 2005–2010
Source: PLoS One. 2024 Sep 27;19(9):e0311168. doi: 10.1371/journal.pone.0311168 (PMC11432863; doi:10.1371/journal.pone.0311168)
Supplement: S1 File — (ZIP) [file pone.0311168.s001.zip › CDAI/all/PROJ2_3_tbl/PROJ2_3_tbl.htm]

|  |
| --- |
| BIANMI24 vs. CDAI23 |

Generalize additive models
Outcome: BIANMI24
Exposure: CDAI23
Linear terms effect

|  |  |  |  |  |  |  |  |
| --- | --- | --- | --- | --- | --- | --- | --- |
|  | Estimate | Std. Error | z value | Pr(>|z|) | exp(est) | 95%CI low | 95%CI upp |
| (Intercept) | -1.4405 | 0.6526 | -2.2074 | 0.0273 | 0.2368 | 0.0659 | 0.8509 |
| factor(AGE)2 | 0.0304 | 0.1298 | 0.2341 | 0.8149 | 1.0309 | 0.7993 | 1.3295 |
| factor(AGE)3 | -0.0921 | 0.2286 | -0.4029 | 0.687 | 0.912 | 0.5827 | 1.4274 |
| AGE2 | -0.0037 | 0.0055 | -0.6763 | 0.4988 | 0.9963 | 0.9856 | 1.0071 |
| factor(ZHONGZU3)2 | 0.3098 | 0.1315 | 2.356 | 0.0185 | 1.3631 | 1.0535 | 1.7638 |
| factor(ZHONGZU3)3 | 0.2209 | 0.1054 | 2.0968 | 0.036 | 1.2472 | 1.0145 | 1.5333 |
| factor(ZHONGZU3)4 | 0.5585 | 0.1127 | 4.9568 | 0 | 1.7481 | 1.4017 | 2.1801 |
| factor(ZHONGZU3)5 | 0.1044 | 0.194 | 0.5381 | 0.5905 | 1.11 | 0.7589 | 1.6236 |
| factor(JIAOYU4)2 | -0.0541 | 0.0882 | -0.6133 | 0.5397 | 0.9473 | 0.797 | 1.1261 |
| factor(JIAOYU4)3 | -0.4002 | 0.0858 | -4.6631 | 0 | 0.6702 | 0.5664 | 0.7929 |
| factor(HUNYING5)2 | 0.0525 | 0.0823 | 0.6383 | 0.5233 | 1.0539 | 0.8969 | 1.2384 |
| factor(HUNYING5)3 | 0.0359 | 0.0943 | 0.3806 | 0.7035 | 1.0366 | 0.8616 | 1.2471 |
| PIR6 | -0.1425 | 0.0696 | -2.0482 | 0.0405 | 0.8672 | 0.7567 | 0.9939 |
| factor(BMI7)2 | -0.1855 | 0.08 | -2.3186 | 0.0204 | 0.8306 | 0.7101 | 0.9717 |
| factor(BMI7)3 | -0.4271 | 0.0829 | -5.1493 | 0 | 0.6524 | 0.5545 | 0.7676 |
| YIYU8 | -0.6224 | 0.0972 | -6.4035 | 0 | 0.5366 | 0.4436 | 0.6493 |
| YUNDONG9 | -0.1183 | 0.1003 | -1.1792 | 0.2383 | 0.8884 | 0.7298 | 1.0815 |
| DRINK10 | 0.1103 | 0.0728 | 1.515 | 0.1298 | 1.1166 | 0.9681 | 1.2878 |
| factor(XIYAN11)2 | -0.1534 | 0.1058 | -1.4497 | 0.1471 | 0.8578 | 0.6972 | 1.0555 |
| factor(XIYAN11)3 | 0.0928 | 0.0865 | 1.0723 | 0.2836 | 1.0973 | 0.9261 | 1.3001 |
| GAOXUEYA12 | 0.1839 | 0.0765 | 2.4038 | 0.0162 | 1.2019 | 1.0345 | 1.3963 |
| TANGNIAOBING13 | -0.0158 | 0.1013 | -0.1562 | 0.8758 | 0.9843 | 0.8071 | 1.2004 |
| FEIBING14 | -0.1071 | 0.0861 | -1.2444 | 0.2134 | 0.8984 | 0.7589 | 1.0635 |
| XINGZHANGBING15 | -0.3361 | 0.1195 | -2.8117 | 0.0049 | 0.7146 | 0.5653 | 0.9032 |
| GANBING16 | 0.2393 | 0.1946 | 1.2293 | 0.2189 | 1.2703 | 0.8674 | 1.8603 |
| DANBAIZHI17 | 0.0048 | 0.0026 | 1.8275 | 0.0676 | 1.0048 | 0.9997 | 1.0099 |
| TANSHUI18 | 0.0064 | 0.0015 | 4.2497 | 0 | 1.0064 | 1.0035 | 1.0094 |
| XIANWEI19 | -0.0211 | 0.0065 | -3.2515 | 0.0011 | 0.9791 | 0.9668 | 0.9917 |
| ZHIFANG20 | 0.0059 | 0.0037 | 1.5989 | 0.1098 | 1.0059 | 0.9987 | 1.0131 |
| SHUIFEN21 | -1e-04 | 0 | -3.3848 | 7e-04 | 0.9999 | 0.9998 | 0.9999 |
| NENGLIANG22 | -0.001 | 4e-04 | -2.6897 | 0.0072 | 0.999 | 0.9983 | 0.9997 |
| XINBIE1 | 0.8928 | 0.0805 | 11.0845 | 0 | 2.4419 | 2.0853 | 2.8595 |

Chi-square tests for linear terms

|  |  |  |  |
| --- | --- | --- | --- |
|  | df | Chi.sq | p-value |
| factor(AGE) | 2 | 1.2122 | 0.5455 |
| AGE2 | 1 | 0.4574 | 0.4988 |
| factor(ZHONGZU3) | 4 | 29.3926 | 0 |
| factor(JIAOYU4) | 2 | 27.8234 | 0 |
| factor(HUNYING5) | 2 | 0.4838 | 0.7852 |
| PIR6 | 1 | 4.1951 | 0.0405 |
| factor(BMI7) | 2 | 26.6418 | 0 |
| YIYU8 | 1 | 41.0048 | 0 |
| YUNDONG9 | 1 | 1.3906 | 0.2383 |
| DRINK10 | 1 | 2.2953 | 0.1298 |
| factor(XIYAN11) | 2 | 7.2989 | 0.026 |
| GAOXUEYA12 | 1 | 5.7785 | 0.0162 |
| TANGNIAOBING13 | 1 | 0.0244 | 0.8758 |
| FEIBING14 | 1 | 1.5485 | 0.2134 |
| XINGZHANGBING15 | 1 | 7.9056 | 0.0049 |
| GANBING16 | 1 | 1.5112 | 0.2189 |
| DANBAIZHI17 | 1 | 3.3397 | 0.0676 |
| TANSHUI18 | 1 | 18.06 | 0 |
| XIANWEI19 | 1 | 10.5723 | 0.0011 |
| ZHIFANG20 | 1 | 2.5564 | 0.1098 |
| SHUIFEN21 | 1 | 11.4567 | 7e-04 |
| NENGLIANG22 | 1 | 7.2343 | 0.0072 |
| XINBIE1 | 1 | 122.8664 | 0 |

Approximate significance of smooth terms

|  |  |  |  |  |
| --- | --- | --- | --- | --- |
|  | edf | Ref.df | Chi.sq | p-value |
| s(CDAI23):factor(AGE)1 | 1.4988 | 1.8694 | 8.8472 | 0.0078 |
| s(CDAI23):factor(AGE)2 | 1.001 | 1.0019 | 1.7945 | 0.1809 |
| s(CDAI23):factor(AGE)3 | 1.084 | 1.164 | 4.3276 | 0.058 |

Model statistics

|  |  |
| --- | --- |
| N: | 10904 |
| Adj. r-square: | 0.0543 |
| Deviance explained: | 0.0792 |
| UBRE score (sp.criterion): | -0.3608 |
| Scale estimate: | 1 |
| family: | binomial |
| link function: | logit |
