# Supplementary material for: Association between the composite dietary antioxidant index and constipation: Evidence from NHANES 2005–2010
Source: PLoS One. 2024 Sep 27;19(9):e0311168. doi: 10.1371/journal.pone.0311168 (PMC11432863; doi:10.1371/journal.pone.0311168)
Supplement: S1 File — (ZIP) [file pone.0311168.s001.zip › CDAI/all/PROJ2_4_tbl/PROJ2_4_tbl.htm]

|  |
| --- |
| BIANMI24 vs. CDAI23 |

Generalize additive models
Outcome: BIANMI24
Exposure: CDAI23
Linear terms effect

|  |  |  |  |  |  |  |  |
| --- | --- | --- | --- | --- | --- | --- | --- |
|  | Estimate | Std. Error | z value | Pr(>|z|) | exp(est) | 95%CI low | 95%CI upp |
| (Intercept) | -1.3095 | 0.6414 | -2.0415 | 0.0412 | 0.27 | 0.0768 | 0.9491 |
| factor(JIAOYU4)2 | -0.0415 | 0.0962 | -0.4317 | 0.6659 | 0.9593 | 0.7946 | 1.1583 |
| factor(JIAOYU4)3 | -0.3812 | 0.0911 | -4.1849 | 0 | 0.683 | 0.5713 | 0.8165 |
| factor(ZHONGZU3)2 | 0.3096 | 0.1314 | 2.3558 | 0.0185 | 1.3629 | 1.0534 | 1.7633 |
| factor(ZHONGZU3)3 | 0.2242 | 0.1049 | 2.1361 | 0.0327 | 1.2513 | 1.0186 | 1.5371 |
| factor(ZHONGZU3)4 | 0.5629 | 0.1126 | 4.999 | 0 | 1.7558 | 1.4081 | 2.1895 |
| factor(ZHONGZU3)5 | 0.0985 | 0.194 | 0.5079 | 0.6116 | 1.1035 | 0.7545 | 1.6141 |
| factor(HUNYING5)2 | 0.0516 | 0.0823 | 0.6263 | 0.5311 | 1.0529 | 0.896 | 1.2374 |
| factor(HUNYING5)3 | 0.0243 | 0.0933 | 0.2608 | 0.7943 | 1.0246 | 0.8534 | 1.2303 |
| PIR6 | -0.1414 | 0.0694 | -2.0355 | 0.0418 | 0.8682 | 0.7577 | 0.9948 |
| factor(BMI7)2 | -0.1795 | 0.08 | -2.2441 | 0.0248 | 0.8357 | 0.7144 | 0.9775 |
| factor(BMI7)3 | -0.4205 | 0.0828 | -5.079 | 0 | 0.6567 | 0.5583 | 0.7724 |
| YIYU8 | -0.6304 | 0.097 | -6.5016 | 0 | 0.5324 | 0.4402 | 0.6438 |
| YUNDONG9 | -0.1207 | 0.1003 | -1.2034 | 0.2288 | 0.8863 | 0.7282 | 1.0788 |
| DRINK10 | 0.111 | 0.0728 | 1.5255 | 0.1271 | 1.1174 | 0.9689 | 1.2886 |
| factor(XIYAN11)2 | -0.1454 | 0.1056 | -1.3764 | 0.1687 | 0.8647 | 0.703 | 1.0636 |
| factor(XIYAN11)3 | 0.0922 | 0.0865 | 1.0668 | 0.2861 | 1.0966 | 0.9257 | 1.2991 |
| GAOXUEYA12 | 0.185 | 0.0765 | 2.4173 | 0.0156 | 1.2032 | 1.0356 | 1.398 |
| TANGNIAOBING13 | -0.0108 | 0.1013 | -0.1067 | 0.915 | 0.9893 | 0.8112 | 1.2064 |
| FEIBING14 | -0.1042 | 0.086 | -1.2114 | 0.2257 | 0.901 | 0.7613 | 1.0665 |
| XINGZHANGBING15 | -0.3312 | 0.1192 | -2.7784 | 0.0055 | 0.7181 | 0.5684 | 0.907 |
| GANBING16 | 0.2272 | 0.1946 | 1.1676 | 0.243 | 1.2551 | 0.8571 | 1.8378 |
| DANBAIZHI17 | 0.0046 | 0.0026 | 1.7669 | 0.0772 | 1.0046 | 0.9995 | 1.0098 |
| TANSHUI18 | 0.0064 | 0.0015 | 4.2489 | 0 | 1.0064 | 1.0035 | 1.0094 |
| XIANWEI19 | -0.0212 | 0.0065 | -3.2541 | 0.0011 | 0.9791 | 0.9667 | 0.9916 |
| ZHIFANG20 | 0.006 | 0.0037 | 1.6294 | 0.1032 | 1.006 | 0.9988 | 1.0133 |
| SHUIFEN21 | -1e-04 | 0 | -3.325 | 9e-04 | 0.9999 | 0.9998 | 1 |
| NENGLIANG22 | -0.001 | 4e-04 | -2.7354 | 0.0062 | 0.999 | 0.9983 | 0.9997 |
| XINBIE1 | 0.8913 | 0.0804 | 11.0815 | 0 | 2.4384 | 2.0827 | 2.8547 |
| AGE2 | -0.0063 | 0.0026 | -2.4396 | 0.0147 | 0.9937 | 0.9887 | 0.9988 |

Chi-square tests for linear terms

|  |  |  |  |
| --- | --- | --- | --- |
|  | df | Chi.sq | p-value |
| factor(JIAOYU4) | 2 | 24.2206 | 0 |
| factor(ZHONGZU3) | 4 | 29.852 | 0 |
| factor(HUNYING5) | 2 | 0.4173 | 0.8117 |
| PIR6 | 1 | 4.1432 | 0.0418 |
| factor(BMI7) | 2 | 25.9532 | 0 |
| YIYU8 | 1 | 42.2712 | 0 |
| YUNDONG9 | 1 | 1.4482 | 0.2288 |
| DRINK10 | 1 | 2.3271 | 0.1271 |
| factor(XIYAN11) | 2 | 6.8732 | 0.0322 |
| GAOXUEYA12 | 1 | 5.8433 | 0.0156 |
| TANGNIAOBING13 | 1 | 0.0114 | 0.915 |
| FEIBING14 | 1 | 1.4676 | 0.2257 |
| XINGZHANGBING15 | 1 | 7.7195 | 0.0055 |
| GANBING16 | 1 | 1.3634 | 0.243 |
| DANBAIZHI17 | 1 | 3.1221 | 0.0772 |
| TANSHUI18 | 1 | 18.0529 | 0 |
| XIANWEI19 | 1 | 10.5891 | 0.0011 |
| ZHIFANG20 | 1 | 2.655 | 0.1032 |
| SHUIFEN21 | 1 | 11.0554 | 9e-04 |
| NENGLIANG22 | 1 | 7.4822 | 0.0062 |
| XINBIE1 | 1 | 122.7993 | 0 |
| AGE2 | 1 | 5.9514 | 0.0147 |

Approximate significance of smooth terms

|  |  |  |  |  |
| --- | --- | --- | --- | --- |
|  | edf | Ref.df | Chi.sq | p-value |
| s(CDAI23):factor(JIAOYU4)1 | 1.0772 | 1.151 | 4.7547 | 0.0332 |
| s(CDAI23):factor(JIAOYU4)2 | 1.0045 | 1.0089 | 4.3615 | 0.0372 |
| s(CDAI23):factor(JIAOYU4)3 | 1.6185 | 2.0485 | 4.4952 | 0.1171 |

Model statistics

|  |  |
| --- | --- |
| N: | 10904 |
| Adj. r-square: | 0.0539 |
| Deviance explained: | 0.0789 |
| UBRE score (sp.criterion): | -0.361 |
| Scale estimate: | 1 |
| family: | binomial |
| link function: | logit |
