# Supplementary material for: Association between the composite dietary antioxidant index and constipation: Evidence from NHANES 2005–2010
Source: PLoS One. 2024 Sep 27;19(9):e0311168. doi: 10.1371/journal.pone.0311168 (PMC11432863; doi:10.1371/journal.pone.0311168)
Supplement: S1 File — (ZIP) [file pone.0311168.s001.zip › CDAI/all/PROJ2_5_tbl/PROJ2_5_tbl.htm]

|  |
| --- |
| BIANMI24 vs. CDAI23 |

Generalize additive models
Outcome: BIANMI24
Exposure: CDAI23
Linear terms effect

|  |  |  |  |  |  |  |  |
| --- | --- | --- | --- | --- | --- | --- | --- |
|  | Estimate | Std. Error | z value | Pr(>|z|) | exp(est) | 95%CI low | 95%CI upp |
| (Intercept) | -1.3514 | 0.6407 | -2.1093 | 0.0349 | 0.2589 | 0.0738 | 0.9088 |
| factor(HUNYING5)2 | 0.1015 | 0.0861 | 1.1791 | 0.2384 | 1.1069 | 0.935 | 1.3104 |
| factor(HUNYING5)3 | 0.0399 | 0.0965 | 0.4136 | 0.6792 | 1.0407 | 0.8614 | 1.2573 |
| factor(ZHONGZU3)2 | 0.3087 | 0.1314 | 2.3487 | 0.0188 | 1.3617 | 1.0524 | 1.7619 |
| factor(ZHONGZU3)3 | 0.2273 | 0.1049 | 2.1664 | 0.0303 | 1.2552 | 1.0219 | 1.5418 |
| factor(ZHONGZU3)4 | 0.5627 | 0.1126 | 4.9975 | 0 | 1.7553 | 1.4077 | 2.1887 |
| factor(ZHONGZU3)5 | 0.1049 | 0.1939 | 0.5412 | 0.5884 | 1.1106 | 0.7595 | 1.624 |
| PIR6 | -0.1412 | 0.0694 | -2.0356 | 0.0418 | 0.8683 | 0.7579 | 0.9948 |
| factor(BMI7)2 | -0.1781 | 0.0799 | -2.2278 | 0.0259 | 0.8369 | 0.7155 | 0.9788 |
| factor(BMI7)3 | -0.4222 | 0.0828 | -5.1016 | 0 | 0.6556 | 0.5574 | 0.7711 |
| YIYU8 | -0.6272 | 0.0968 | -6.4782 | 0 | 0.5341 | 0.4417 | 0.6457 |
| YUNDONG9 | -0.116 | 0.1004 | -1.1558 | 0.2478 | 0.8905 | 0.7315 | 1.0841 |
| DRINK10 | 0.1101 | 0.0727 | 1.514 | 0.13 | 1.1164 | 0.9681 | 1.2874 |
| factor(XIYAN11)2 | -0.1488 | 0.1056 | -1.4097 | 0.1586 | 0.8617 | 0.7007 | 1.0598 |
| factor(XIYAN11)3 | 0.096 | 0.0864 | 1.1113 | 0.2664 | 1.1008 | 0.9293 | 1.304 |
| GAOXUEYA12 | 0.1838 | 0.0765 | 2.4016 | 0.0163 | 1.2017 | 1.0344 | 1.3962 |
| TANGNIAOBING13 | -0.0082 | 0.1012 | -0.0814 | 0.9351 | 0.9918 | 0.8133 | 1.2094 |
| FEIBING14 | -0.1063 | 0.086 | -1.2356 | 0.2166 | 0.8992 | 0.7596 | 1.0643 |
| XINGZHANGBING15 | -0.3323 | 0.1191 | -2.7902 | 0.0053 | 0.7173 | 0.568 | 0.9059 |
| GANBING16 | 0.2319 | 0.1946 | 1.1921 | 0.2332 | 1.261 | 0.8612 | 1.8465 |
| DANBAIZHI17 | 0.0049 | 0.0026 | 1.8629 | 0.0625 | 1.0049 | 0.9997 | 1.01 |
| TANSHUI18 | 0.0064 | 0.0015 | 4.2642 | 0 | 1.0065 | 1.0035 | 1.0094 |
| XIANWEI19 | -0.0207 | 0.0065 | -3.1952 | 0.0014 | 0.9795 | 0.9671 | 0.992 |
| ZHIFANG20 | 0.006 | 0.0037 | 1.6376 | 0.1015 | 1.006 | 0.9988 | 1.0133 |
| SHUIFEN21 | -1e-04 | 0 | -3.2974 | 0.001 | 0.9999 | 0.9998 | 1 |
| NENGLIANG22 | -0.001 | 4e-04 | -2.7455 | 0.006 | 0.999 | 0.9983 | 0.9997 |
| XINBIE1 | 0.8854 | 0.0804 | 11.0136 | 0 | 2.424 | 2.0706 | 2.8377 |
| AGE2 | -0.0062 | 0.0026 | -2.4299 | 0.0151 | 0.9938 | 0.9888 | 0.9988 |
| factor(JIAOYU4)2 | -0.0527 | 0.0881 | -0.5975 | 0.5502 | 0.9487 | 0.7982 | 1.1276 |
| factor(JIAOYU4)3 | -0.3972 | 0.0857 | -4.6319 | 0 | 0.6722 | 0.5682 | 0.7952 |

Chi-square tests for linear terms

|  |  |  |  |
| --- | --- | --- | --- |
|  | df | Chi.sq | p-value |
| factor(HUNYING5) | 2 | 1.4363 | 0.4877 |
| factor(ZHONGZU3) | 4 | 29.6416 | 0 |
| PIR6 | 1 | 4.1435 | 0.0418 |
| factor(BMI7) | 2 | 26.2072 | 0 |
| YIYU8 | 1 | 41.9669 | 0 |
| YUNDONG9 | 1 | 1.3358 | 0.2478 |
| DRINK10 | 1 | 2.2922 | 0.13 |
| factor(XIYAN11) | 2 | 7.3138 | 0.0258 |
| GAOXUEYA12 | 1 | 5.7676 | 0.0163 |
| TANGNIAOBING13 | 1 | 0.0066 | 0.9351 |
| FEIBING14 | 1 | 1.5268 | 0.2166 |
| XINGZHANGBING15 | 1 | 7.7849 | 0.0053 |
| GANBING16 | 1 | 1.4211 | 0.2332 |
| DANBAIZHI17 | 1 | 3.4703 | 0.0625 |
| TANSHUI18 | 1 | 18.1835 | 0 |
| XIANWEI19 | 1 | 10.2093 | 0.0014 |
| ZHIFANG20 | 1 | 2.6818 | 0.1015 |
| SHUIFEN21 | 1 | 10.8731 | 0.001 |
| NENGLIANG22 | 1 | 7.5378 | 0.006 |
| XINBIE1 | 1 | 121.3002 | 0 |
| AGE2 | 1 | 5.9043 | 0.0151 |
| factor(JIAOYU4) | 2 | 27.5306 | 0 |

Approximate significance of smooth terms

|  |  |  |  |  |
| --- | --- | --- | --- | --- |
|  | edf | Ref.df | Chi.sq | p-value |
| s(CDAI23):factor(HUNYING5)1 | 1.0027 | 1.0054 | 10.7231 | 0.0011 |
| s(CDAI23):factor(HUNYING5)2 | 1.0021 | 1.0041 | 0.5621 | 0.4552 |
| s(CDAI23):factor(HUNYING5)3 | 2.7285 | 3.4747 | 4.9804 | 0.2565 |

Model statistics

|  |  |
| --- | --- |
| N: | 10904 |
| Adj. r-square: | 0.0545 |
| Deviance explained: | 0.0796 |
| UBRE score (sp.criterion): | -0.3612 |
| Scale estimate: | 1 |
| family: | binomial |
| link function: | logit |
