# Supplementary material for: Association between the composite dietary antioxidant index and constipation: Evidence from NHANES 2005–2010
Source: PLoS One. 2024 Sep 27;19(9):e0311168. doi: 10.1371/journal.pone.0311168 (PMC11432863; doi:10.1371/journal.pone.0311168)
Supplement: S1 File — (ZIP) [file pone.0311168.s001.zip › CDAI/all/PROJ2_6_tbl/PROJ2_6_tbl.htm]

|  |
| --- |
| BIANMI24 vs. CDAI23 |

Generalize additive models
Outcome: BIANMI24
Exposure: CDAI23
Linear terms effect

|  |  |  |  |  |  |  |  |
| --- | --- | --- | --- | --- | --- | --- | --- |
|  | Estimate | Std. Error | z value | Pr(>|z|) | exp(est) | 95%CI low | 95%CI upp |
| (Intercept) | -1.4427 | 0.6384 | -2.2598 | 0.0238 | 0.2363 | 0.0676 | 0.8258 |
| factor(PIR6)2 | -0.1597 | 0.072 | -2.2175 | 0.0266 | 0.8524 | 0.7401 | 0.9816 |
| factor(ZHONGZU3)2 | 0.3102 | 0.1314 | 2.3609 | 0.0182 | 1.3636 | 1.0541 | 1.7641 |
| factor(ZHONGZU3)3 | 0.2297 | 0.1049 | 2.19 | 0.0285 | 1.2582 | 1.0244 | 1.5454 |
| factor(ZHONGZU3)4 | 0.5618 | 0.1126 | 4.9913 | 0 | 1.7539 | 1.4066 | 2.1868 |
| factor(ZHONGZU3)5 | 0.1036 | 0.1938 | 0.5342 | 0.5932 | 1.1091 | 0.7585 | 1.6217 |
| factor(BMI7)2 | -0.1791 | 0.0799 | -2.2414 | 0.025 | 0.836 | 0.7148 | 0.9778 |
| factor(BMI7)3 | -0.4187 | 0.0827 | -5.0615 | 0 | 0.6579 | 0.5594 | 0.7737 |
| YIYU8 | -0.6284 | 0.0968 | -6.491 | 0 | 0.5335 | 0.4413 | 0.6449 |
| YUNDONG9 | -0.1201 | 0.1003 | -1.1977 | 0.231 | 0.8869 | 0.7286 | 1.0794 |
| DRINK10 | 0.1086 | 0.0727 | 1.4931 | 0.1354 | 1.1147 | 0.9666 | 1.2854 |
| factor(XIYAN11)2 | -0.1433 | 0.1055 | -1.3579 | 0.1745 | 0.8665 | 0.7046 | 1.0656 |
| factor(XIYAN11)3 | 0.0959 | 0.0864 | 1.1097 | 0.2671 | 1.1006 | 0.9291 | 1.3038 |
| GAOXUEYA12 | 0.1855 | 0.0765 | 2.4234 | 0.0154 | 1.2038 | 1.0361 | 1.3987 |
| TANGNIAOBING13 | -0.0123 | 0.1012 | -0.1213 | 0.9034 | 0.9878 | 0.81 | 1.2046 |
| FEIBING14 | -0.1056 | 0.086 | -1.228 | 0.2194 | 0.8998 | 0.7603 | 1.065 |
| XINGZHANGBING15 | -0.3287 | 0.1191 | -2.7594 | 0.0058 | 0.7199 | 0.57 | 0.9092 |
| GANBING16 | 0.2307 | 0.1945 | 1.1862 | 0.2355 | 1.2595 | 0.8603 | 1.8439 |
| DANBAIZHI17 | 0.0045 | 0.0026 | 1.7298 | 0.0837 | 1.0045 | 0.9994 | 1.0097 |
| TANSHUI18 | 0.0064 | 0.0015 | 4.2117 | 0 | 1.0064 | 1.0034 | 1.0094 |
| XIANWEI19 | -0.0208 | 0.0065 | -3.2085 | 0.0013 | 0.9794 | 0.967 | 0.9919 |
| ZHIFANG20 | 0.0059 | 0.0037 | 1.6072 | 0.108 | 1.0059 | 0.9987 | 1.0132 |
| SHUIFEN21 | -1e-04 | 0 | -3.3227 | 9e-04 | 0.9999 | 0.9998 | 1 |
| NENGLIANG22 | -0.001 | 4e-04 | -2.6983 | 0.007 | 0.999 | 0.9983 | 0.9997 |
| XINBIE1 | 0.8897 | 0.0804 | 11.0648 | 0 | 2.4344 | 2.0795 | 2.85 |
| AGE2 | -0.0062 | 0.0026 | -2.418 | 0.0156 | 0.9938 | 0.9888 | 0.9988 |
| factor(JIAOYU4)2 | -0.0527 | 0.0881 | -0.5981 | 0.5498 | 0.9487 | 0.7983 | 1.1274 |
| factor(JIAOYU4)3 | -0.3967 | 0.0857 | -4.6295 | 0 | 0.6725 | 0.5685 | 0.7955 |
| factor(HUNYING5)2 | 0.0531 | 0.0823 | 0.6458 | 0.5184 | 1.0546 | 0.8975 | 1.2392 |
| factor(HUNYING5)3 | 0.0232 | 0.0933 | 0.2486 | 0.8037 | 1.0235 | 0.8524 | 1.2289 |

Chi-square tests for linear terms

|  |  |  |  |
| --- | --- | --- | --- |
|  | df | Chi.sq | p-value |
| factor(PIR6) | 1 | 4.9175 | 0.0266 |
| factor(ZHONGZU3) | 4 | 29.4527 | 0 |
| factor(BMI7) | 2 | 25.7682 | 0 |
| YIYU8 | 1 | 42.1326 | 0 |
| YUNDONG9 | 1 | 1.4344 | 0.231 |
| DRINK10 | 1 | 2.2294 | 0.1354 |
| factor(XIYAN11) | 2 | 7.0101 | 0.03 |
| GAOXUEYA12 | 1 | 5.8728 | 0.0154 |
| TANGNIAOBING13 | 1 | 0.0147 | 0.9034 |
| FEIBING14 | 1 | 1.508 | 0.2194 |
| XINGZHANGBING15 | 1 | 7.6142 | 0.0058 |
| GANBING16 | 1 | 1.4071 | 0.2355 |
| DANBAIZHI17 | 1 | 2.9921 | 0.0837 |
| TANSHUI18 | 1 | 17.7385 | 0 |
| XIANWEI19 | 1 | 10.2947 | 0.0013 |
| ZHIFANG20 | 1 | 2.583 | 0.108 |
| SHUIFEN21 | 1 | 11.0402 | 9e-04 |
| NENGLIANG22 | 1 | 7.2806 | 0.007 |
| XINBIE1 | 1 | 122.4302 | 0 |
| AGE2 | 1 | 5.8468 | 0.0156 |
| factor(JIAOYU4) | 2 | 27.4734 | 0 |
| factor(HUNYING5) | 2 | 0.4374 | 0.8036 |

Approximate significance of smooth terms

|  |  |  |  |  |
| --- | --- | --- | --- | --- |
|  | edf | Ref.df | Chi.sq | p-value |
| s(CDAI23):factor(PIR6)1 | 1.0055 | 1.0109 | 3.1632 | 0.0763 |
| s(CDAI23):factor(PIR6)2 | 1.0023 | 1.0046 | 8.3666 | 0.0039 |

Model statistics

|  |  |
| --- | --- |
| N: | 10904 |
| Adj. r-square: | 0.054 |
| Deviance explained: | 0.0787 |
| UBRE score (sp.criterion): | -0.3611 |
| Scale estimate: | 1 |
| family: | binomial |
| link function: | logit |
