# Supplementary material for: Association between the composite dietary antioxidant index and constipation: Evidence from NHANES 2005–2010
Source: PLoS One. 2024 Sep 27;19(9):e0311168. doi: 10.1371/journal.pone.0311168 (PMC11432863; doi:10.1371/journal.pone.0311168)
Supplement: S1 File — (ZIP) [file pone.0311168.s001.zip › CDAI/all/PROJ2_7_tbl/PROJ2_7_tbl.htm]

|  |
| --- |
| BIANMI24 vs. CDAI23 |

Generalize additive models
Outcome: BIANMI24
Exposure: CDAI23
Linear terms effect

|  |  |  |  |  |  |  |  |
| --- | --- | --- | --- | --- | --- | --- | --- |
|  | Estimate | Std. Error | z value | Pr(>|z|) | exp(est) | 95%CI low | 95%CI upp |
| (Intercept) | -1.3274 | 0.6413 | -2.0697 | 0.0385 | 0.2652 | 0.0754 | 0.932 |
| factor(BMI7)2 | -0.1334 | 0.0832 | -1.6031 | 0.1089 | 0.8751 | 0.7434 | 1.0302 |
| factor(BMI7)3 | -0.3743 | 0.0864 | -4.331 | 0 | 0.6878 | 0.5806 | 0.8147 |
| factor(ZHONGZU3)2 | 0.3137 | 0.1315 | 2.3854 | 0.0171 | 1.3685 | 1.0575 | 1.7708 |
| factor(ZHONGZU3)3 | 0.2262 | 0.105 | 2.1544 | 0.0312 | 1.2538 | 1.0206 | 1.5402 |
| factor(ZHONGZU3)4 | 0.5608 | 0.1127 | 4.9752 | 0 | 1.752 | 1.4047 | 2.1852 |
| factor(ZHONGZU3)5 | 0.1034 | 0.1941 | 0.5328 | 0.5942 | 1.109 | 0.758 | 1.6224 |
| YIYU8 | -0.6255 | 0.097 | -6.4454 | 0 | 0.535 | 0.4423 | 0.6471 |
| YUNDONG9 | -0.129 | 0.1004 | -1.2854 | 0.1986 | 0.8789 | 0.722 | 1.0701 |
| DRINK10 | 0.1101 | 0.0728 | 1.5114 | 0.1307 | 1.1163 | 0.9679 | 1.2876 |
| factor(XIYAN11)2 | -0.1553 | 0.1059 | -1.4668 | 0.1424 | 0.8562 | 0.6958 | 1.0536 |
| factor(XIYAN11)3 | 0.099 | 0.0865 | 1.1449 | 0.2523 | 1.1041 | 0.9319 | 1.308 |
| GAOXUEYA12 | 0.1919 | 0.0766 | 2.5067 | 0.0122 | 1.2116 | 1.0427 | 1.4078 |
| TANGNIAOBING13 | -0.0171 | 0.1012 | -0.1692 | 0.8656 | 0.983 | 0.8061 | 1.1987 |
| FEIBING14 | -0.1102 | 0.0861 | -1.2807 | 0.2003 | 0.8956 | 0.7566 | 1.0602 |
| XINGZHANGBING15 | -0.33 | 0.1192 | -2.7676 | 0.0056 | 0.7189 | 0.5691 | 0.9082 |
| GANBING16 | 0.2248 | 0.1945 | 1.1559 | 0.2477 | 1.2521 | 0.8552 | 1.833 |
| DANBAIZHI17 | 0.0045 | 0.0026 | 1.7431 | 0.0813 | 1.0045 | 0.9994 | 1.0097 |
| TANSHUI18 | 0.0064 | 0.0015 | 4.2382 | 0 | 1.0064 | 1.0034 | 1.0094 |
| XIANWEI19 | -0.0206 | 0.0065 | -3.1771 | 0.0015 | 0.9796 | 0.9672 | 0.9921 |
| ZHIFANG20 | 0.006 | 0.0037 | 1.6305 | 0.103 | 1.006 | 0.9988 | 1.0133 |
| SHUIFEN21 | -1e-04 | 0 | -3.3371 | 8e-04 | 0.9999 | 0.9998 | 0.9999 |
| NENGLIANG22 | -0.001 | 4e-04 | -2.7204 | 0.0065 | 0.999 | 0.9983 | 0.9997 |
| XINBIE1 | 0.8971 | 0.0806 | 11.1351 | 0 | 2.4525 | 2.0942 | 2.872 |
| AGE2 | -0.0062 | 0.0026 | -2.3919 | 0.0168 | 0.9939 | 0.9889 | 0.9989 |
| factor(JIAOYU4)2 | -0.0627 | 0.0884 | -0.7098 | 0.4778 | 0.9392 | 0.7899 | 1.1168 |
| factor(JIAOYU4)3 | -0.4041 | 0.0858 | -4.7068 | 0 | 0.6676 | 0.5642 | 0.7899 |
| factor(HUNYING5)2 | 0.0523 | 0.0824 | 0.6343 | 0.5259 | 1.0537 | 0.8965 | 1.2384 |
| factor(HUNYING5)3 | 0.0299 | 0.0934 | 0.3202 | 0.7488 | 1.0304 | 0.858 | 1.2374 |
| PIR6 | -0.1403 | 0.0695 | -2.0202 | 0.0434 | 0.8691 | 0.7584 | 0.9958 |

Chi-square tests for linear terms

|  |  |  |  |
| --- | --- | --- | --- |
|  | df | Chi.sq | p-value |
| factor(BMI7) | 2 | 19.2817 | 1e-04 |
| factor(ZHONGZU3) | 4 | 29.4563 | 0 |
| YIYU8 | 1 | 41.5437 | 0 |
| YUNDONG9 | 1 | 1.6524 | 0.1986 |
| DRINK10 | 1 | 2.2843 | 0.1307 |
| factor(XIYAN11) | 2 | 7.8174 | 0.0201 |
| GAOXUEYA12 | 1 | 6.2833 | 0.0122 |
| TANGNIAOBING13 | 1 | 0.0286 | 0.8656 |
| FEIBING14 | 1 | 1.6402 | 0.2003 |
| XINGZHANGBING15 | 1 | 7.6598 | 0.0056 |
| GANBING16 | 1 | 1.3362 | 0.2477 |
| DANBAIZHI17 | 1 | 3.0386 | 0.0813 |
| TANSHUI18 | 1 | 17.9623 | 0 |
| XIANWEI19 | 1 | 10.0941 | 0.0015 |
| ZHIFANG20 | 1 | 2.6587 | 0.103 |
| SHUIFEN21 | 1 | 11.1363 | 8e-04 |
| NENGLIANG22 | 1 | 7.4004 | 0.0065 |
| XINBIE1 | 1 | 123.9901 | 0 |
| AGE2 | 1 | 5.7211 | 0.0168 |
| factor(JIAOYU4) | 2 | 27.8135 | 0 |
| factor(HUNYING5) | 2 | 0.4494 | 0.7988 |
| PIR6 | 1 | 4.0812 | 0.0434 |

Approximate significance of smooth terms

|  |  |  |  |  |
| --- | --- | --- | --- | --- |
|  | edf | Ref.df | Chi.sq | p-value |
| s(CDAI23):factor(BMI7)1 | 4.5019 | 5.5904 | 16.4522 | 0.0093 |
| s(CDAI23):factor(BMI7)2 | 1.0005 | 1.0011 | 1.6594 | 0.198 |
| s(CDAI23):factor(BMI7)3 | 1.0044 | 1.0089 | 1.8502 | 0.1751 |

Model statistics

|  |  |
| --- | --- |
| N: | 10904 |
| Adj. r-square: | 0.055 |
| Deviance explained: | 0.0802 |
| UBRE score (sp.criterion): | -0.3613 |
| Scale estimate: | 1 |
| family: | binomial |
| link function: | logit |
