# Supplementary figures and images for: Association between the composite dietary antioxidant index and constipation: Evidence from NHANES 2005–2010 (part 2 of 2)
Source: PLoS One. 2024 Sep 27;19(9):e0311168. doi: 10.1371/journal.pone.0311168 (PMC11432863; doi:10.1371/journal.pone.0311168)

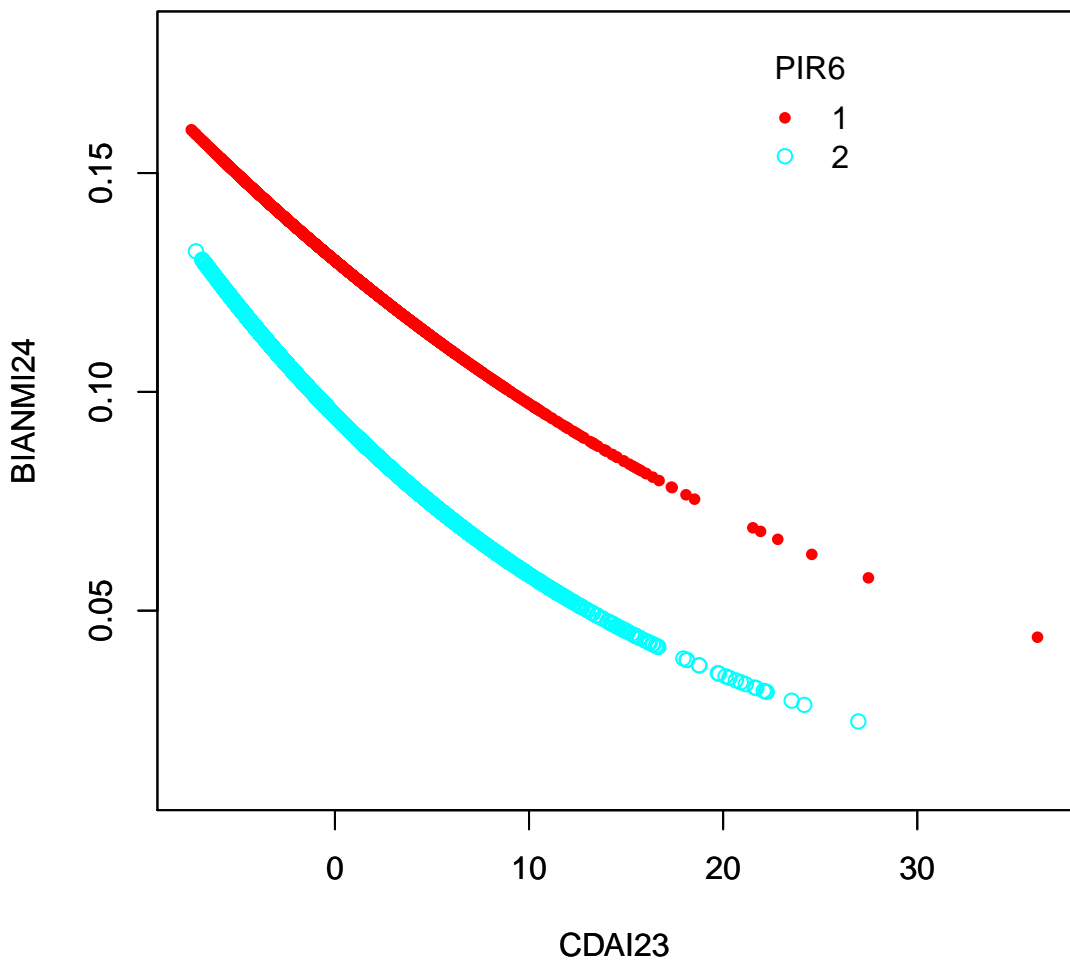

Supplement: S1 File — (ZIP) [file pone.0311168.s001.zip › CDAI/all/PROJ2_6_tbl/PROJ2_6_tbl_BIANMI24_CDAI23_smooth.pdf]

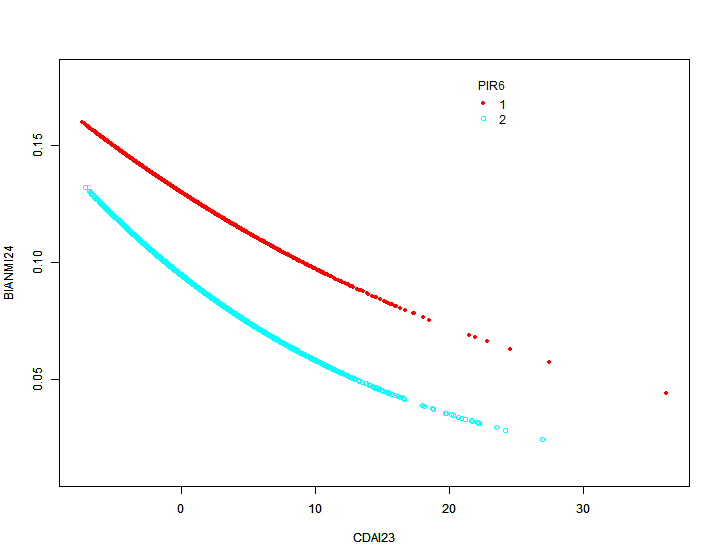

Supplement: S1 File — (ZIP) [file pone.0311168.s001.zip › CDAI/all/PROJ2_6_tbl/PROJ2_6_tbl_BIANMI24_CDAI23_smooth.png]

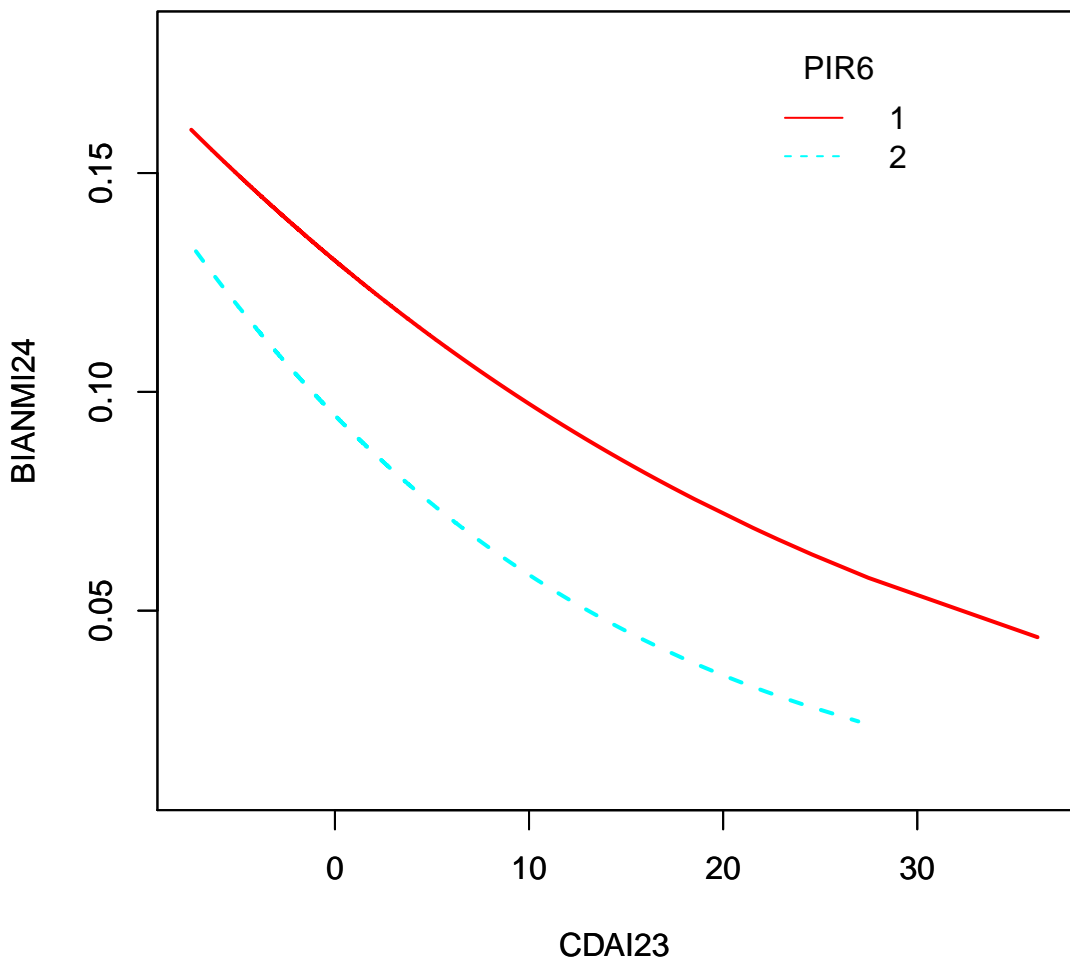

Supplement: S1 File — (ZIP) [file pone.0311168.s001.zip › CDAI/all/PROJ2_6_tbl/PROJ2_6_tbl_BIANMI24_CDAI23_smooth1.pdf]

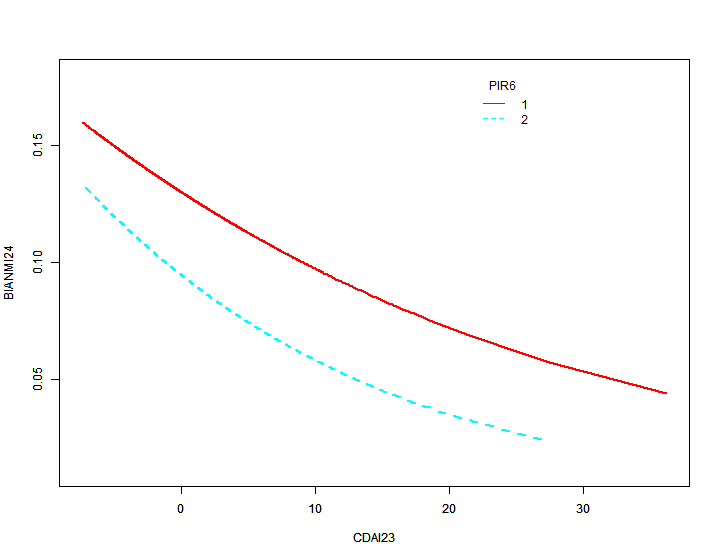

Supplement: S1 File — (ZIP) [file pone.0311168.s001.zip › CDAI/all/PROJ2_6_tbl/PROJ2_6_tbl_BIANMI24_CDAI23_smooth1.png]

BIANMI24

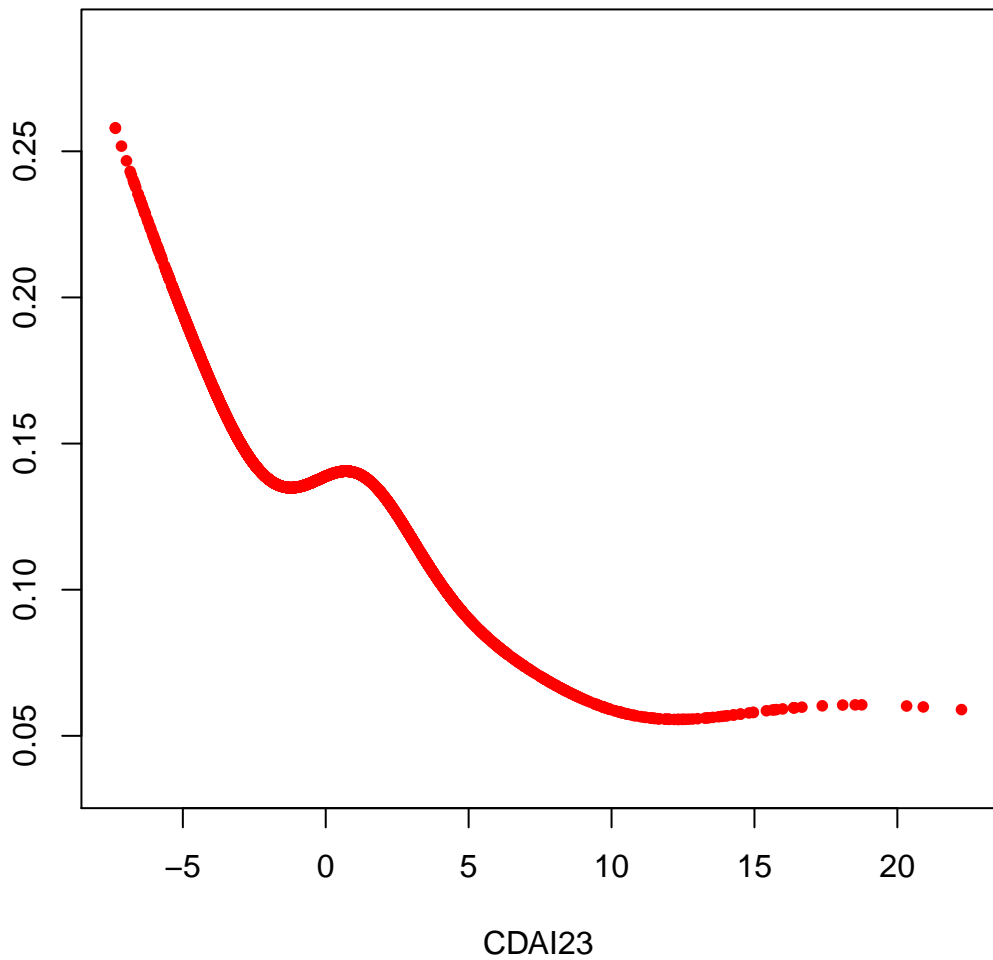

Supplement: S1 File — (ZIP) [file pone.0311168.s001.zip › CDAI/all/PROJ2_7_tbl/PROJ2_7_tbl_BIANMI24_CDAI23_BMI7_1_smooth.pdf]

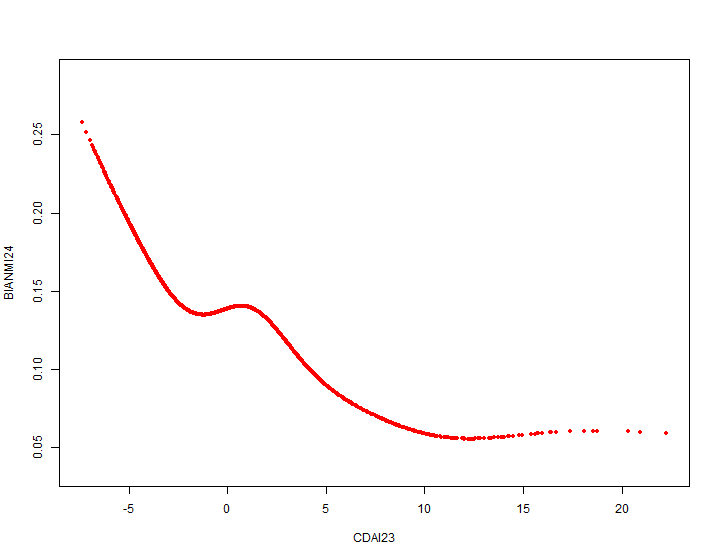

Supplement: S1 File — (ZIP) [file pone.0311168.s001.zip › CDAI/all/PROJ2_7_tbl/PROJ2_7_tbl_BIANMI24_CDAI23_BMI7_1_smooth.png]

BIANMI24

0.12

0.10

0.08

0.06

-5

0

5

10

15

20

25

CDAI23

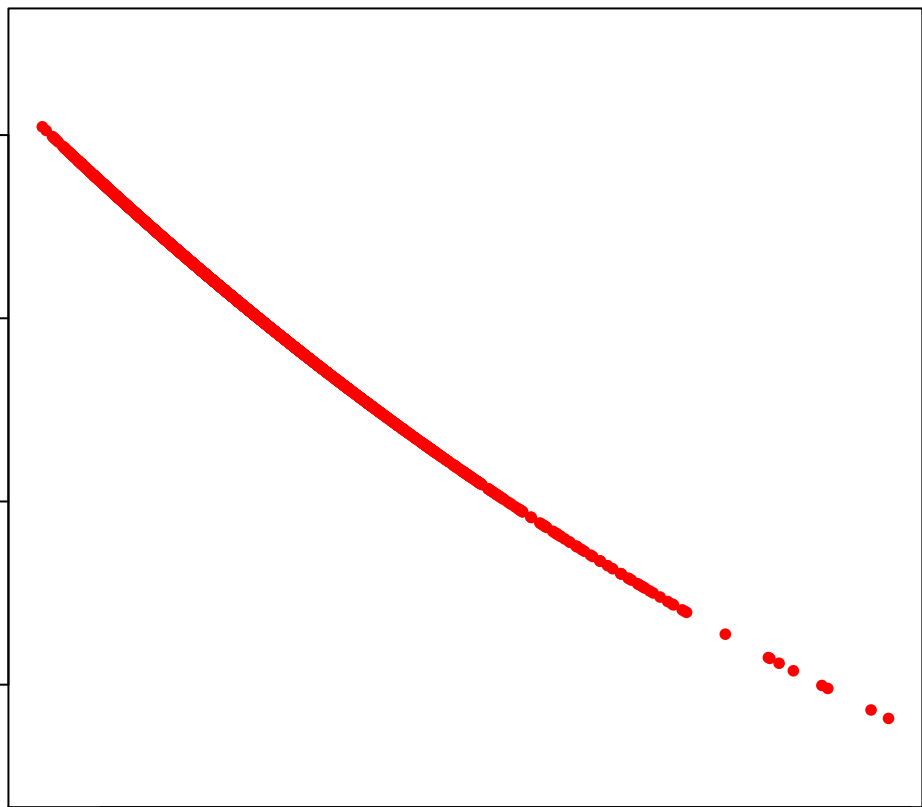

Supplement: S1 File — (ZIP) [file pone.0311168.s001.zip › CDAI/all/PROJ2_7_tbl/PROJ2_7_tbl_BIANMI24_CDAI23_BMI7_2_smooth.pdf]

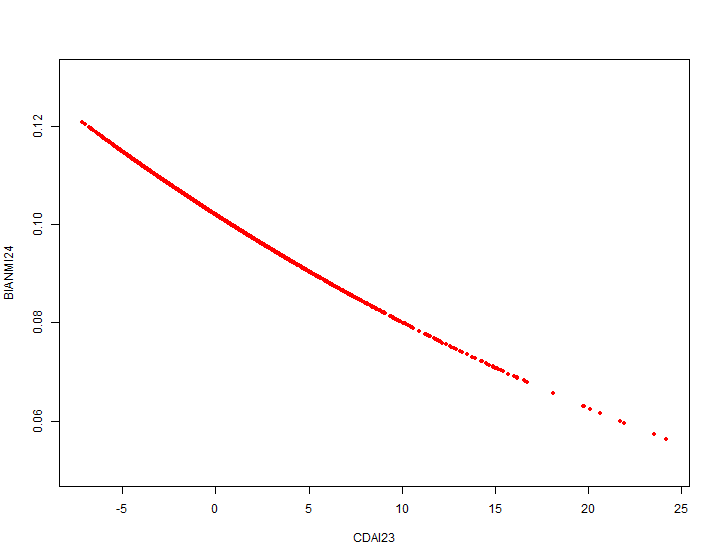

Supplement: S1 File — (ZIP) [file pone.0311168.s001.zip › CDAI/all/PROJ2_7_tbl/PROJ2_7_tbl_BIANMI24_CDAI23_BMI7_2_smooth.png]

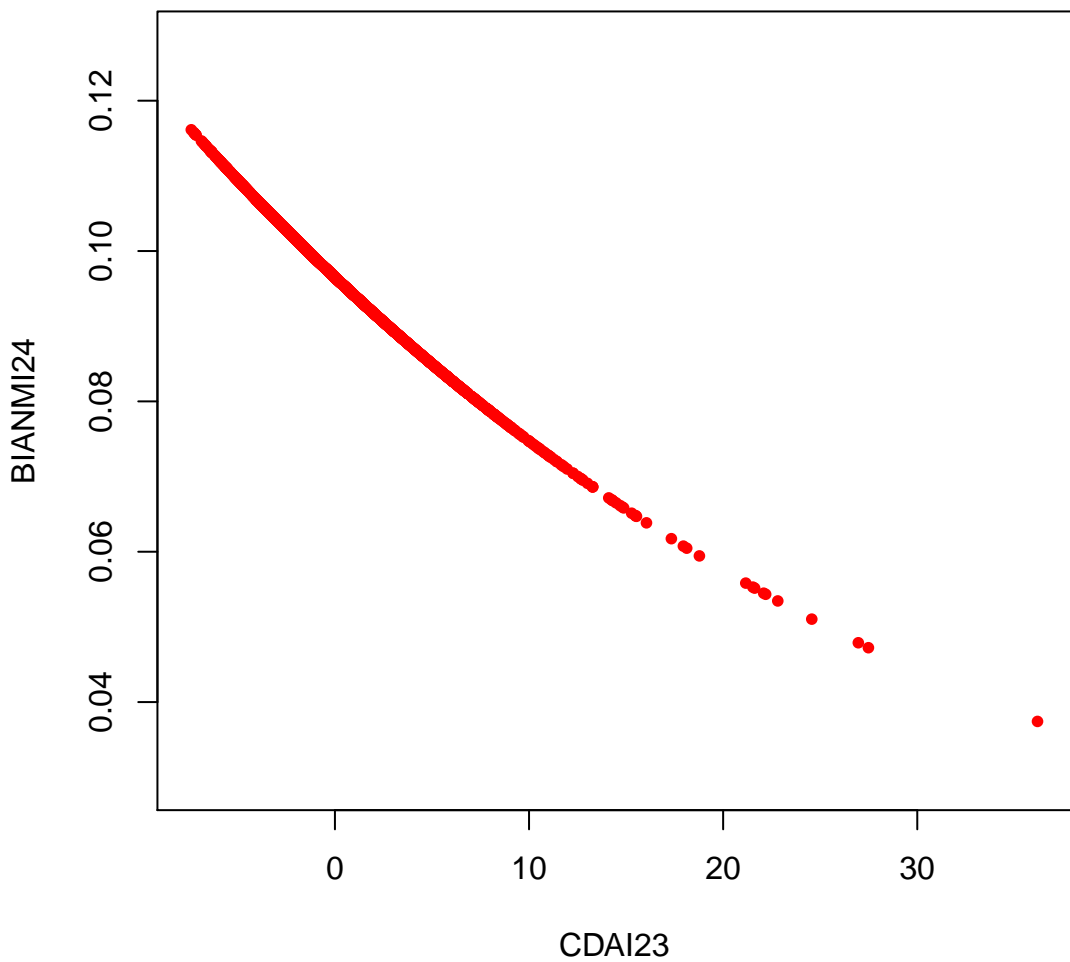

Supplement: S1 File — (ZIP) [file pone.0311168.s001.zip › CDAI/all/PROJ2_7_tbl/PROJ2_7_tbl_BIANMI24_CDAI23_BMI7_3_smooth.pdf]

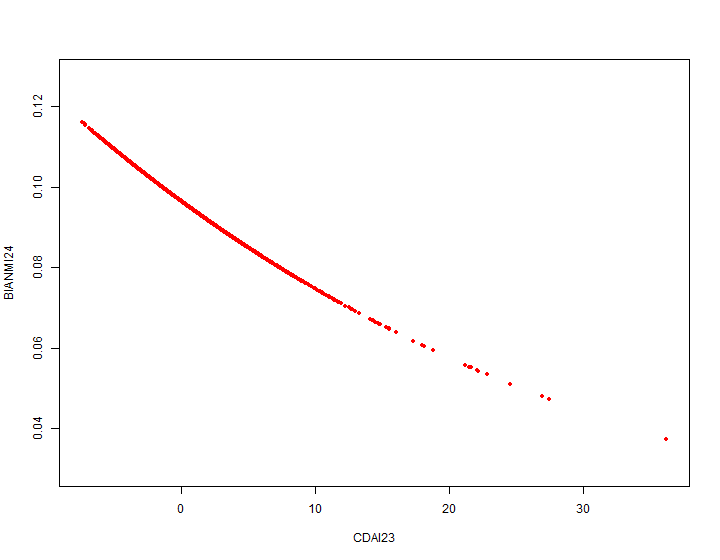

Supplement: S1 File — (ZIP) [file pone.0311168.s001.zip › CDAI/all/PROJ2_7_tbl/PROJ2_7_tbl_BIANMI24_CDAI23_BMI7_3_smooth.png]
