# Supplementary figures and images for: Association between the composite dietary antioxidant index and constipation: Evidence from NHANES 2005–2010 (part 1 of 2)
Source: PLoS One. 2024 Sep 27;19(9):e0311168. doi: 10.1371/journal.pone.0311168 (PMC11432863; doi:10.1371/journal.pone.0311168)

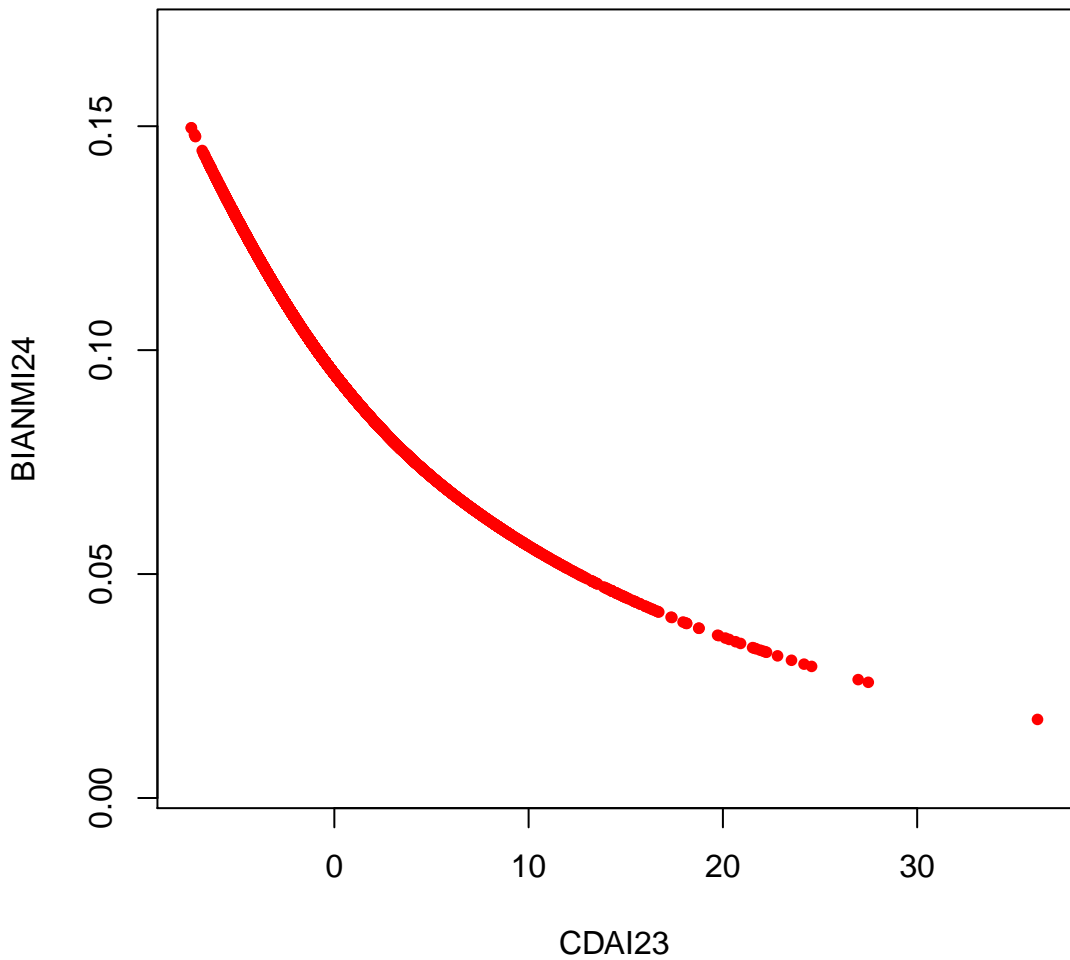

Supplement: S1 File — (ZIP) [file pone.0311168.s001.zip › CDAI/all/PROJ2_10_tbl/PROJ2_10_tbl_BIANMI24_CDAI23_DRINK10_1_smooth.pdf]

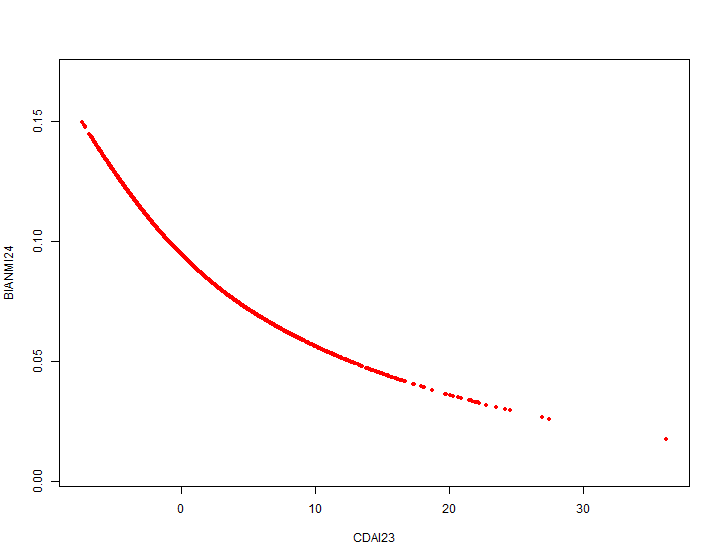

Supplement: S1 File — (ZIP) [file pone.0311168.s001.zip › CDAI/all/PROJ2_10_tbl/PROJ2_10_tbl_BIANMI24_CDAI23_DRINK10_1_smooth.png]

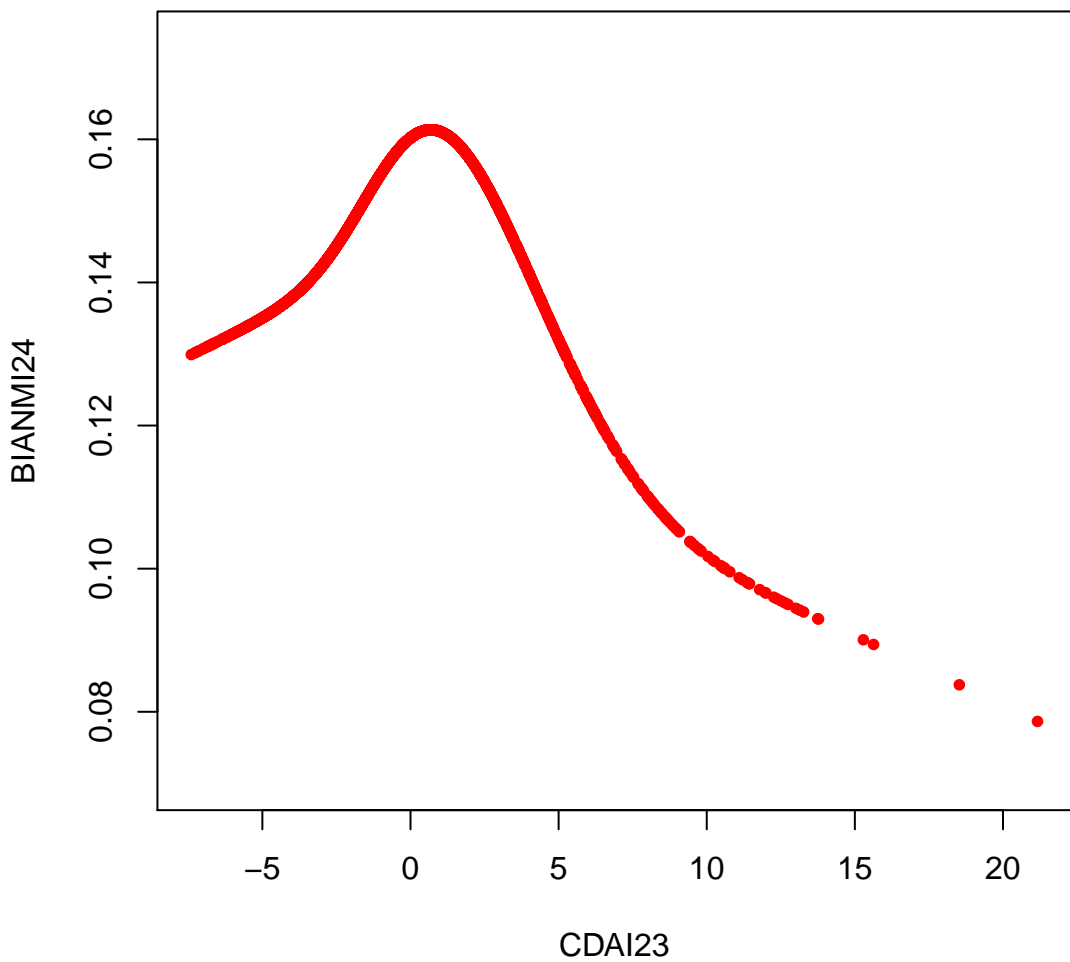

Supplement: S1 File — (ZIP) [file pone.0311168.s001.zip › CDAI/all/PROJ2_10_tbl/PROJ2_10_tbl_BIANMI24_CDAI23_DRINK10_2_smooth.pdf]

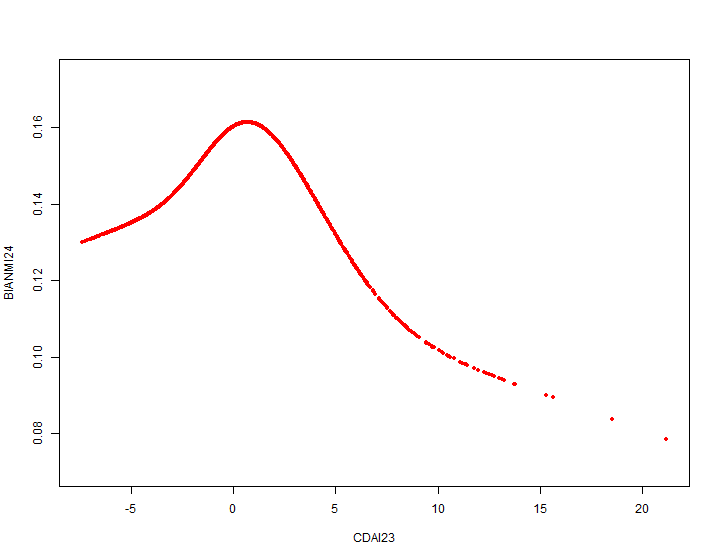

Supplement: S1 File — (ZIP) [file pone.0311168.s001.zip › CDAI/all/PROJ2_10_tbl/PROJ2_10_tbl_BIANMI24_CDAI23_DRINK10_2_smooth.png]

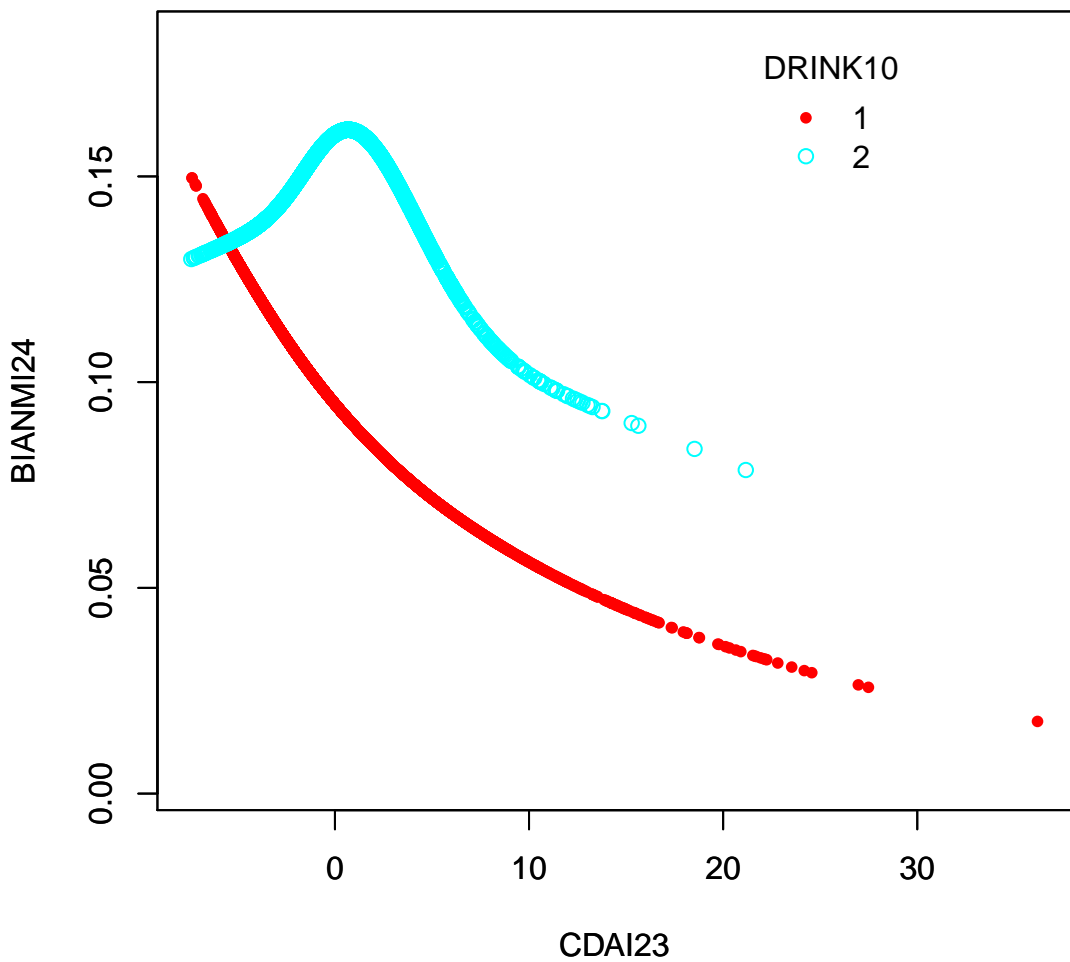

Supplement: S1 File — (ZIP) [file pone.0311168.s001.zip › CDAI/all/PROJ2_10_tbl/PROJ2_10_tbl_BIANMI24_CDAI23_smooth.pdf]

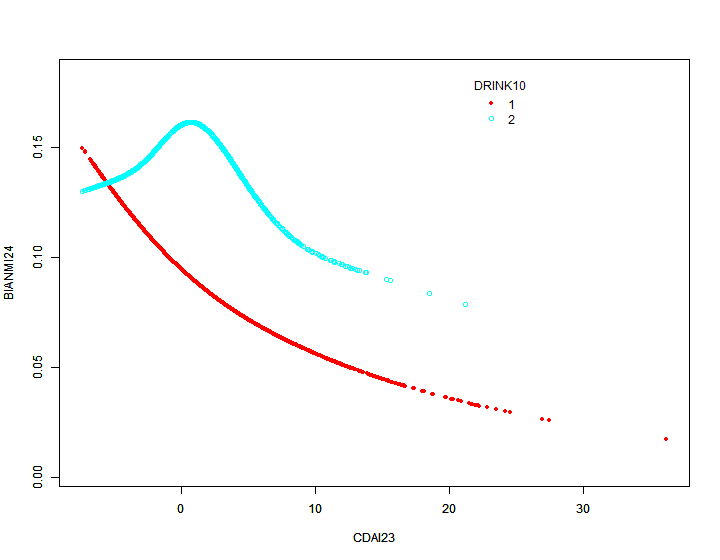

Supplement: S1 File — (ZIP) [file pone.0311168.s001.zip › CDAI/all/PROJ2_10_tbl/PROJ2_10_tbl_BIANMI24_CDAI23_smooth.png]

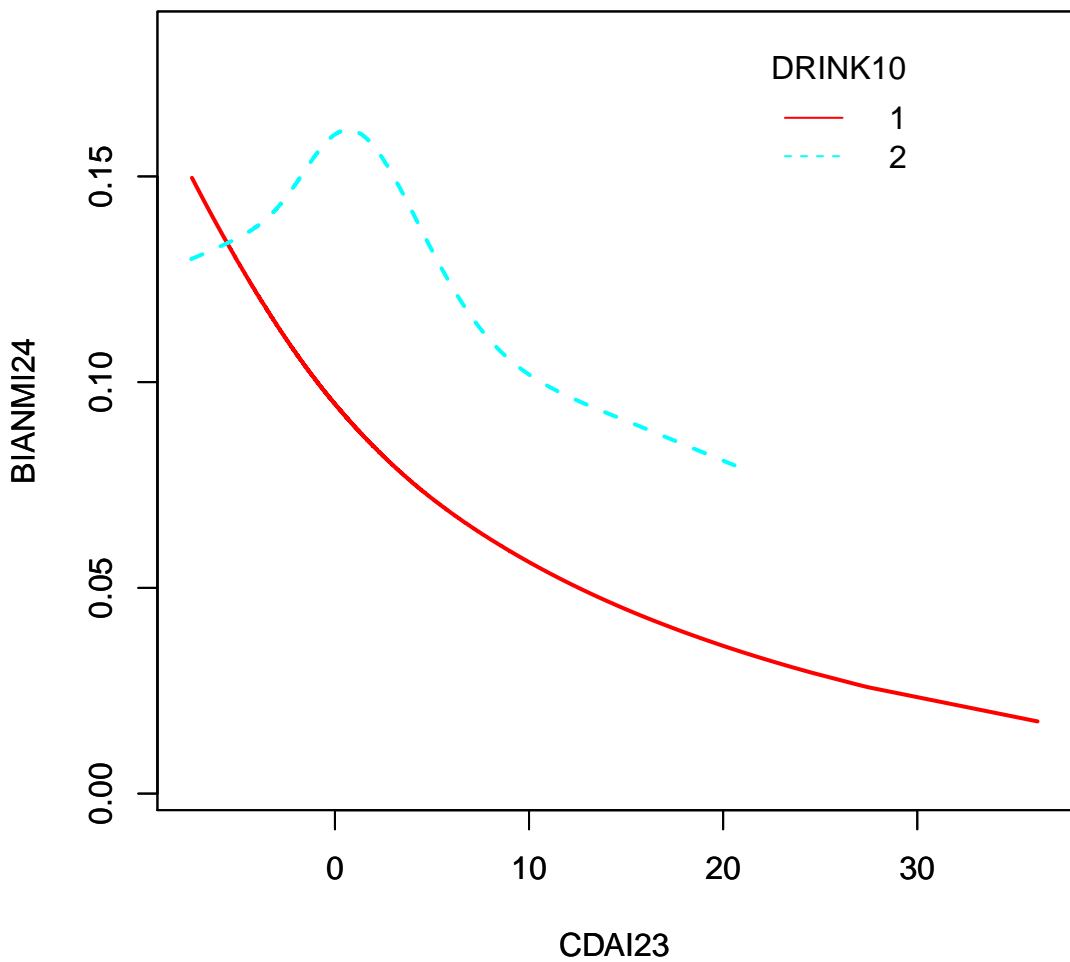

Supplement: S1 File — (ZIP) [file pone.0311168.s001.zip › CDAI/all/PROJ2_10_tbl/PROJ2_10_tbl_BIANMI24_CDAI23_smooth1.pdf]

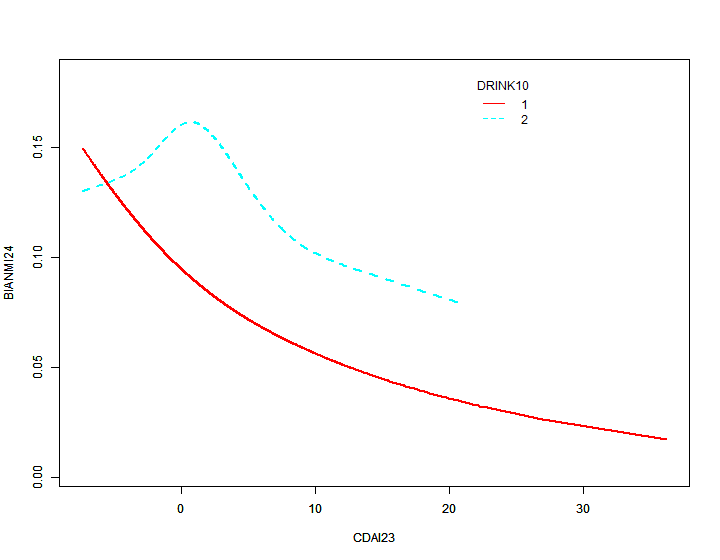

Supplement: S1 File — (ZIP) [file pone.0311168.s001.zip › CDAI/all/PROJ2_10_tbl/PROJ2_10_tbl_BIANMI24_CDAI23_smooth1.png]

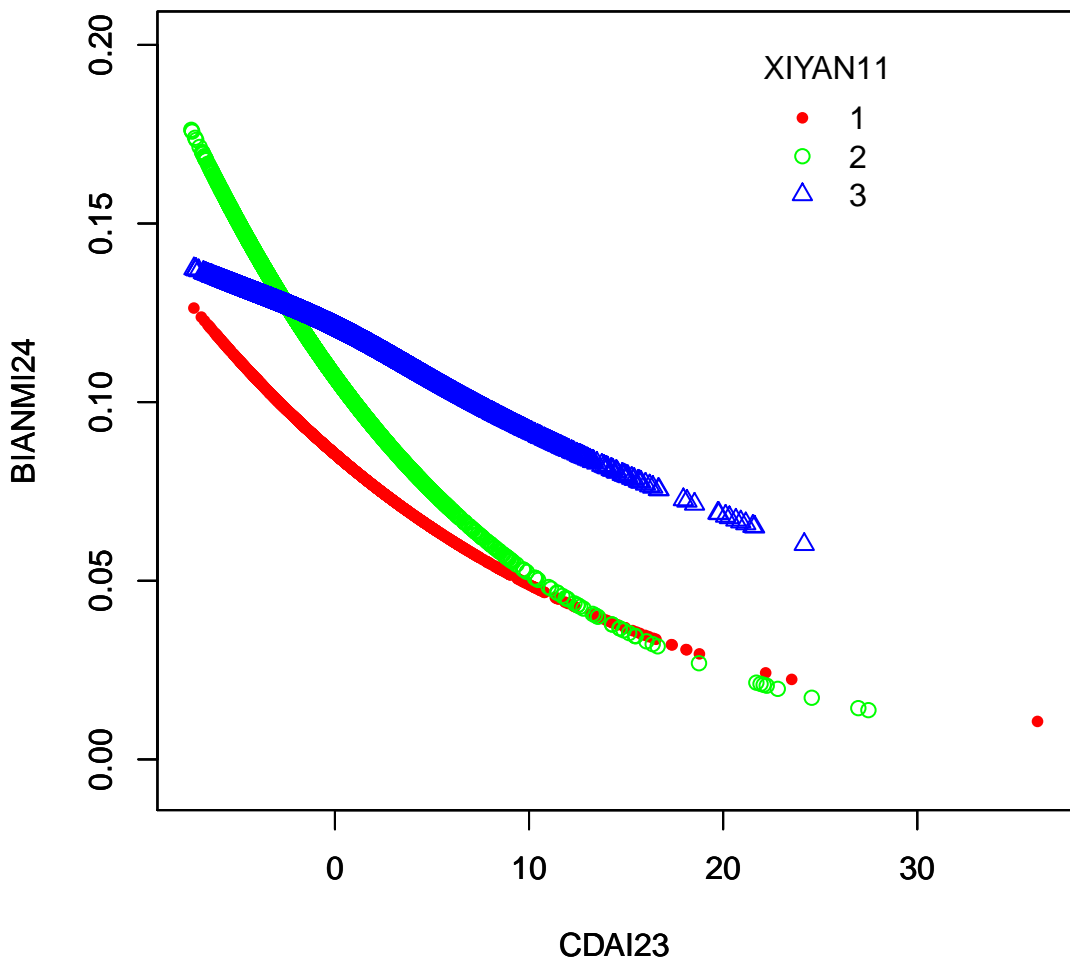

Supplement: S1 File — (ZIP) [file pone.0311168.s001.zip › CDAI/all/PROJ2_11_tbl/PROJ2_11_tbl_BIANMI24_CDAI23_smooth.pdf]

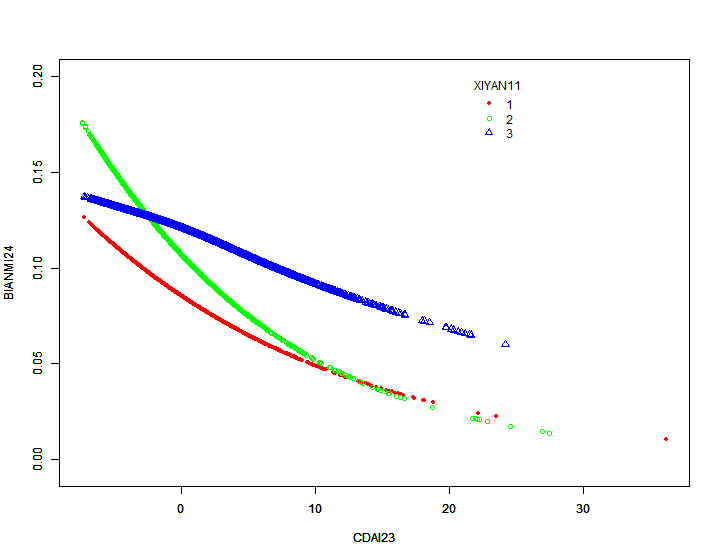

Supplement: S1 File — (ZIP) [file pone.0311168.s001.zip › CDAI/all/PROJ2_11_tbl/PROJ2_11_tbl_BIANMI24_CDAI23_smooth.png]

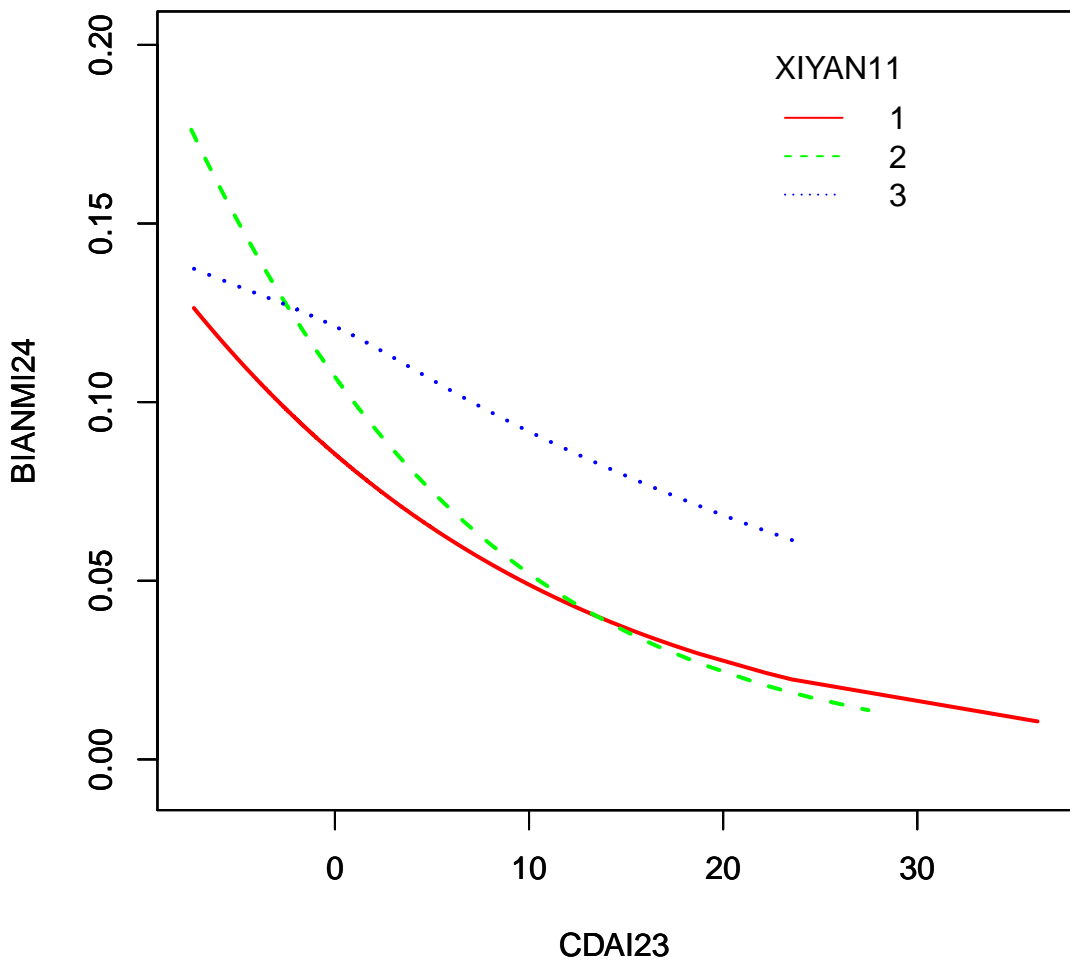

Supplement: S1 File — (ZIP) [file pone.0311168.s001.zip › CDAI/all/PROJ2_11_tbl/PROJ2_11_tbl_BIANMI24_CDAI23_smooth1.pdf]

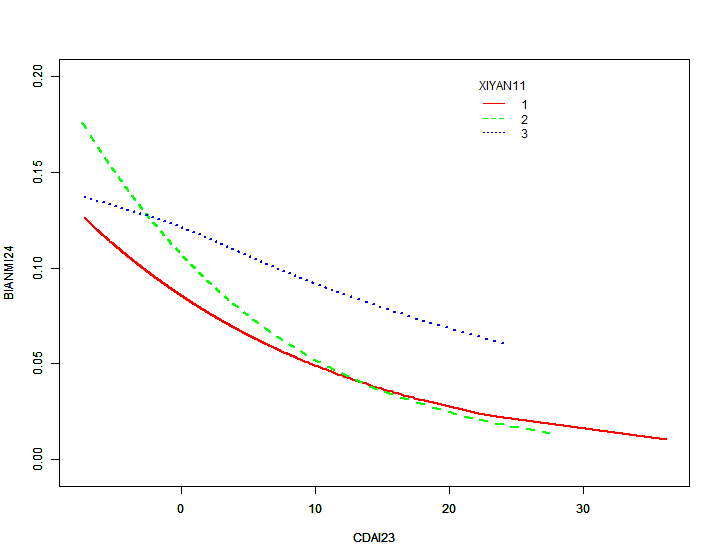

Supplement: S1 File — (ZIP) [file pone.0311168.s001.zip › CDAI/all/PROJ2_11_tbl/PROJ2_11_tbl_BIANMI24_CDAI23_smooth1.png]

BIANMI24

0.10

0.05

0.00

0

10

20

30

CDAI23

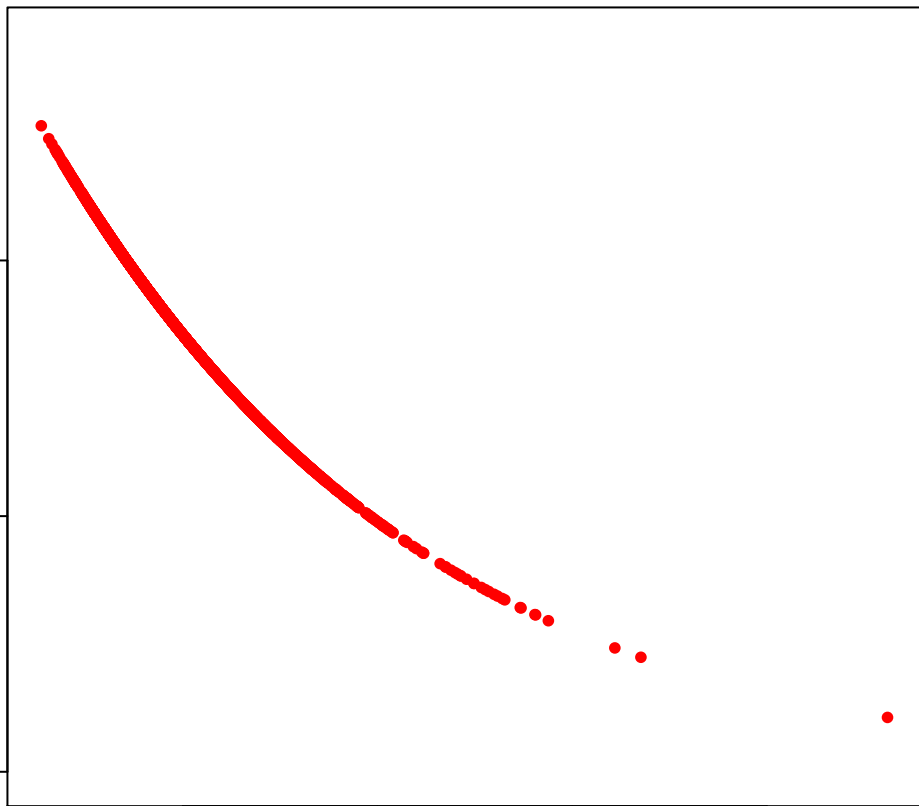

Supplement: S1 File — (ZIP) [file pone.0311168.s001.zip › CDAI/all/PROJ2_11_tbl/PROJ2_11_tbl_BIANMI24_CDAI23_XIYAN11_1_smooth.pdf]

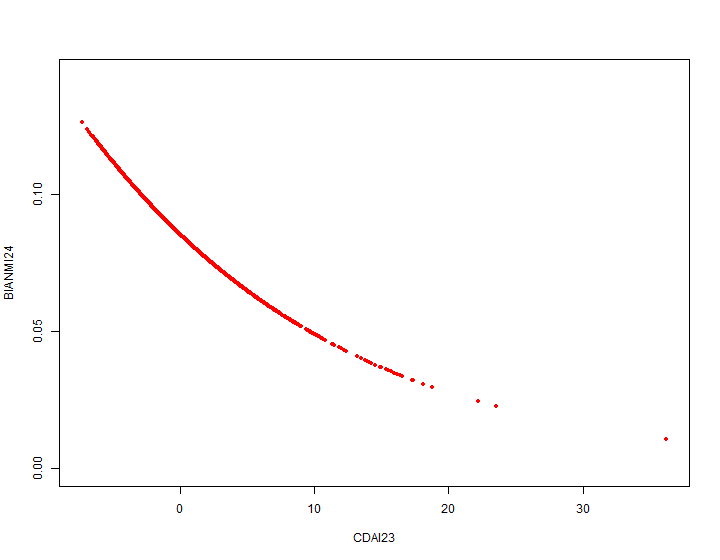

Supplement: S1 File — (ZIP) [file pone.0311168.s001.zip › CDAI/all/PROJ2_11_tbl/PROJ2_11_tbl_BIANMI24_CDAI23_XIYAN11_1_smooth.png]

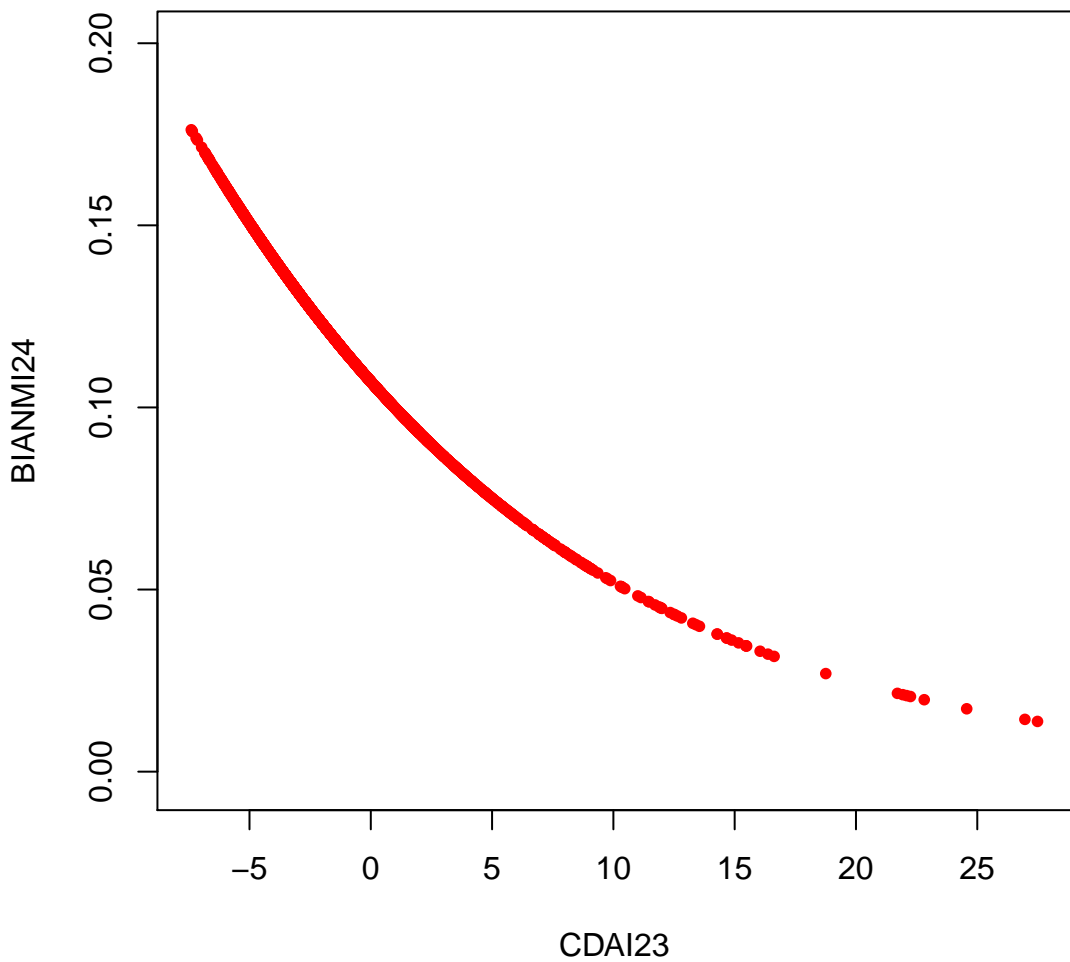

Supplement: S1 File — (ZIP) [file pone.0311168.s001.zip › CDAI/all/PROJ2_11_tbl/PROJ2_11_tbl_BIANMI24_CDAI23_XIYAN11_2_smooth.pdf]

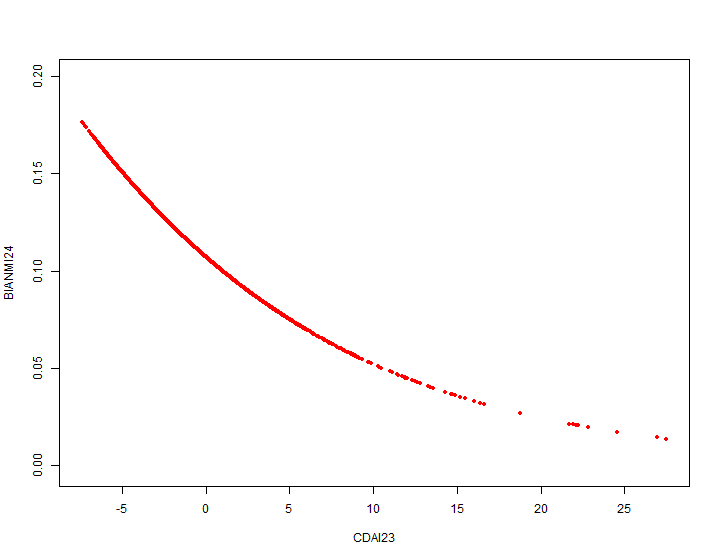

Supplement: S1 File — (ZIP) [file pone.0311168.s001.zip › CDAI/all/PROJ2_11_tbl/PROJ2_11_tbl_BIANMI24_CDAI23_XIYAN11_2_smooth.png]

BIANMI24

0.14  
0.12  
0.10  
0.08  
0.06

-5

0

5

10

15

20

25

CDAI23

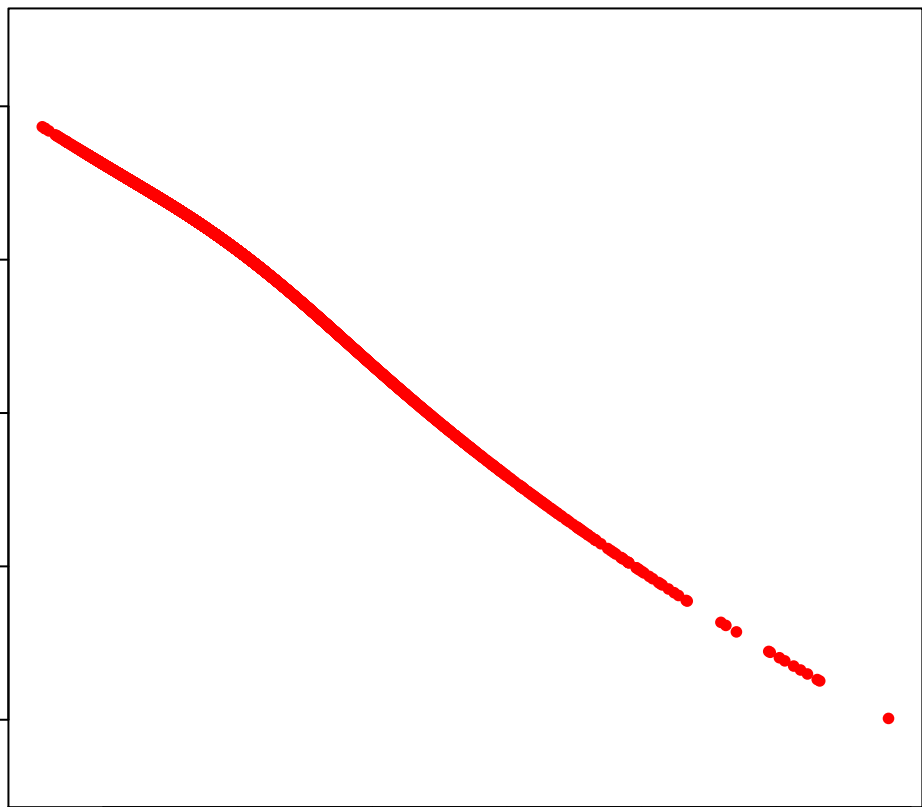

Supplement: S1 File — (ZIP) [file pone.0311168.s001.zip › CDAI/all/PROJ2_11_tbl/PROJ2_11_tbl_BIANMI24_CDAI23_XIYAN11_3_smooth.pdf]

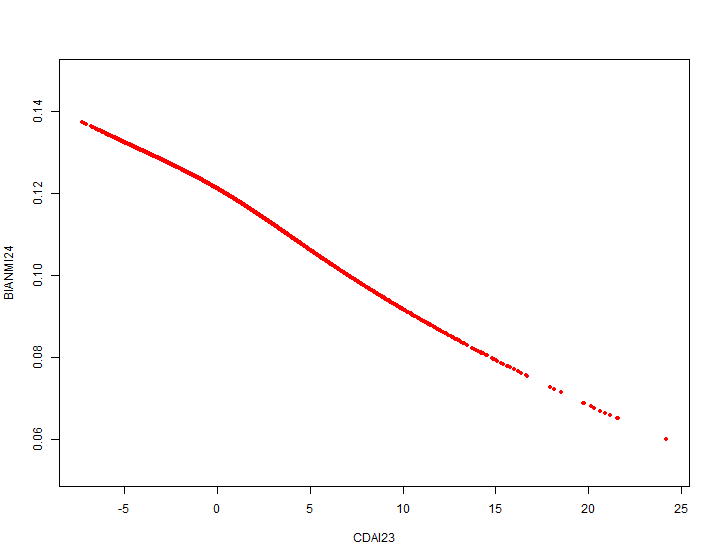

Supplement: S1 File — (ZIP) [file pone.0311168.s001.zip › CDAI/all/PROJ2_11_tbl/PROJ2_11_tbl_BIANMI24_CDAI23_XIYAN11_3_smooth.png]

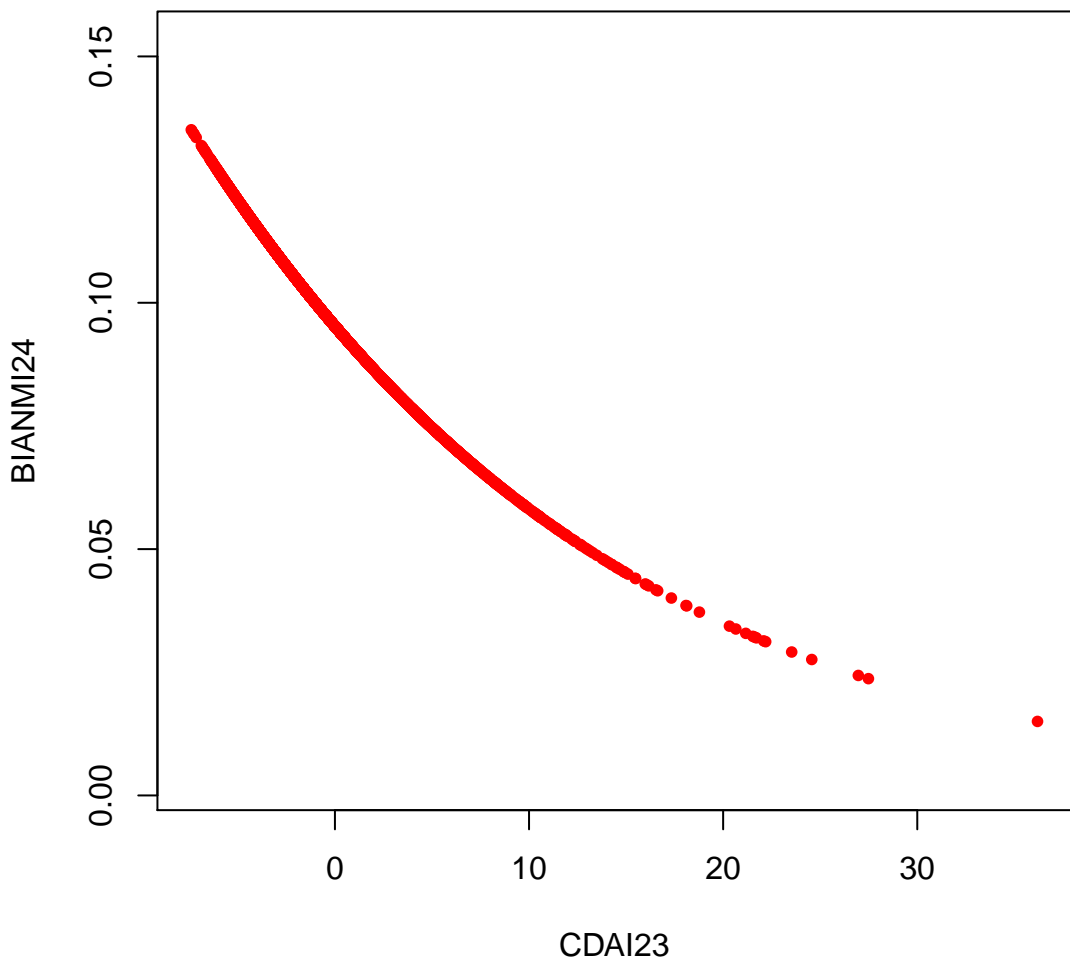

Supplement: S1 File — (ZIP) [file pone.0311168.s001.zip › CDAI/all/PROJ2_12_tbl/PROJ2_12_tbl_BIANMI24_CDAI23_GAOXUEYA12_1_smooth.pdf]

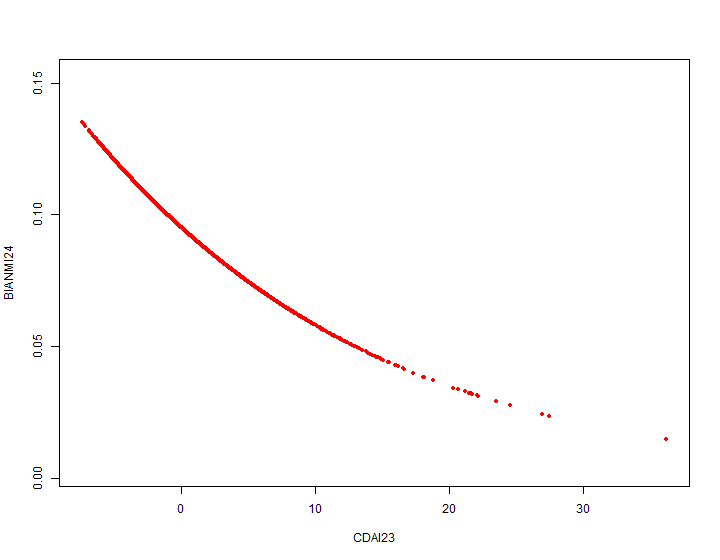

Supplement: S1 File — (ZIP) [file pone.0311168.s001.zip › CDAI/all/PROJ2_12_tbl/PROJ2_12_tbl_BIANMI24_CDAI23_GAOXUEYA12_1_smooth.png]

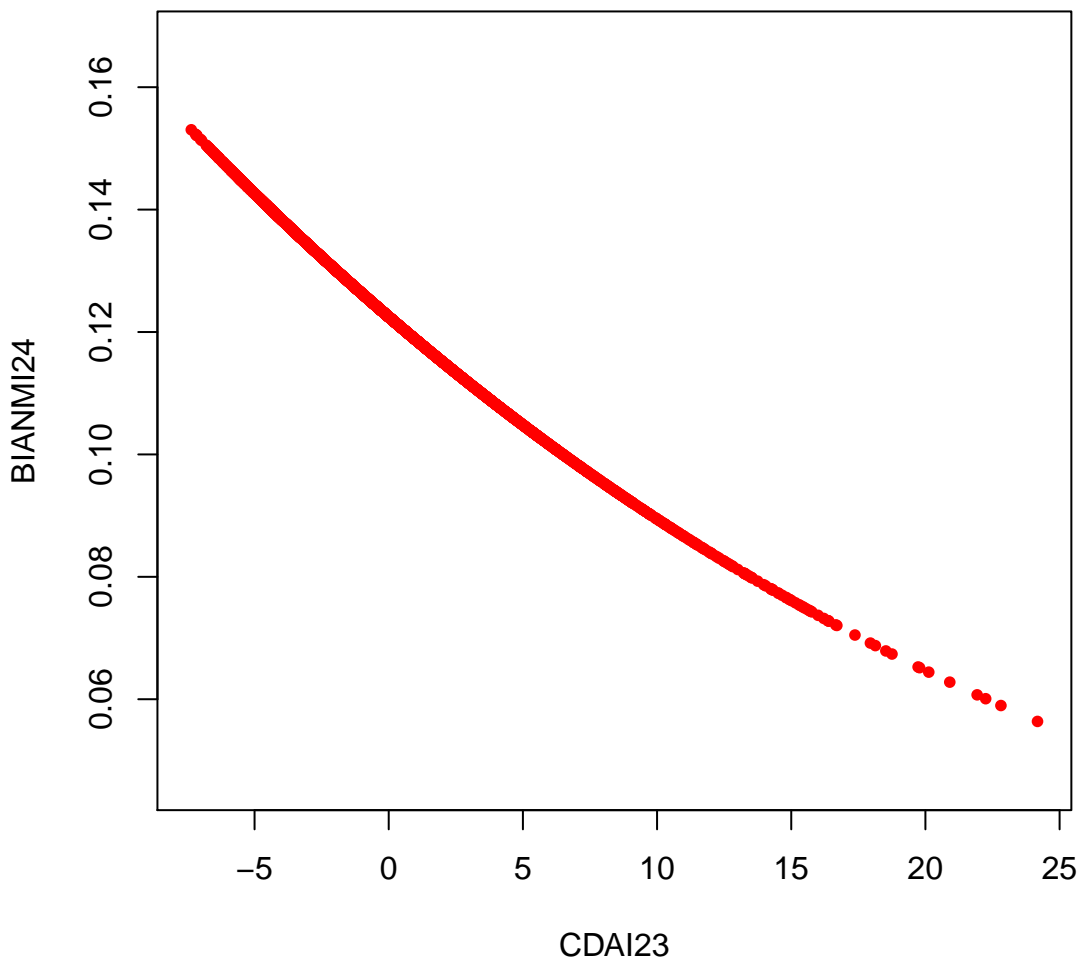

Supplement: S1 File — (ZIP) [file pone.0311168.s001.zip › CDAI/all/PROJ2_12_tbl/PROJ2_12_tbl_BIANMI24_CDAI23_GAOXUEYA12_2_smooth.pdf]

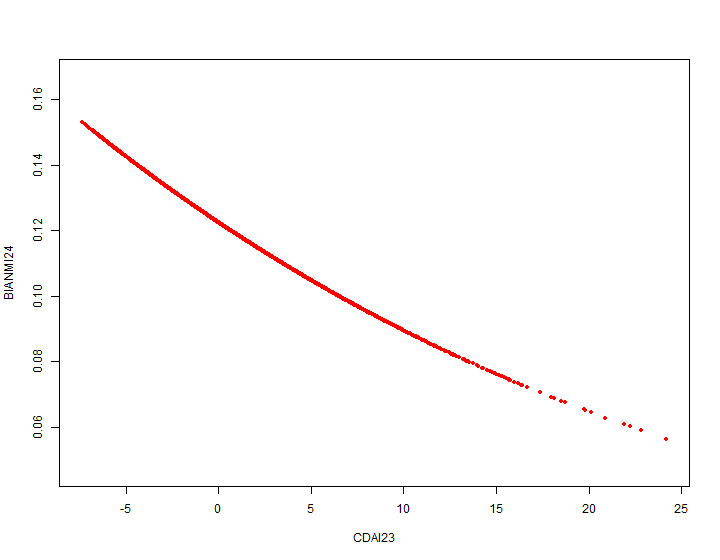

Supplement: S1 File — (ZIP) [file pone.0311168.s001.zip › CDAI/all/PROJ2_12_tbl/PROJ2_12_tbl_BIANMI24_CDAI23_GAOXUEYA12_2_smooth.png]

BIANMI24

GAOXUEYA12

1  
2

0.15  
0.10  
0.05  
0.00

0

10

20

30

CDAI23

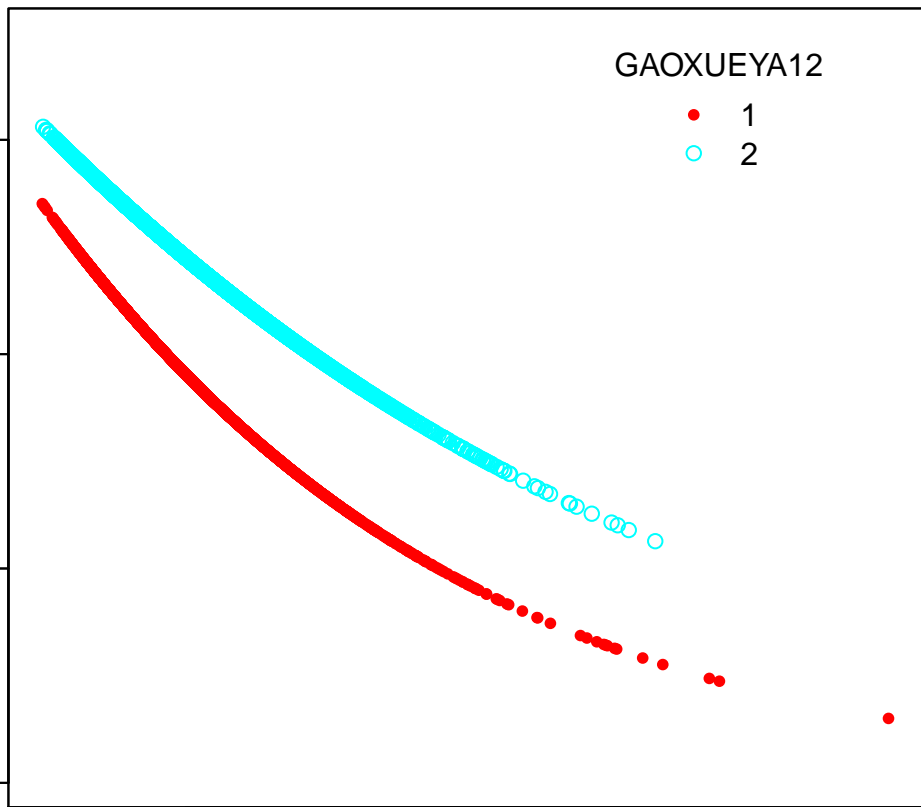

Supplement: S1 File — (ZIP) [file pone.0311168.s001.zip › CDAI/all/PROJ2_12_tbl/PROJ2_12_tbl_BIANMI24_CDAI23_smooth.pdf]

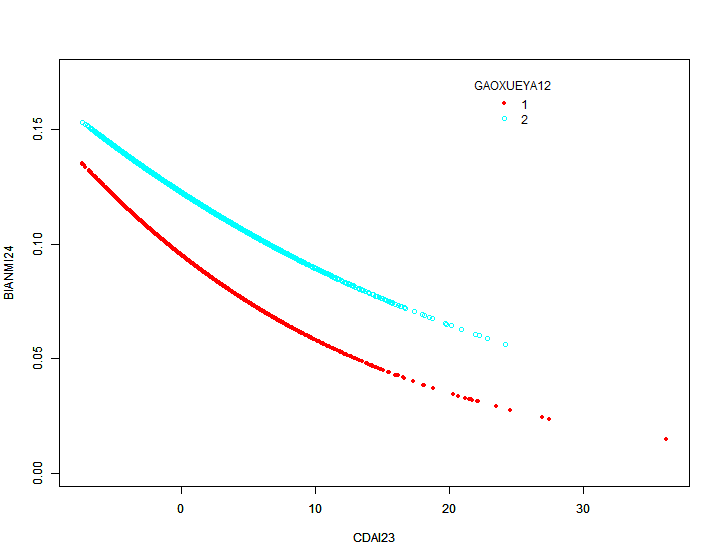

Supplement: S1 File — (ZIP) [file pone.0311168.s001.zip › CDAI/all/PROJ2_12_tbl/PROJ2_12_tbl_BIANMI24_CDAI23_smooth.png]

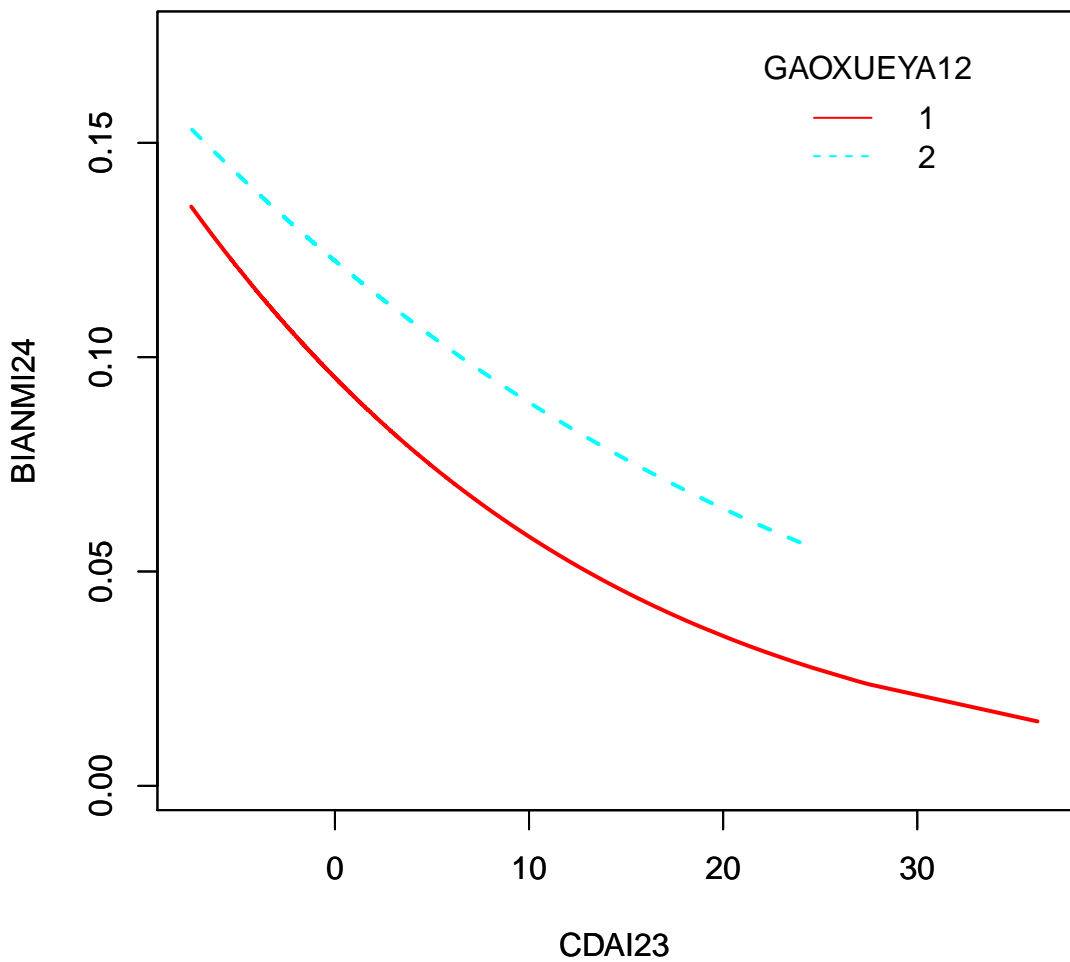

Supplement: S1 File — (ZIP) [file pone.0311168.s001.zip › CDAI/all/PROJ2_12_tbl/PROJ2_12_tbl_BIANMI24_CDAI23_smooth1.pdf]

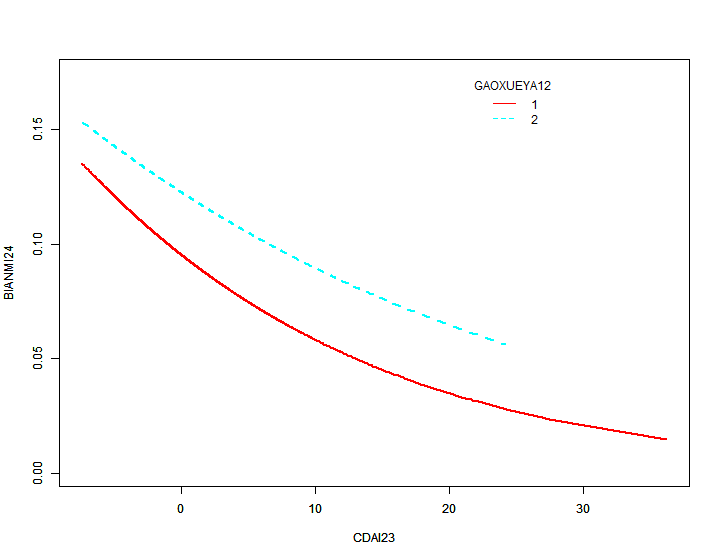

Supplement: S1 File — (ZIP) [file pone.0311168.s001.zip › CDAI/all/PROJ2_12_tbl/PROJ2_12_tbl_BIANMI24_CDAI23_smooth1.png]

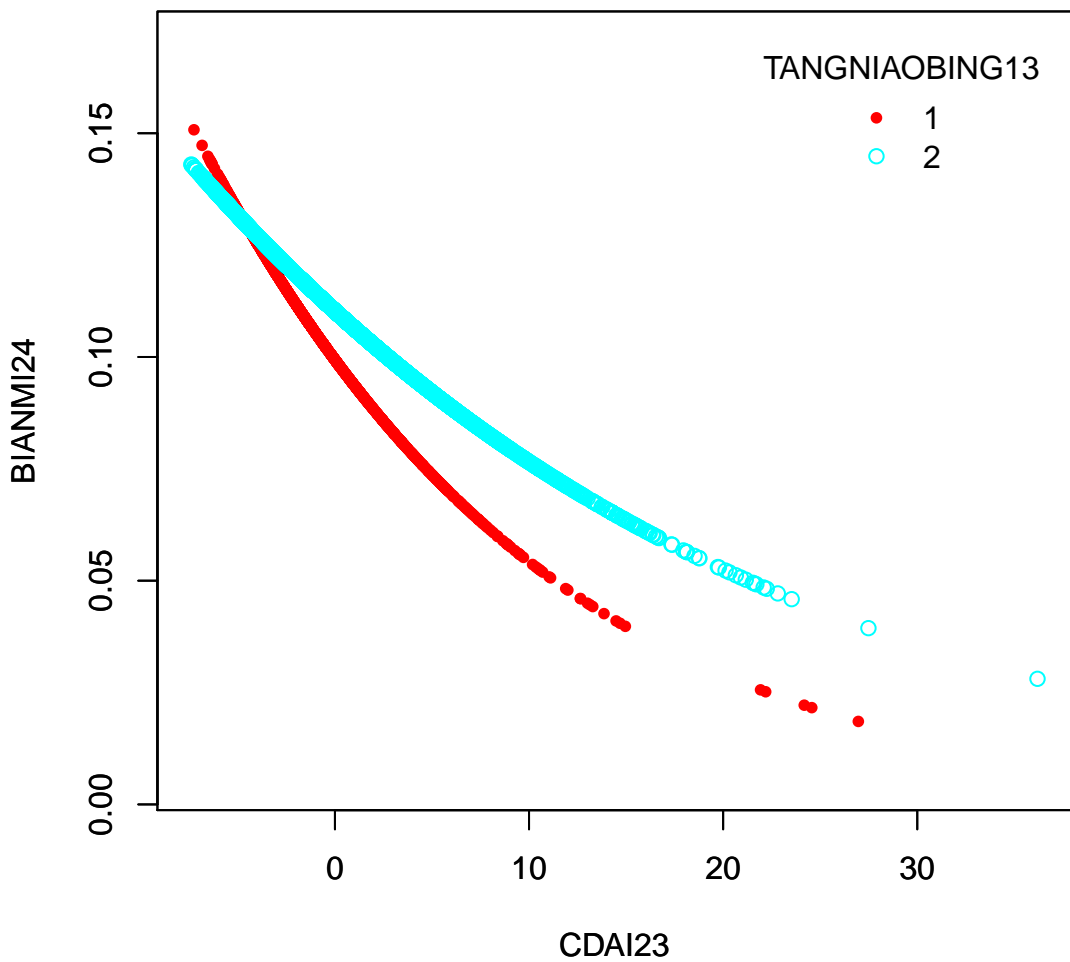

Supplement: S1 File — (ZIP) [file pone.0311168.s001.zip › CDAI/all/PROJ2_13_tbl/PROJ2_13_tbl_BIANMI24_CDAI23_smooth.pdf]

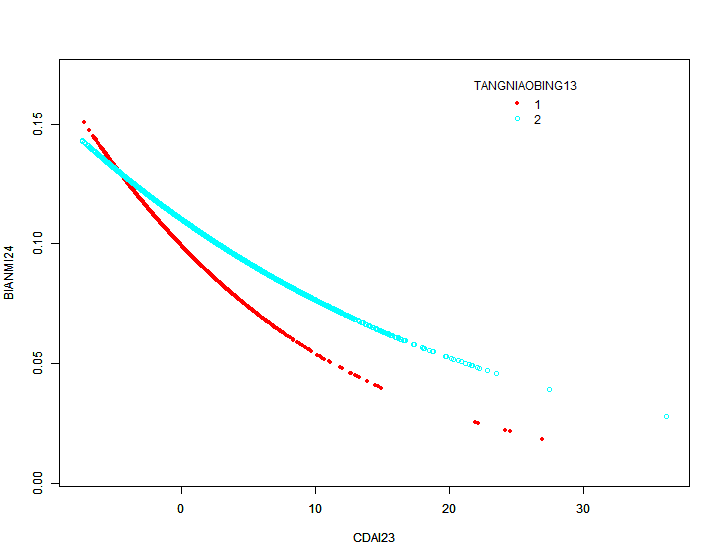

Supplement: S1 File — (ZIP) [file pone.0311168.s001.zip › CDAI/all/PROJ2_13_tbl/PROJ2_13_tbl_BIANMI24_CDAI23_smooth.png]

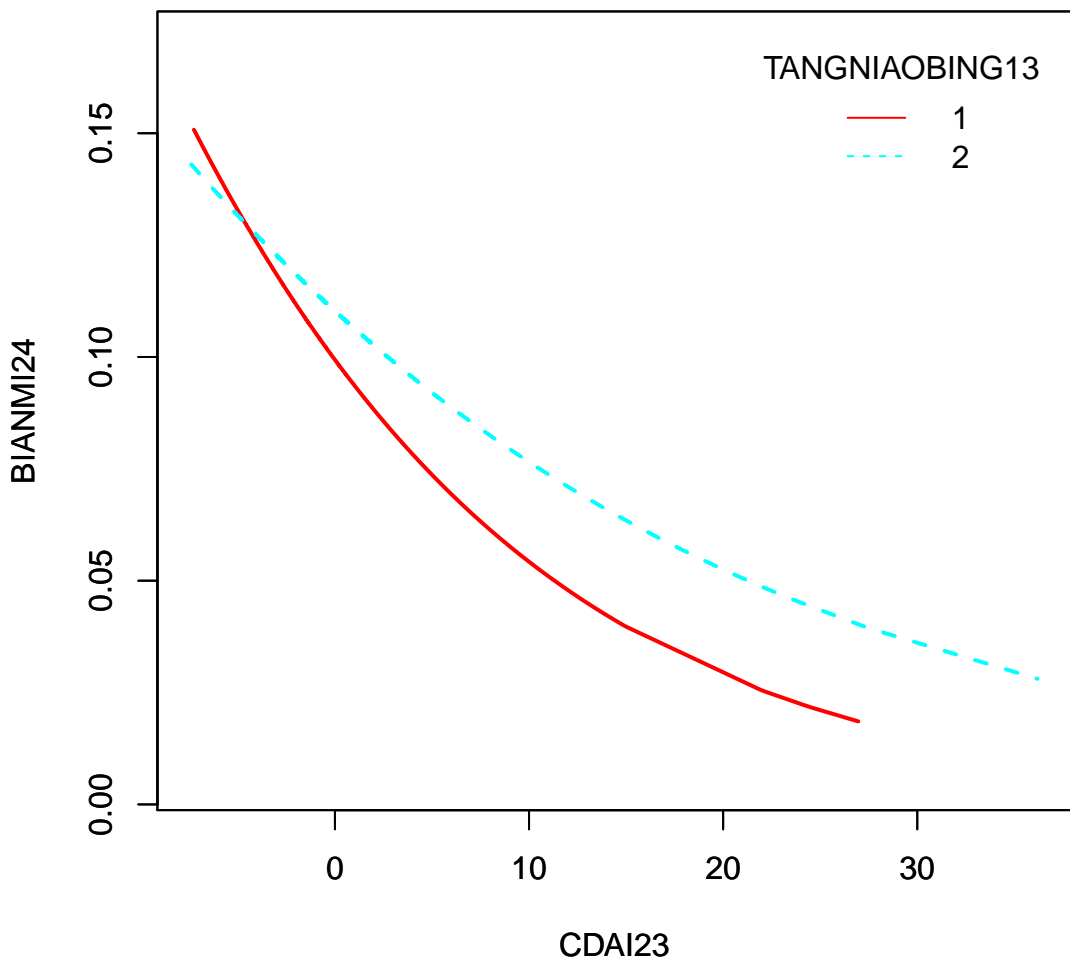

Supplement: S1 File — (ZIP) [file pone.0311168.s001.zip › CDAI/all/PROJ2_13_tbl/PROJ2_13_tbl_BIANMI24_CDAI23_smooth1.pdf]

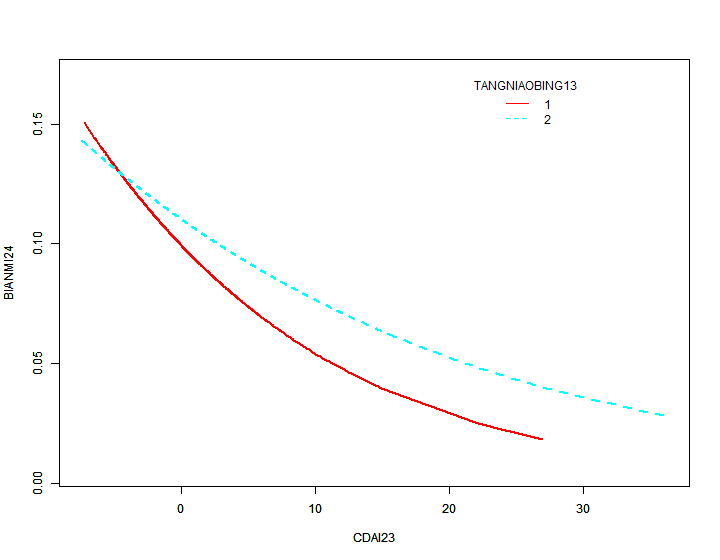

Supplement: S1 File — (ZIP) [file pone.0311168.s001.zip › CDAI/all/PROJ2_13_tbl/PROJ2_13_tbl_BIANMI24_CDAI23_smooth1.png]

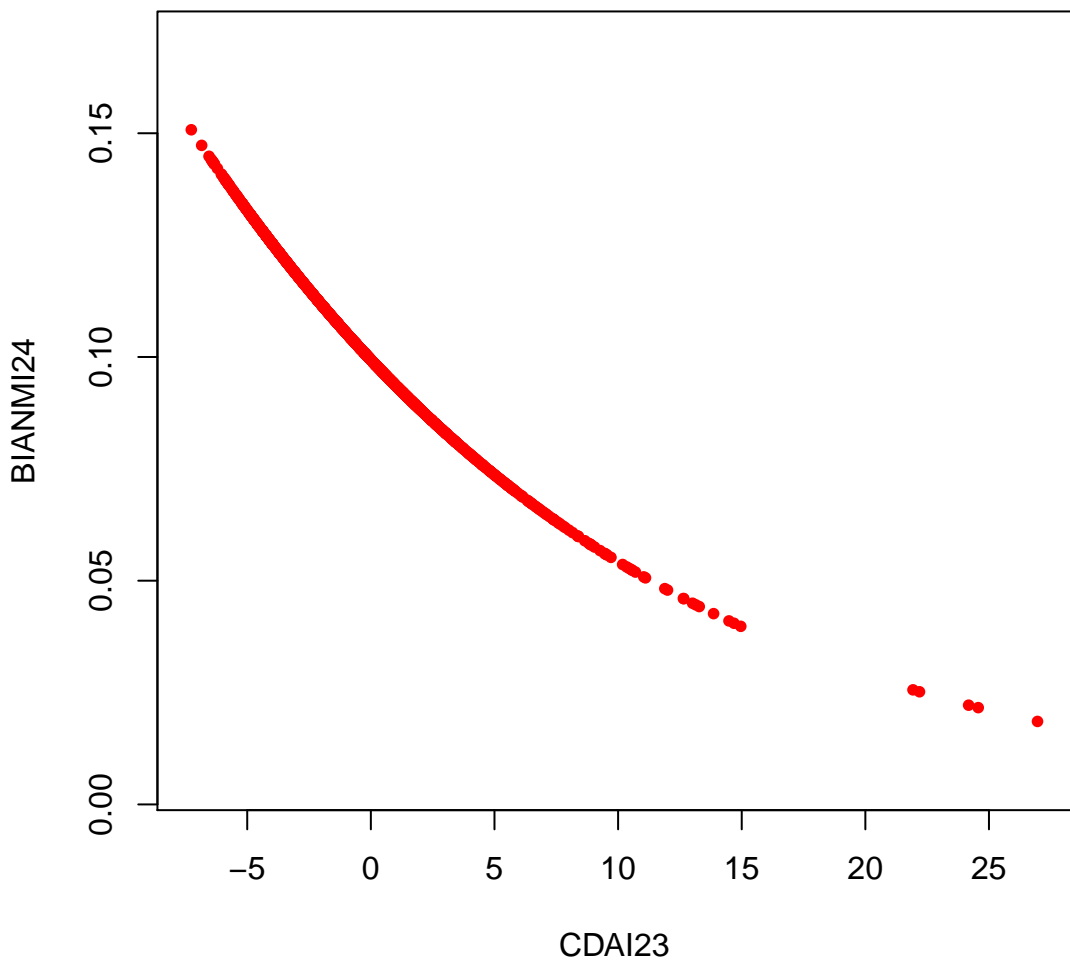

Supplement: S1 File — (ZIP) [file pone.0311168.s001.zip › CDAI/all/PROJ2_13_tbl/PROJ2_13_tbl_BIANMI24_CDAI23_TANGNIAOBING13_1_smooth.pdf]

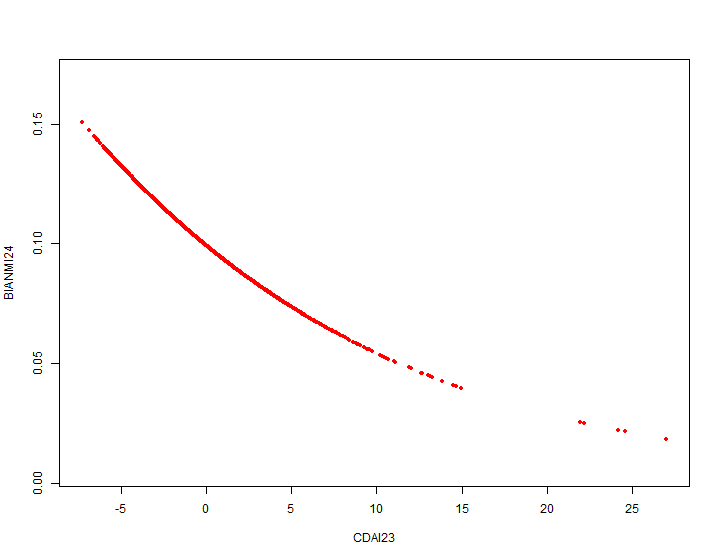

Supplement: S1 File — (ZIP) [file pone.0311168.s001.zip › CDAI/all/PROJ2_13_tbl/PROJ2_13_tbl_BIANMI24_CDAI23_TANGNIAOBING13_1_smooth.png]

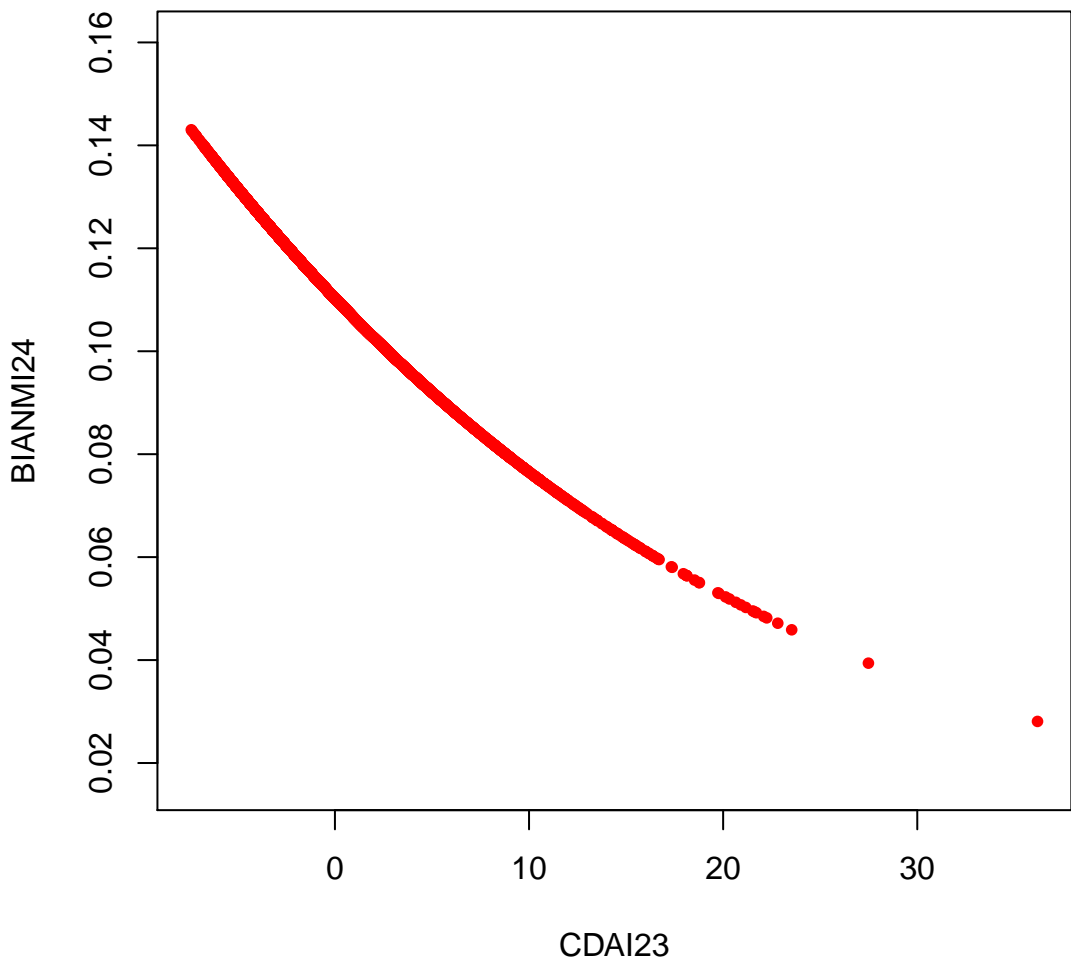

Supplement: S1 File — (ZIP) [file pone.0311168.s001.zip › CDAI/all/PROJ2_13_tbl/PROJ2_13_tbl_BIANMI24_CDAI23_TANGNIAOBING13_2_smooth.pdf]

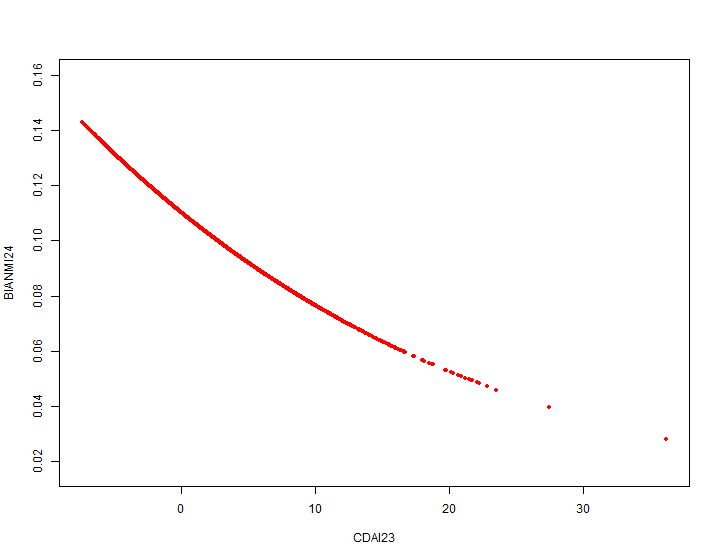

Supplement: S1 File — (ZIP) [file pone.0311168.s001.zip › CDAI/all/PROJ2_13_tbl/PROJ2_13_tbl_BIANMI24_CDAI23_TANGNIAOBING13_2_smooth.png]

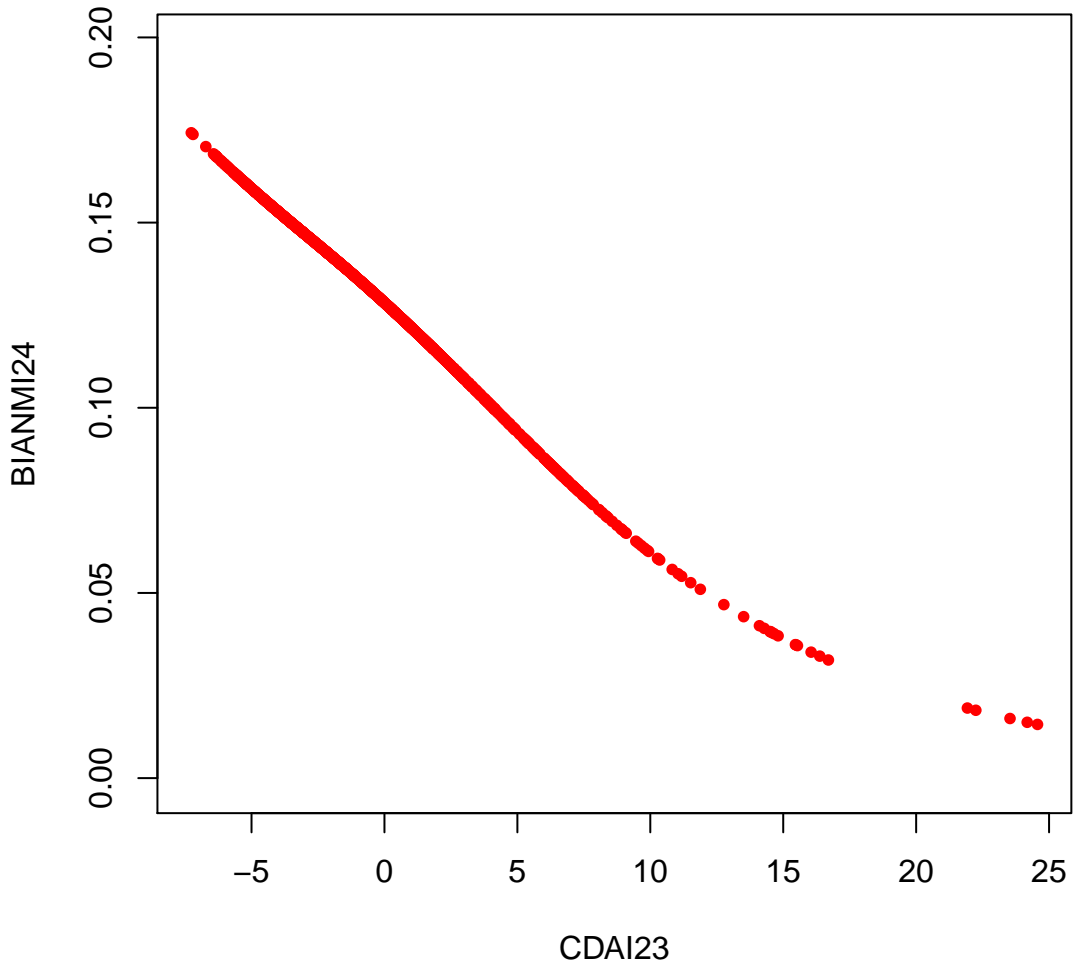

Supplement: S1 File — (ZIP) [file pone.0311168.s001.zip › CDAI/all/PROJ2_14_tbl/PROJ2_14_tbl_BIANMI24_CDAI23_FEIBING14_1_smooth.pdf]

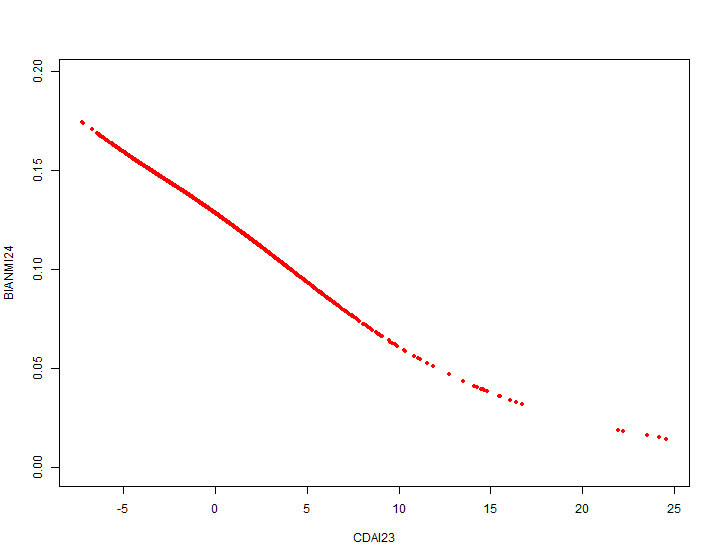

Supplement: S1 File — (ZIP) [file pone.0311168.s001.zip › CDAI/all/PROJ2_14_tbl/PROJ2_14_tbl_BIANMI24_CDAI23_FEIBING14_1_smooth.png]

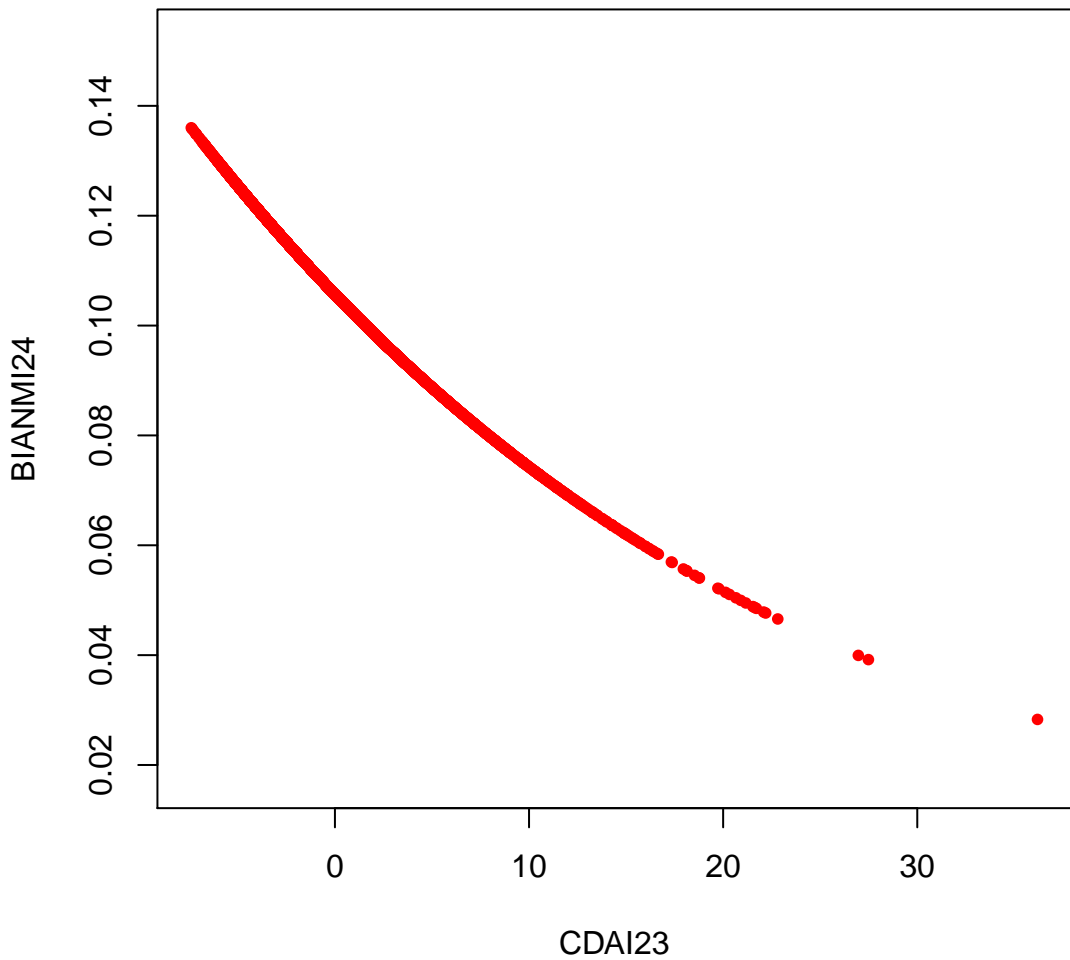

Supplement: S1 File — (ZIP) [file pone.0311168.s001.zip › CDAI/all/PROJ2_14_tbl/PROJ2_14_tbl_BIANMI24_CDAI23_FEIBING14_2_smooth.pdf]

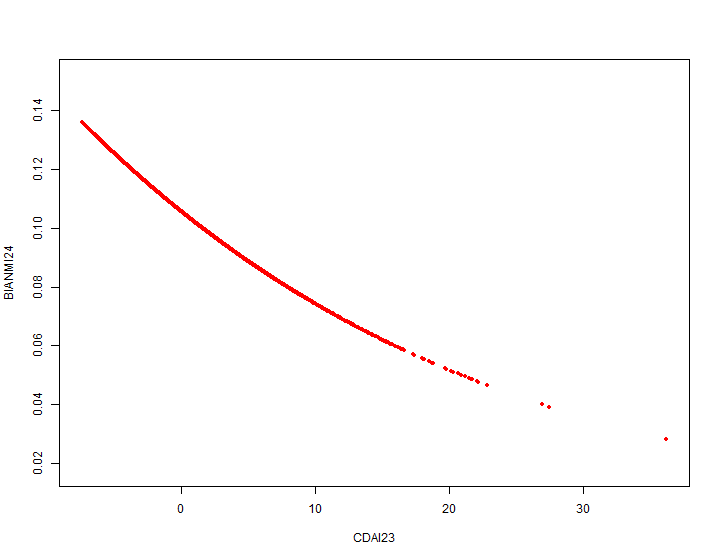

Supplement: S1 File — (ZIP) [file pone.0311168.s001.zip › CDAI/all/PROJ2_14_tbl/PROJ2_14_tbl_BIANMI24_CDAI23_FEIBING14_2_smooth.png]

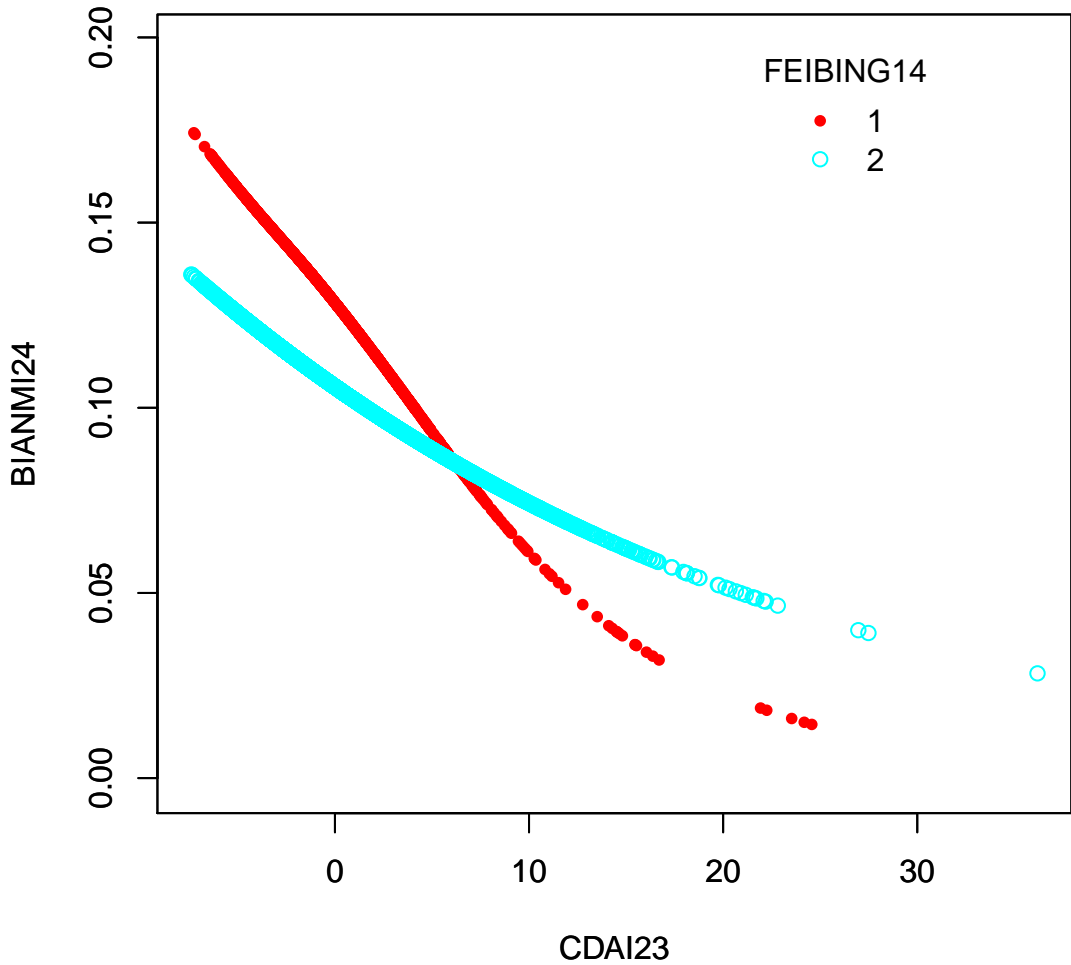

Supplement: S1 File — (ZIP) [file pone.0311168.s001.zip › CDAI/all/PROJ2_14_tbl/PROJ2_14_tbl_BIANMI24_CDAI23_smooth.pdf]

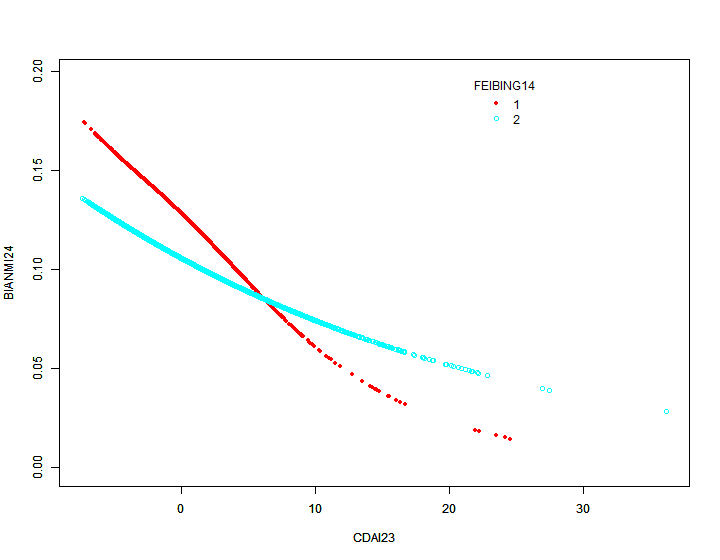

Supplement: S1 File — (ZIP) [file pone.0311168.s001.zip › CDAI/all/PROJ2_14_tbl/PROJ2_14_tbl_BIANMI24_CDAI23_smooth.png]

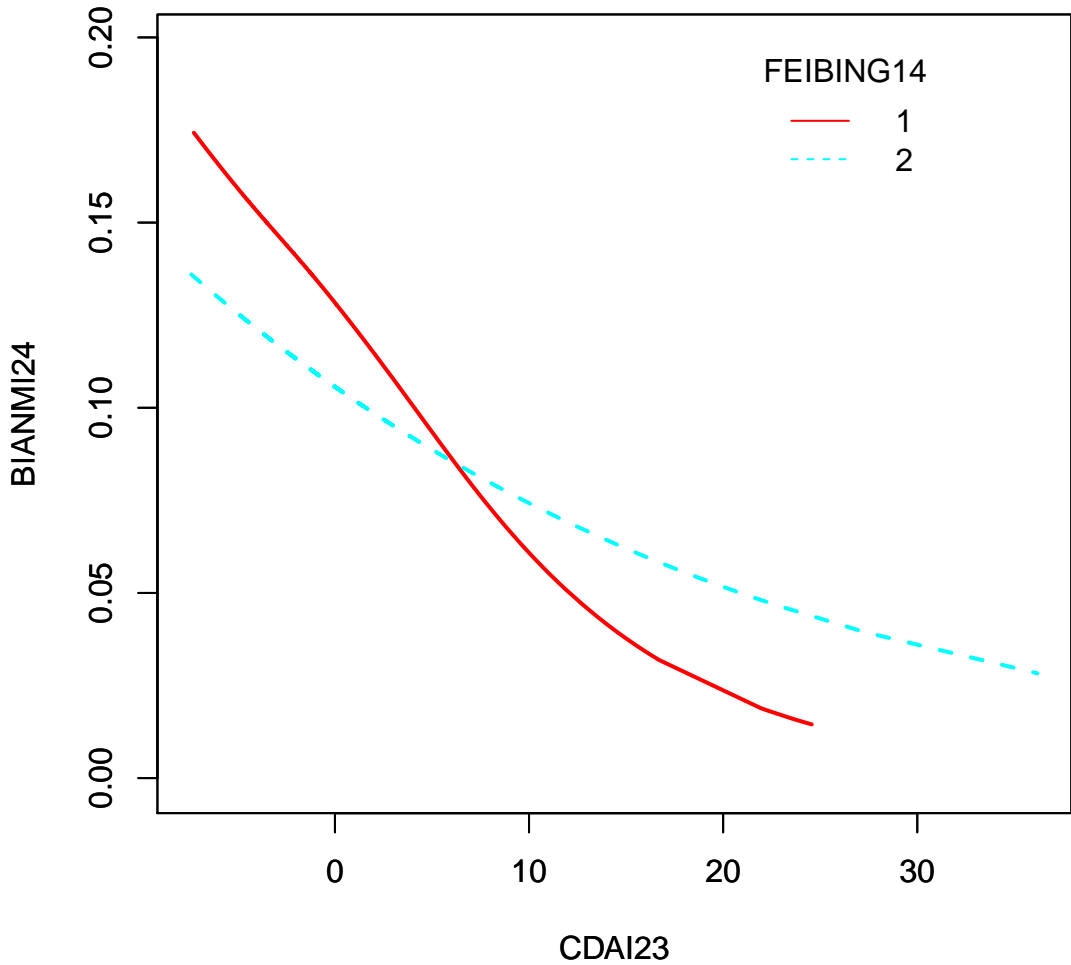

Supplement: S1 File — (ZIP) [file pone.0311168.s001.zip › CDAI/all/PROJ2_14_tbl/PROJ2_14_tbl_BIANMI24_CDAI23_smooth1.pdf]

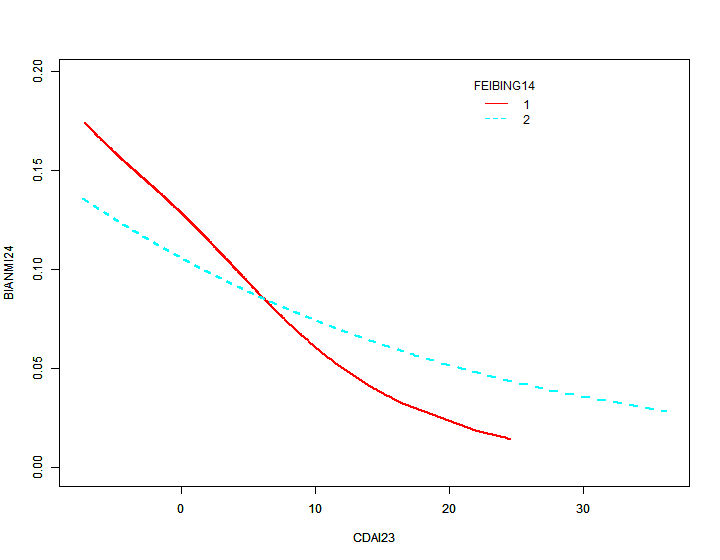

Supplement: S1 File — (ZIP) [file pone.0311168.s001.zip › CDAI/all/PROJ2_14_tbl/PROJ2_14_tbl_BIANMI24_CDAI23_smooth1.png]

BIANMI24

1.2  
1.0  
0.8  
0.6  
0.4  
0.2  
0.0

0

10

20

30

CDAI23

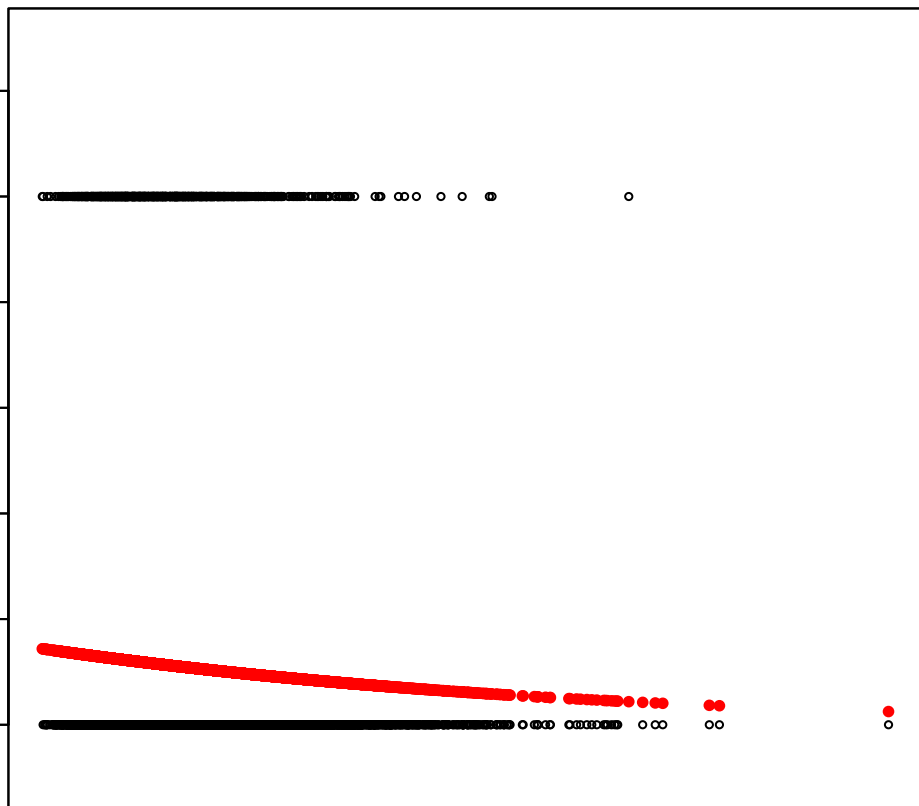

Supplement: S1 File — (ZIP) [file pone.0311168.s001.zip › CDAI/all/PROJ2_1_tbl/PROJ2_1_tbl_BIANMI24_CDAI23_scatter.pdf]

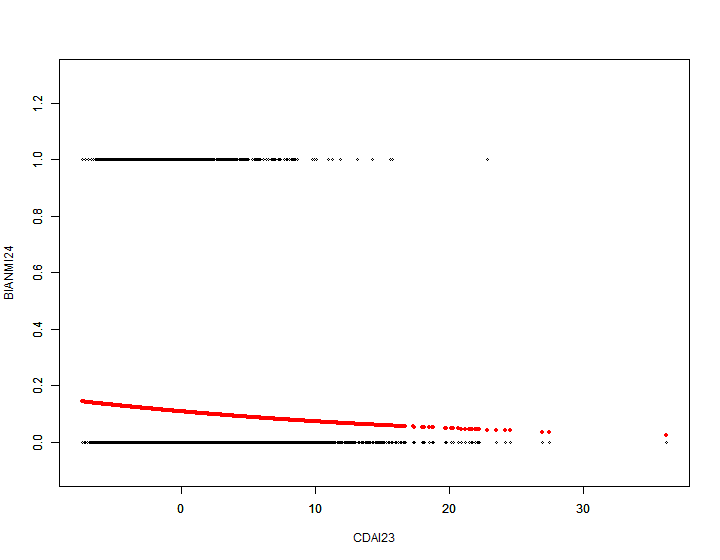

Supplement: S1 File — (ZIP) [file pone.0311168.s001.zip › CDAI/all/PROJ2_1_tbl/PROJ2_1_tbl_BIANMI24_CDAI23_scatter.png]

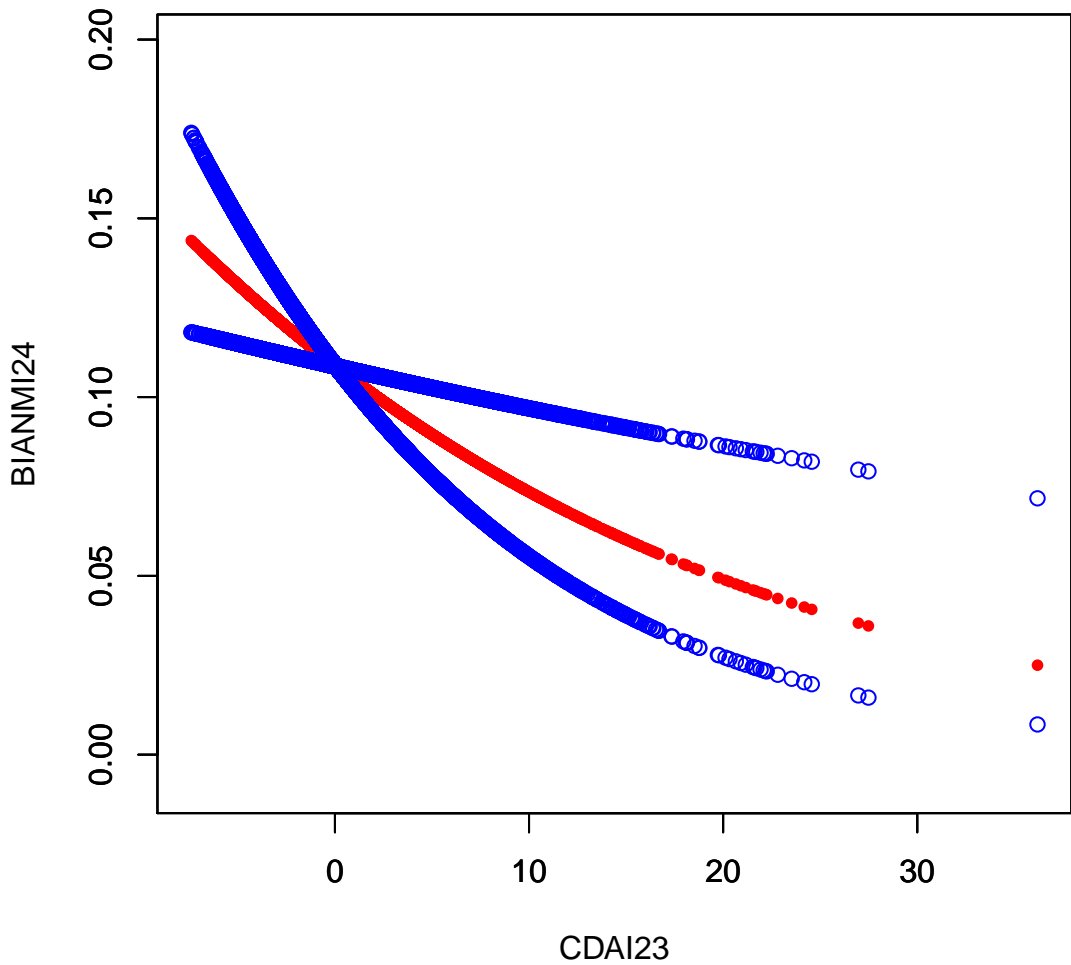

Supplement: S1 File — (ZIP) [file pone.0311168.s001.zip › CDAI/all/PROJ2_1_tbl/PROJ2_1_tbl_BIANMI24_CDAI23_smooth.pdf]

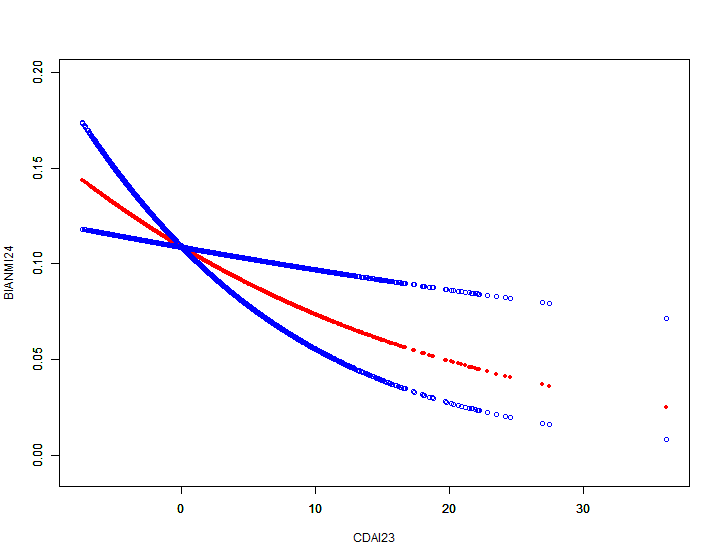

Supplement: S1 File — (ZIP) [file pone.0311168.s001.zip › CDAI/all/PROJ2_1_tbl/PROJ2_1_tbl_BIANMI24_CDAI23_smooth.png]

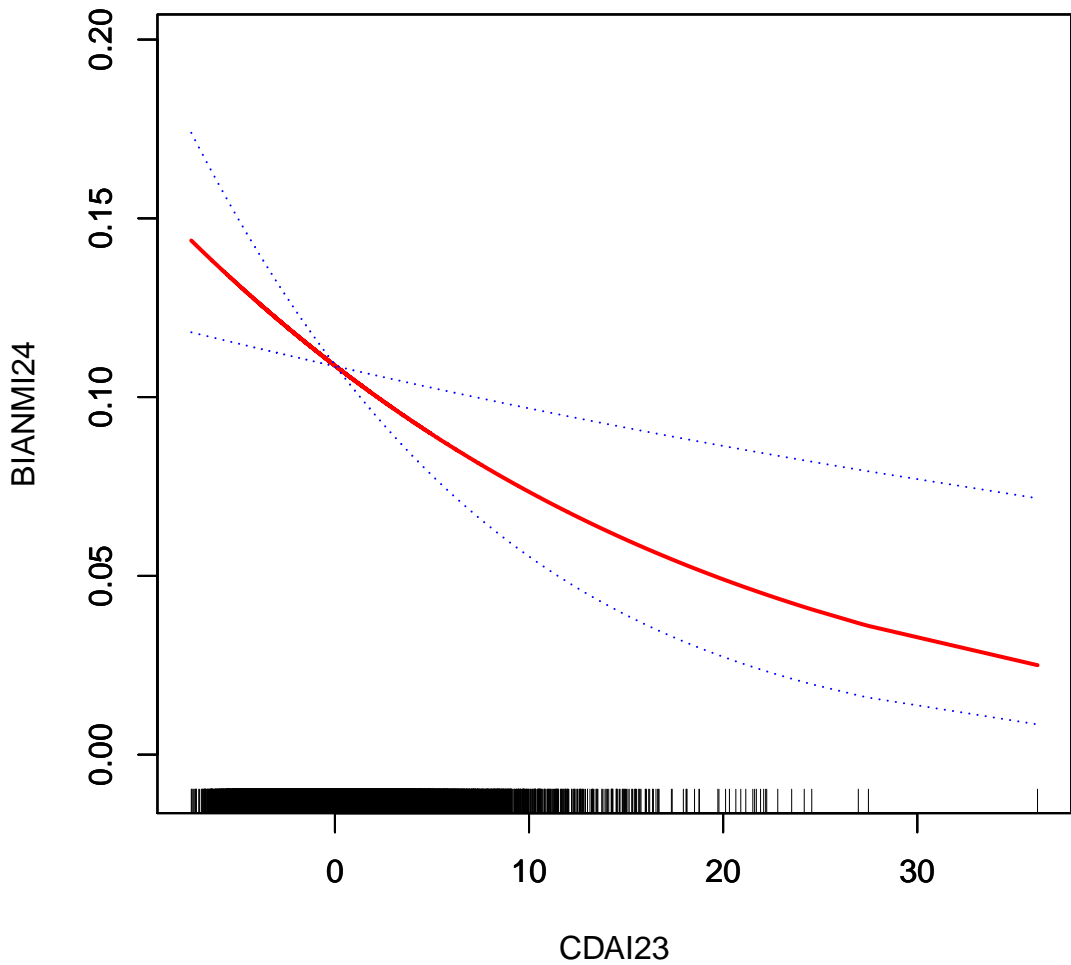

Supplement: S1 File — (ZIP) [file pone.0311168.s001.zip › CDAI/all/PROJ2_1_tbl/PROJ2_1_tbl_BIANMI24_CDAI23_smooth1.pdf]

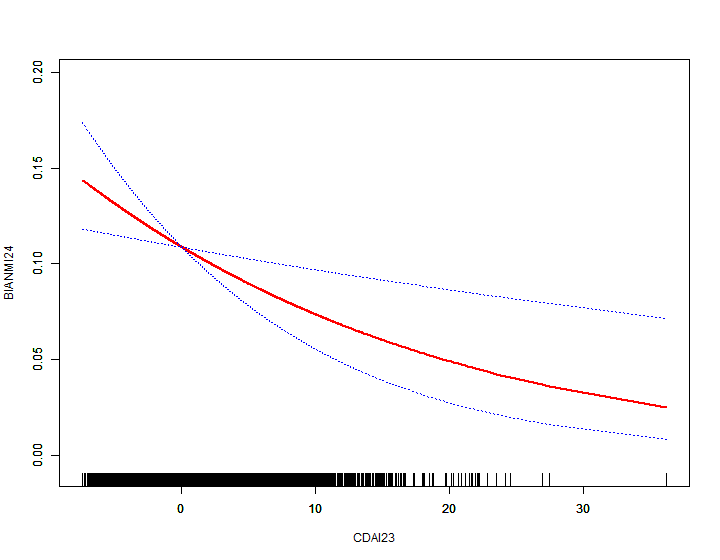

Supplement: S1 File — (ZIP) [file pone.0311168.s001.zip › CDAI/all/PROJ2_1_tbl/PROJ2_1_tbl_BIANMI24_CDAI23_smooth1.png]

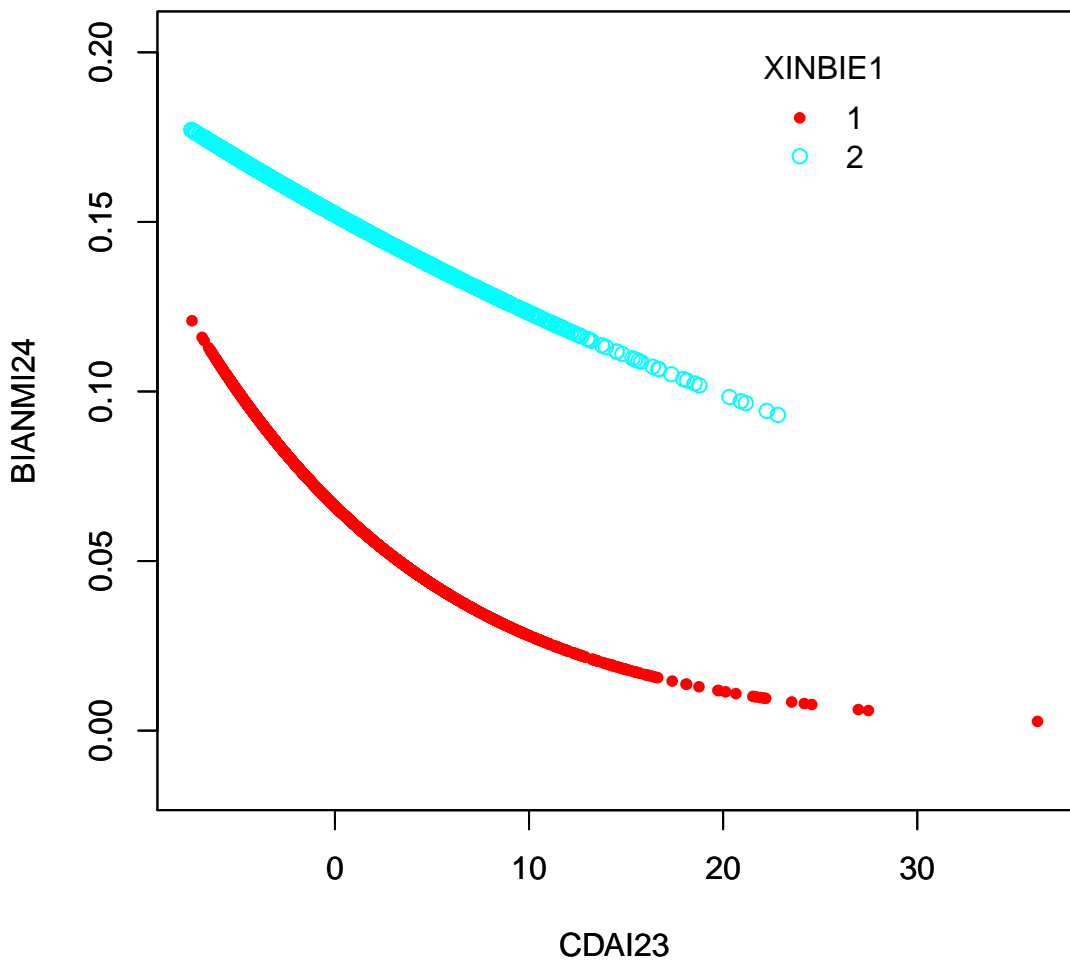

Supplement: S1 File — (ZIP) [file pone.0311168.s001.zip › CDAI/all/PROJ2_2_tbl/PROJ2_2_tbl_BIANMI24_CDAI23_smooth.pdf]

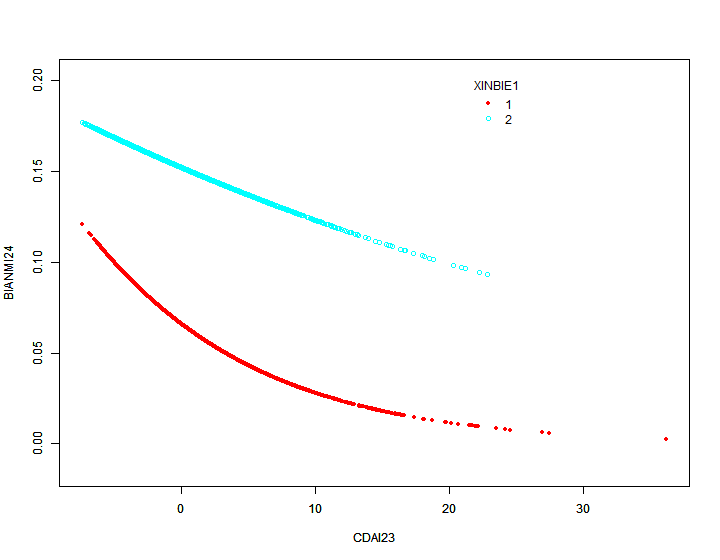

Supplement: S1 File — (ZIP) [file pone.0311168.s001.zip › CDAI/all/PROJ2_2_tbl/PROJ2_2_tbl_BIANMI24_CDAI23_smooth.png]

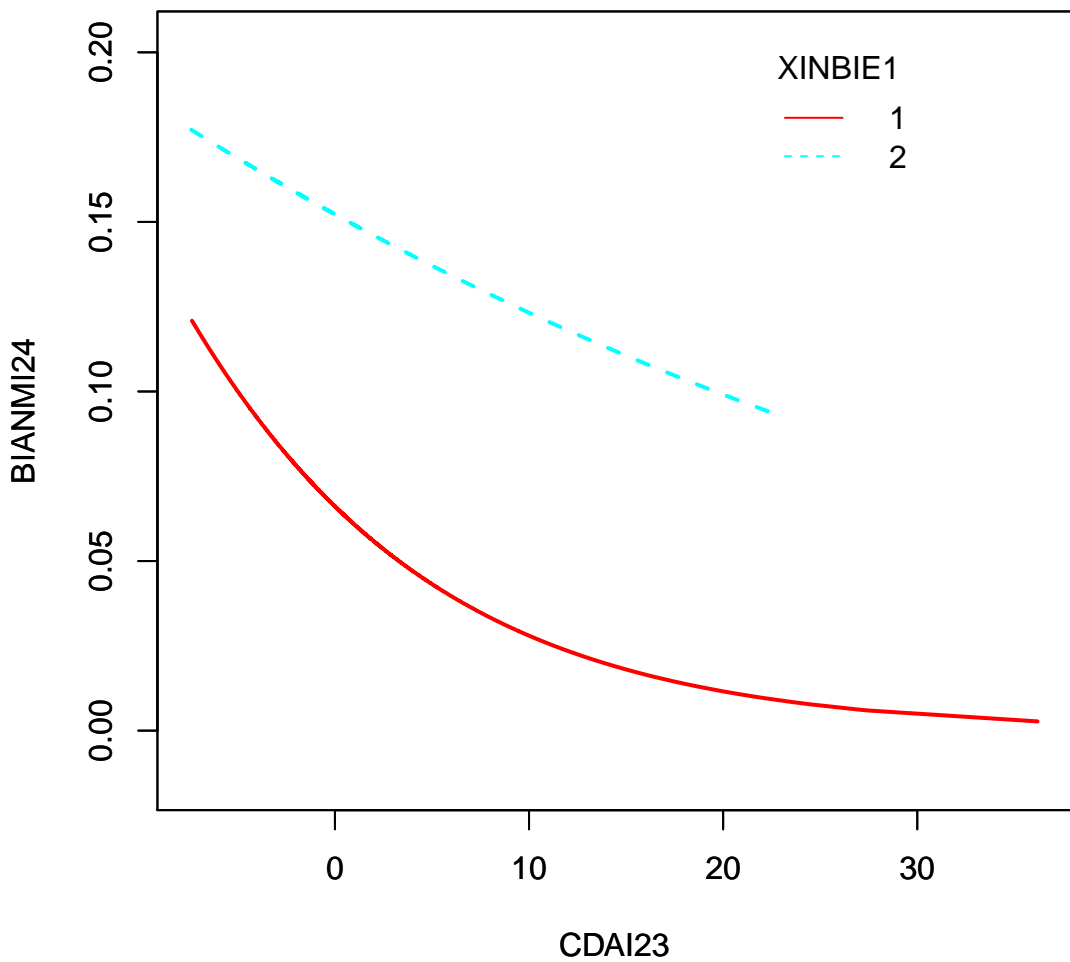

Supplement: S1 File — (ZIP) [file pone.0311168.s001.zip › CDAI/all/PROJ2_2_tbl/PROJ2_2_tbl_BIANMI24_CDAI23_smooth1.pdf]

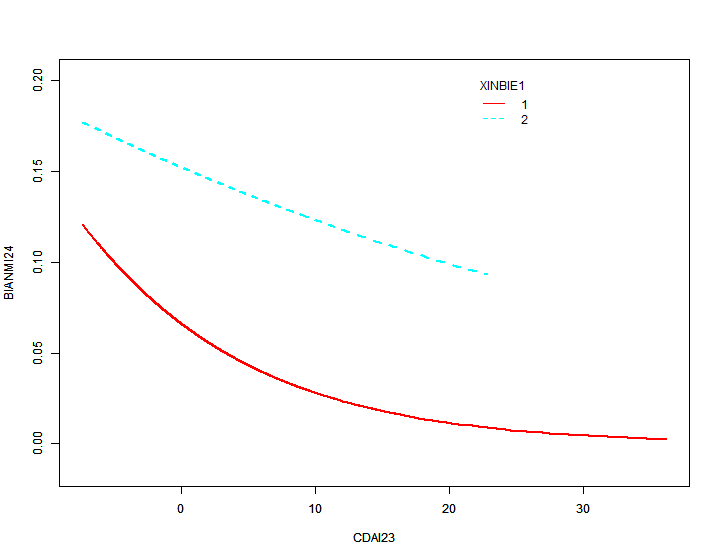

Supplement: S1 File — (ZIP) [file pone.0311168.s001.zip › CDAI/all/PROJ2_2_tbl/PROJ2_2_tbl_BIANMI24_CDAI23_smooth1.png]

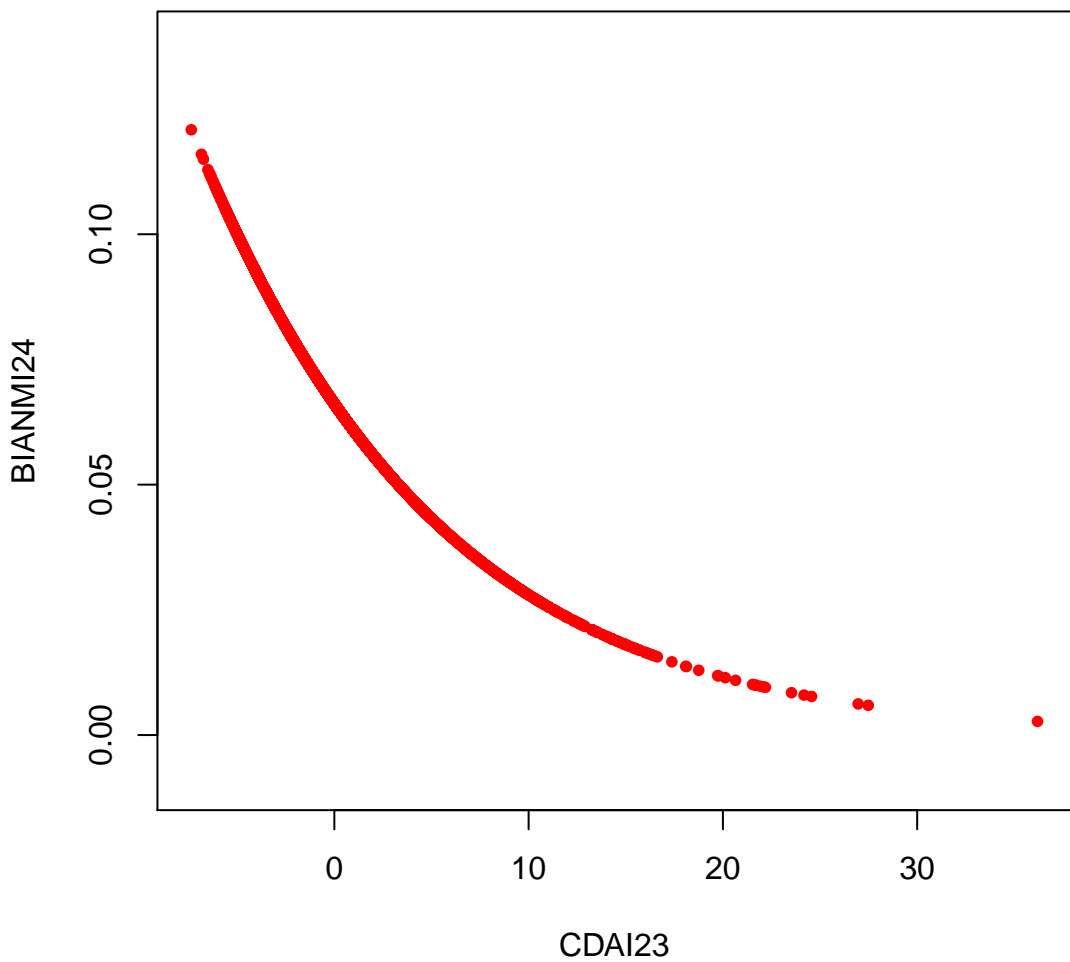

Supplement: S1 File — (ZIP) [file pone.0311168.s001.zip › CDAI/all/PROJ2_2_tbl/PROJ2_2_tbl_BIANMI24_CDAI23_XINBIE1_1_smooth.pdf]

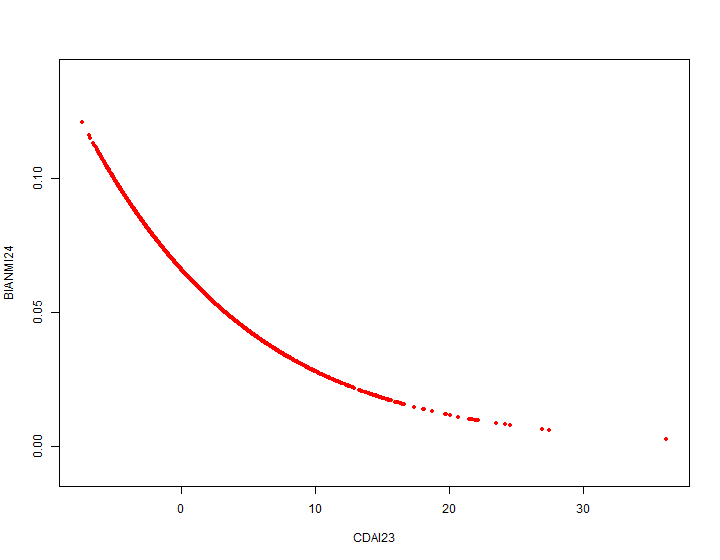

Supplement: S1 File — (ZIP) [file pone.0311168.s001.zip › CDAI/all/PROJ2_2_tbl/PROJ2_2_tbl_BIANMI24_CDAI23_XINBIE1_1_smooth.png]

BIANMI24

0.18  
0.16  
0.14  
0.12  
0.10

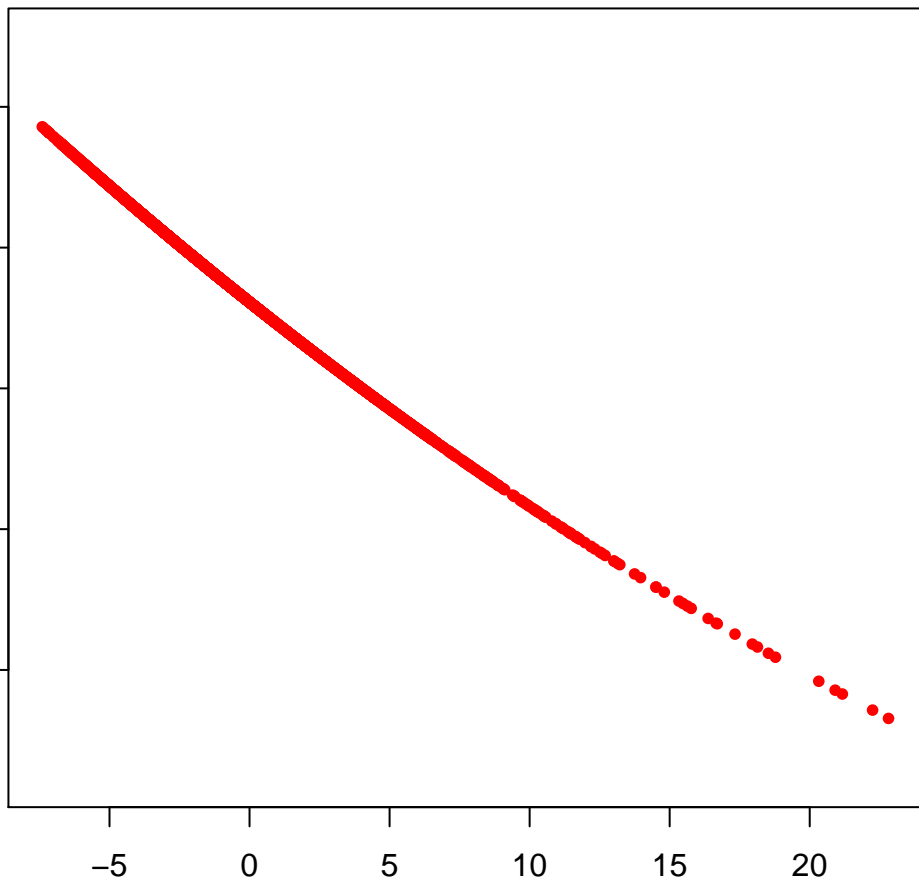

CDAI23

Supplement: S1 File — (ZIP) [file pone.0311168.s001.zip › CDAI/all/PROJ2_2_tbl/PROJ2_2_tbl_BIANMI24_CDAI23_XINBIE1_2_smooth.pdf]

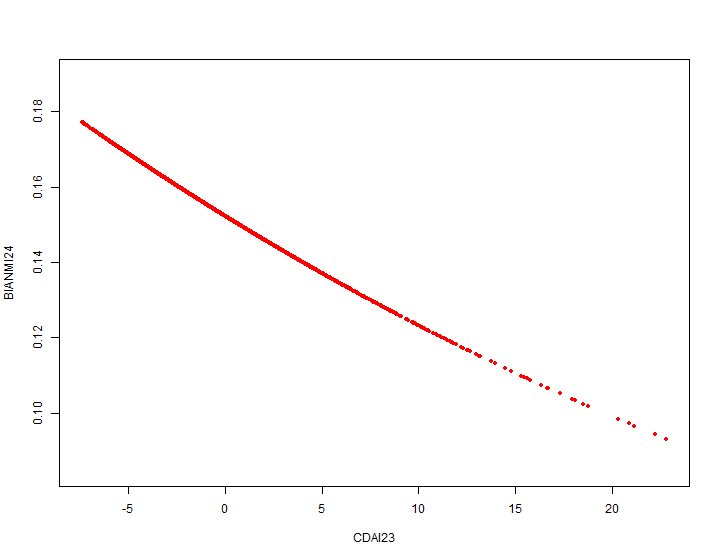

Supplement: S1 File — (ZIP) [file pone.0311168.s001.zip › CDAI/all/PROJ2_2_tbl/PROJ2_2_tbl_BIANMI24_CDAI23_XINBIE1_2_smooth.png]

BIANMI24

0.20

0.15

0.10

0.05

-5

0

5

10

15

20

CDAI23

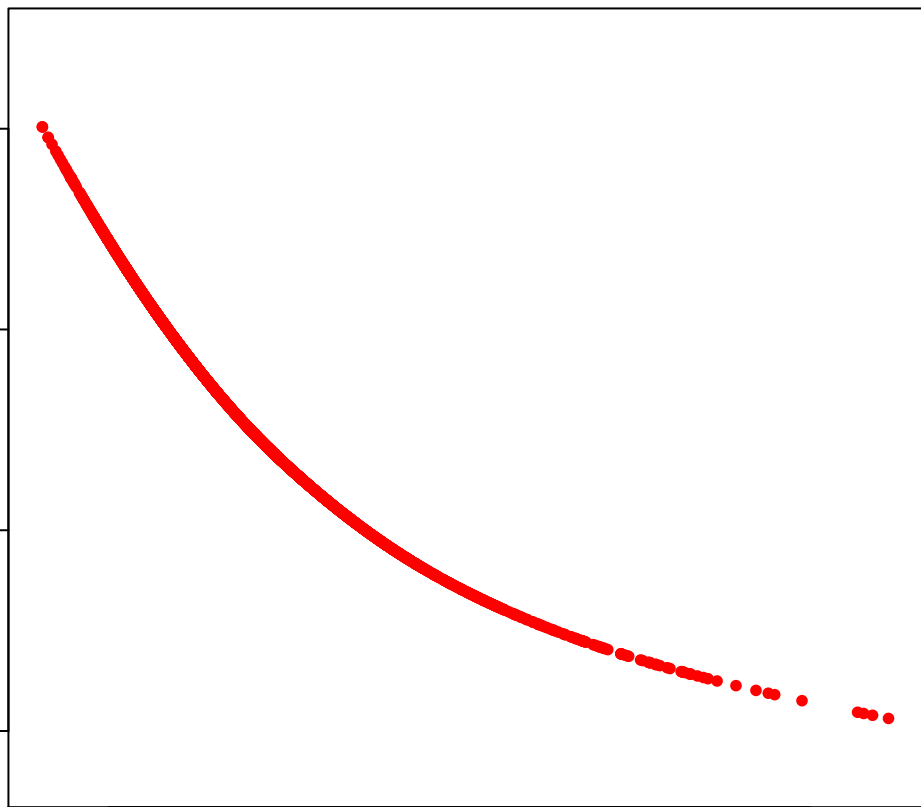

Supplement: S1 File — (ZIP) [file pone.0311168.s001.zip › CDAI/all/PROJ2_3_tbl1/PROJ2_3_tbl1_BIANMI24_CDAI23_AGE_1_smooth.pdf]

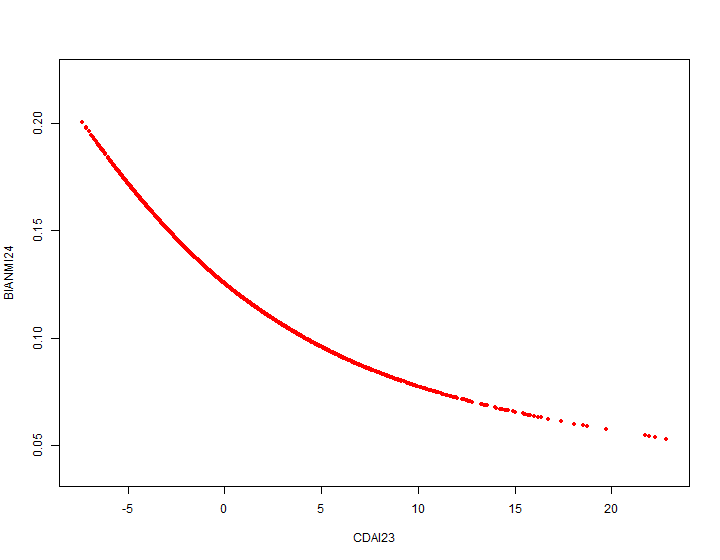

Supplement: S1 File — (ZIP) [file pone.0311168.s001.zip › CDAI/all/PROJ2_3_tbl1/PROJ2_3_tbl1_BIANMI24_CDAI23_AGE_1_smooth.png]

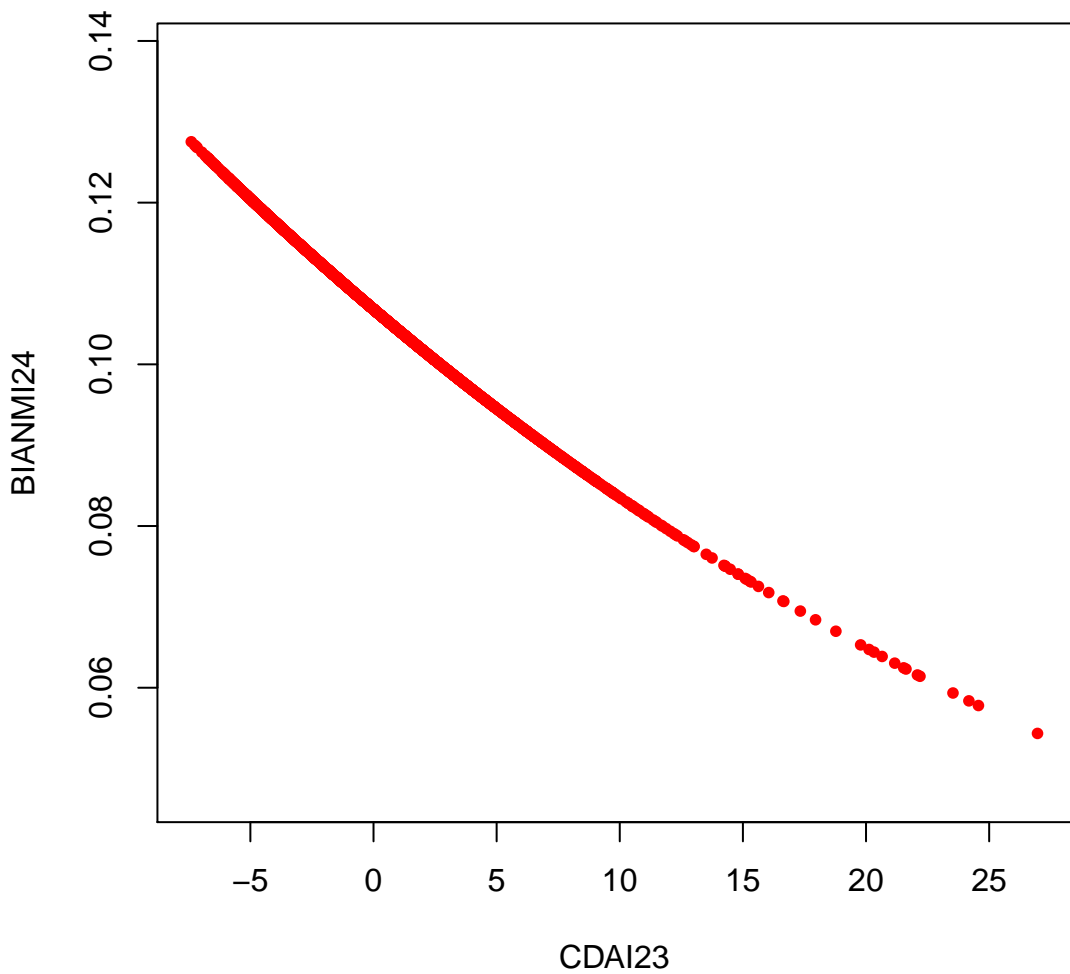

Supplement: S1 File — (ZIP) [file pone.0311168.s001.zip › CDAI/all/PROJ2_3_tbl1/PROJ2_3_tbl1_BIANMI24_CDAI23_AGE_2_smooth.pdf]

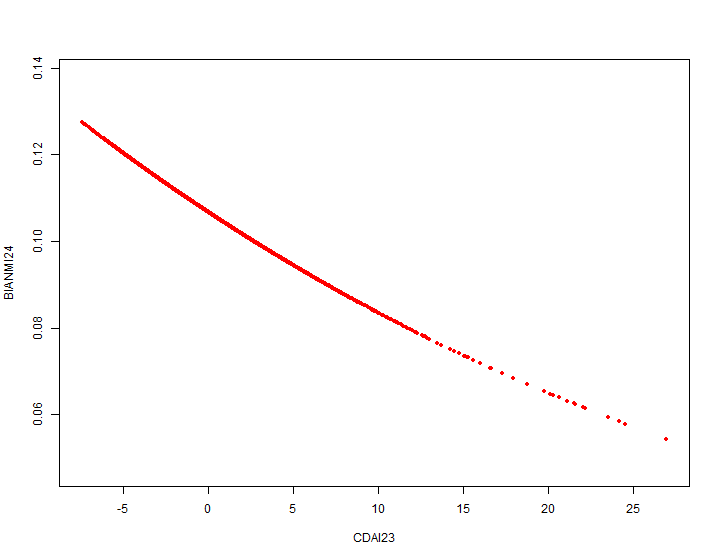

Supplement: S1 File — (ZIP) [file pone.0311168.s001.zip › CDAI/all/PROJ2_3_tbl1/PROJ2_3_tbl1_BIANMI24_CDAI23_AGE_2_smooth.png]

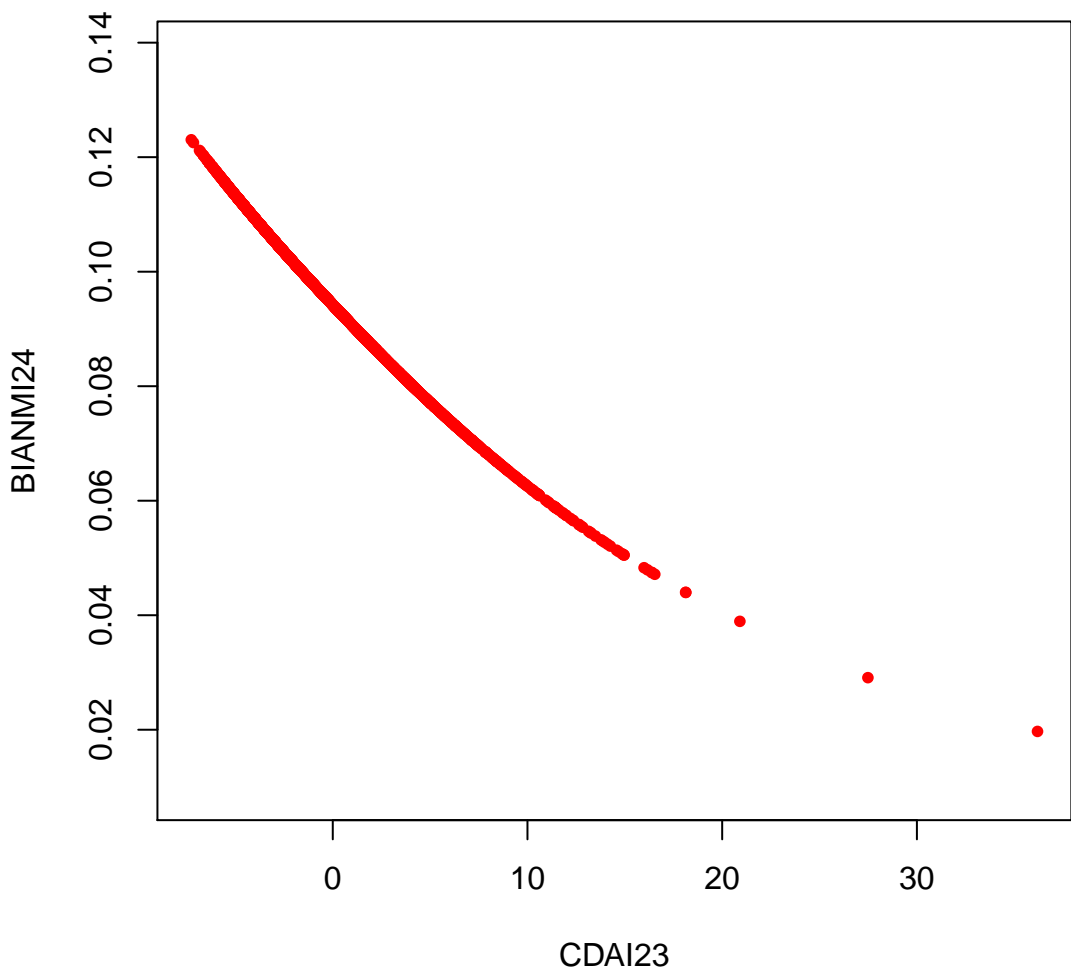

Supplement: S1 File — (ZIP) [file pone.0311168.s001.zip › CDAI/all/PROJ2_3_tbl1/PROJ2_3_tbl1_BIANMI24_CDAI23_AGE_3_smooth.pdf]

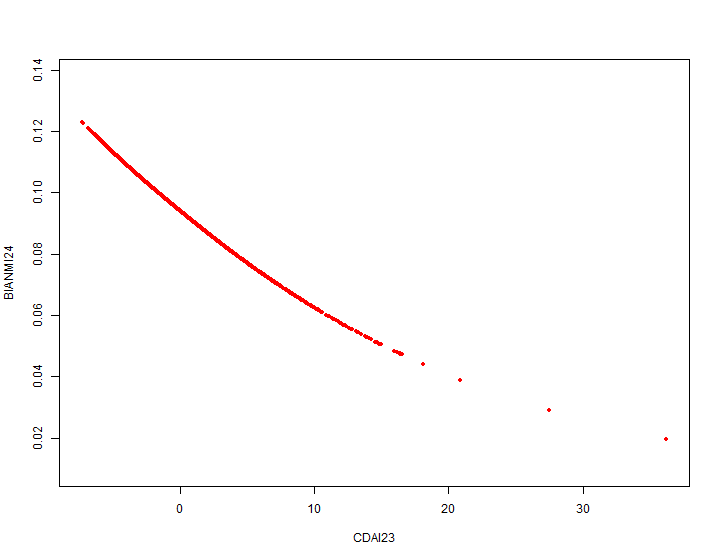

Supplement: S1 File — (ZIP) [file pone.0311168.s001.zip › CDAI/all/PROJ2_3_tbl1/PROJ2_3_tbl1_BIANMI24_CDAI23_AGE_3_smooth.png]

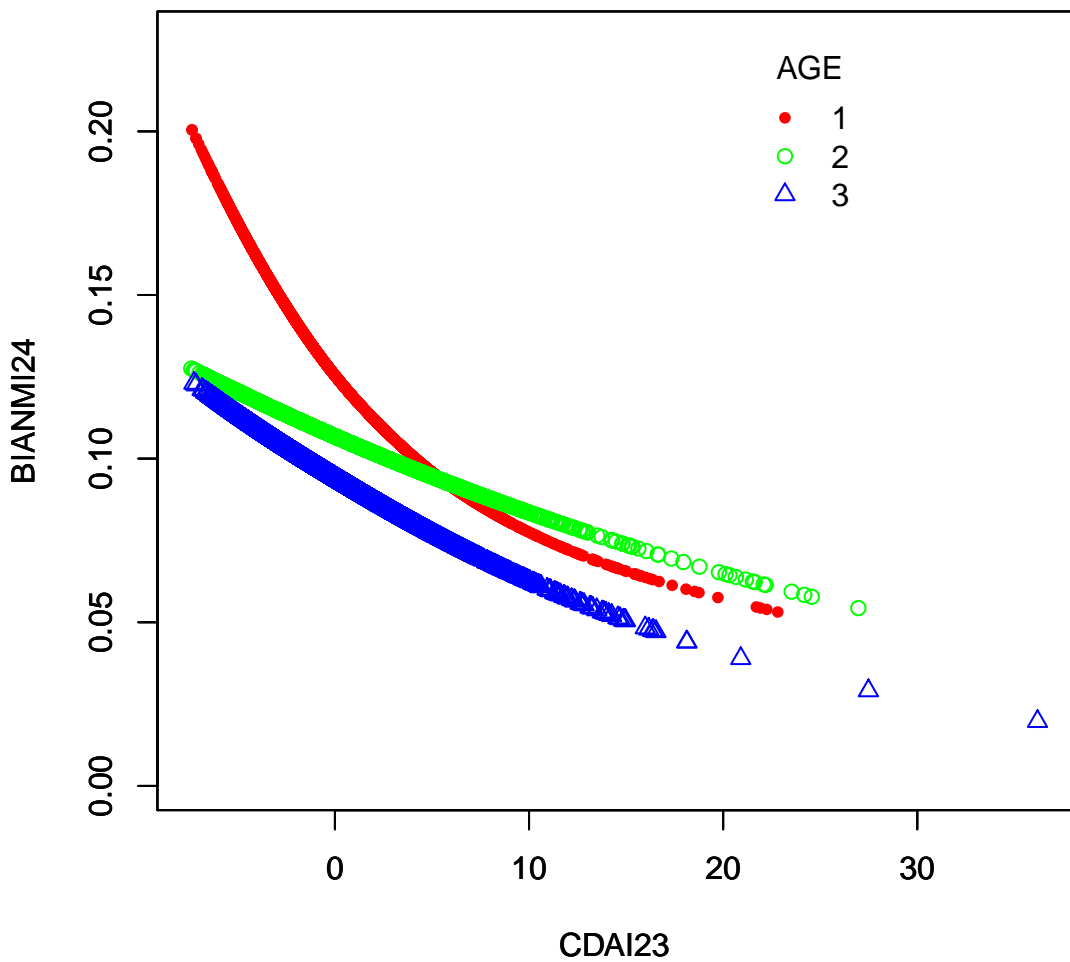

Supplement: S1 File — (ZIP) [file pone.0311168.s001.zip › CDAI/all/PROJ2_3_tbl1/PROJ2_3_tbl1_BIANMI24_CDAI23_smooth.pdf]

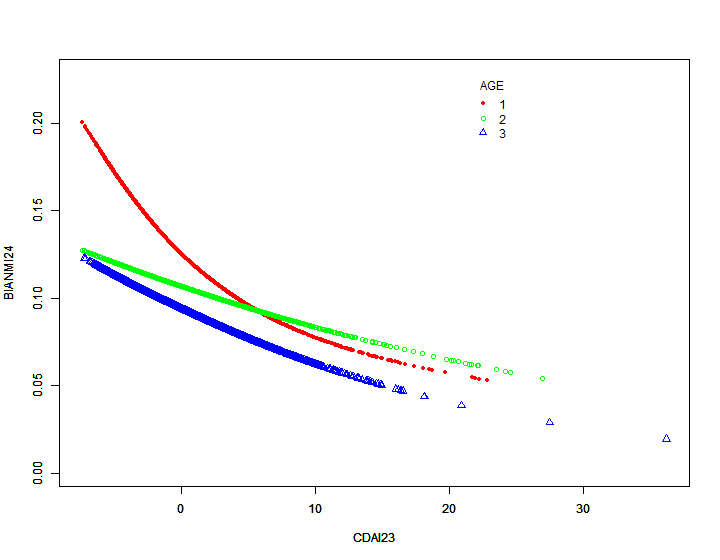

Supplement: S1 File — (ZIP) [file pone.0311168.s001.zip › CDAI/all/PROJ2_3_tbl1/PROJ2_3_tbl1_BIANMI24_CDAI23_smooth.png]

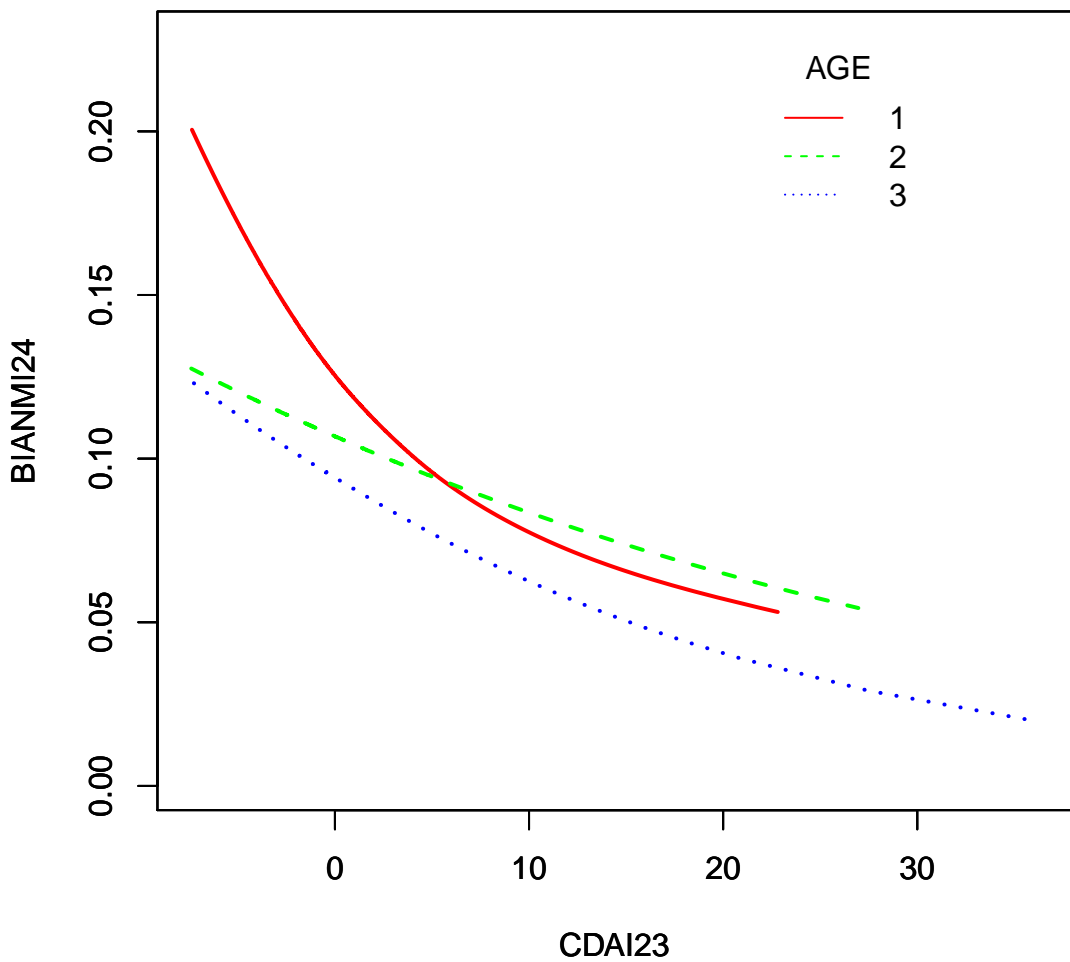

Supplement: S1 File — (ZIP) [file pone.0311168.s001.zip › CDAI/all/PROJ2_3_tbl1/PROJ2_3_tbl1_BIANMI24_CDAI23_smooth1.pdf]

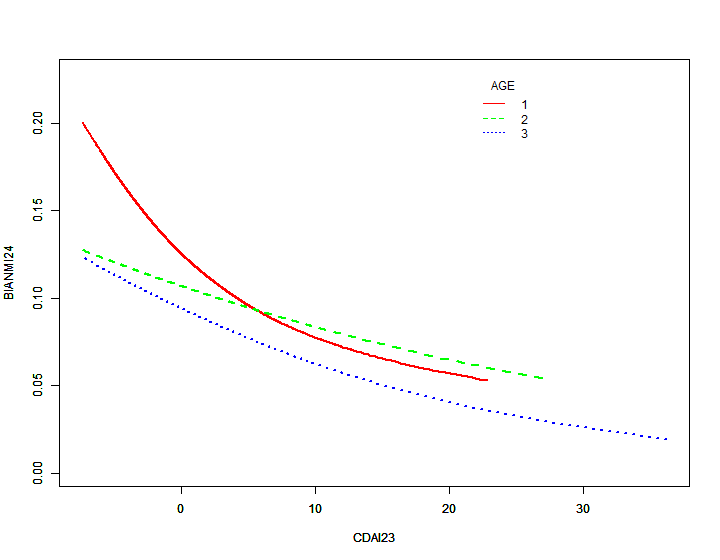

Supplement: S1 File — (ZIP) [file pone.0311168.s001.zip › CDAI/all/PROJ2_3_tbl1/PROJ2_3_tbl1_BIANMI24_CDAI23_smooth1.png]

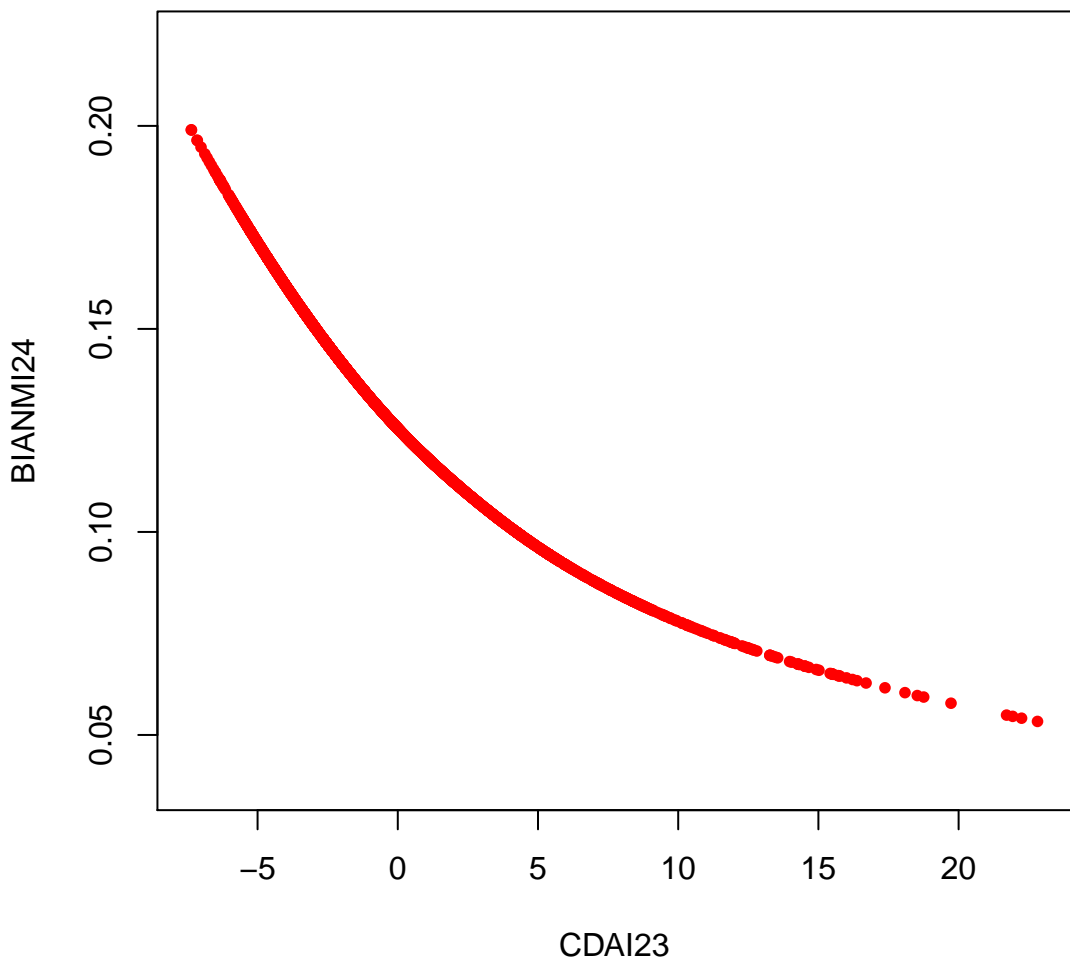

Supplement: S1 File — (ZIP) [file pone.0311168.s001.zip › CDAI/all/PROJ2_3_tbl/PROJ2_3_tbl_BIANMI24_CDAI23_AGE_1_smooth.pdf]

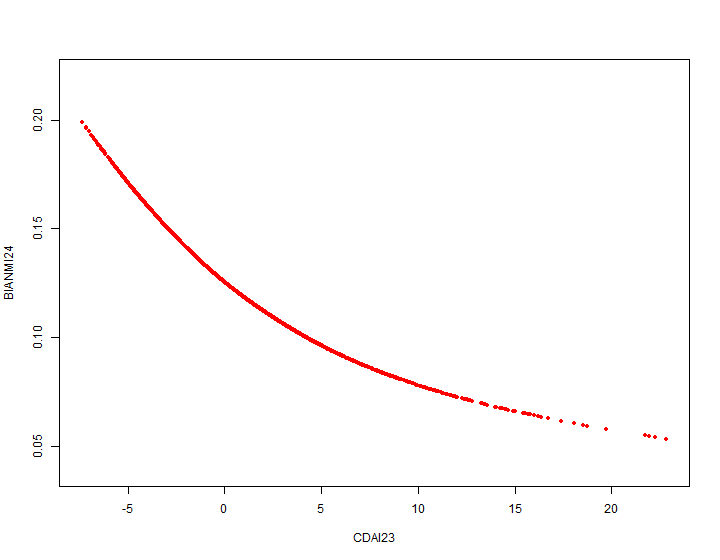

Supplement: S1 File — (ZIP) [file pone.0311168.s001.zip › CDAI/all/PROJ2_3_tbl/PROJ2_3_tbl_BIANMI24_CDAI23_AGE_1_smooth.png]

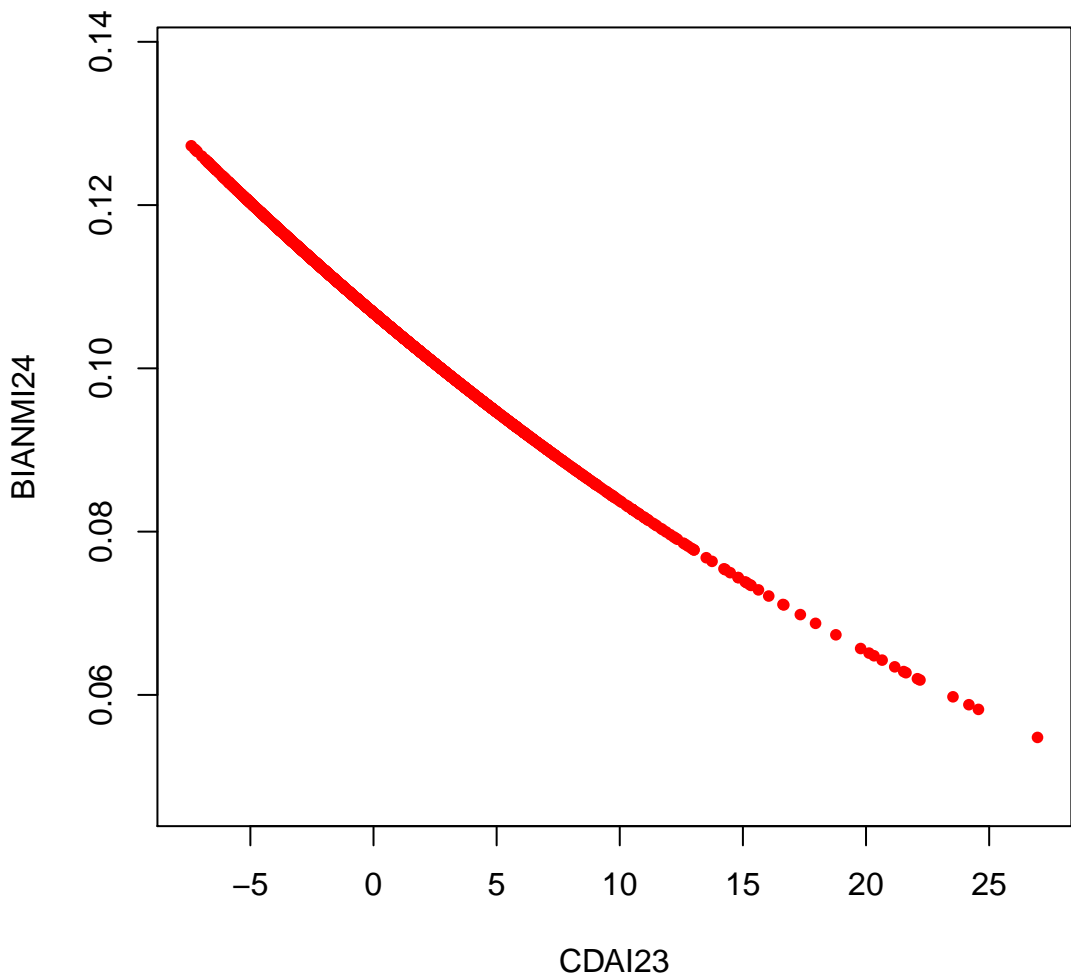

Supplement: S1 File — (ZIP) [file pone.0311168.s001.zip › CDAI/all/PROJ2_3_tbl/PROJ2_3_tbl_BIANMI24_CDAI23_AGE_2_smooth.pdf]

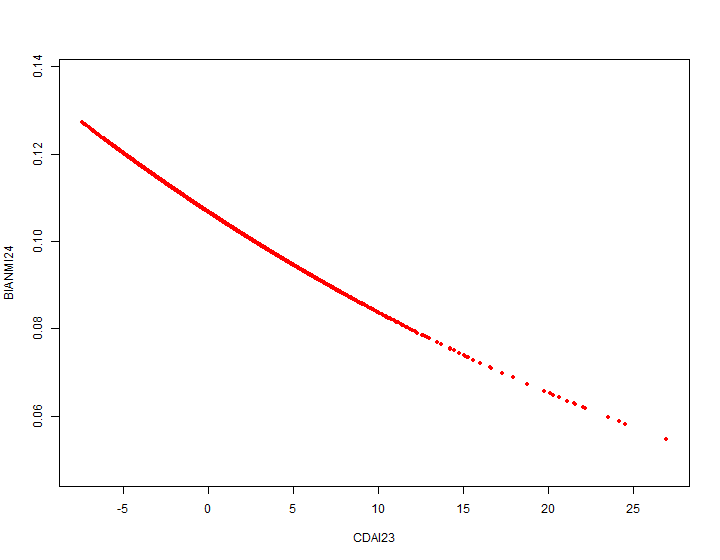

Supplement: S1 File — (ZIP) [file pone.0311168.s001.zip › CDAI/all/PROJ2_3_tbl/PROJ2_3_tbl_BIANMI24_CDAI23_AGE_2_smooth.png]

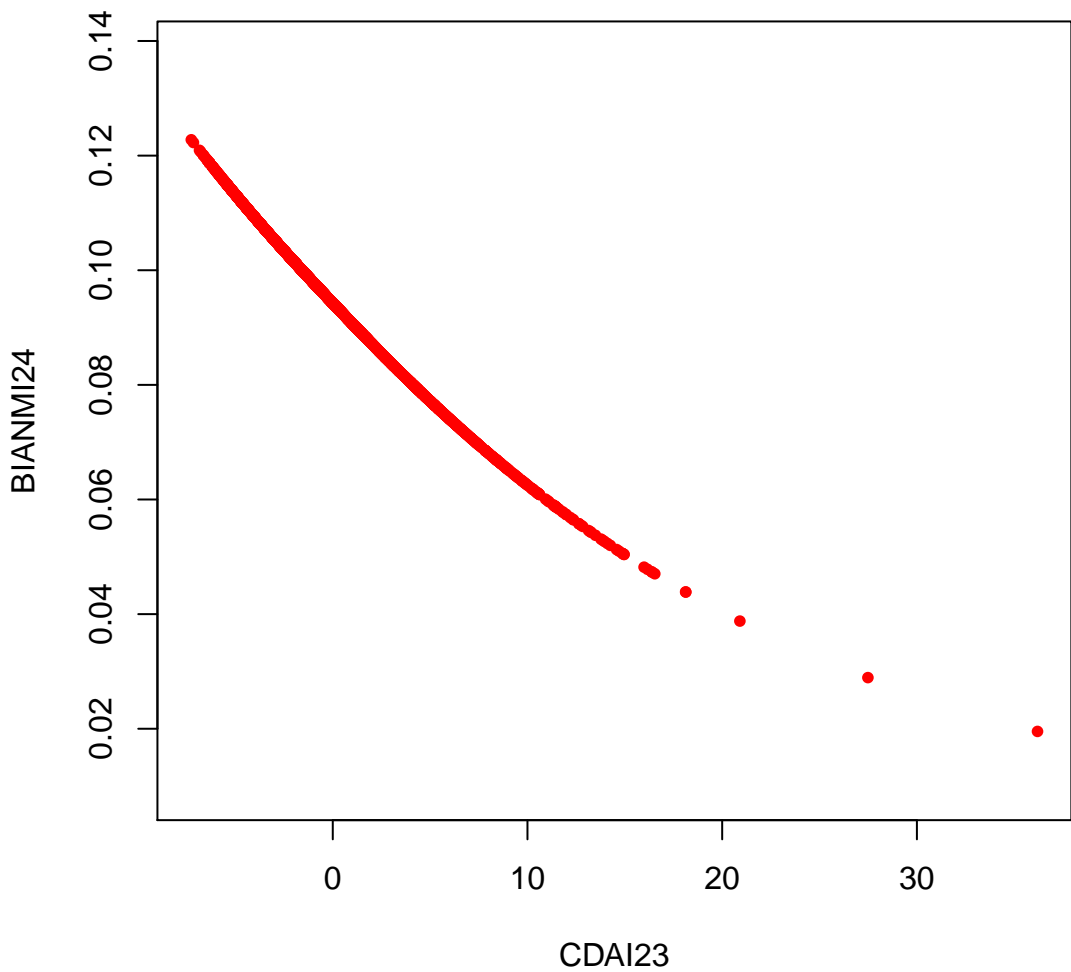

Supplement: S1 File — (ZIP) [file pone.0311168.s001.zip › CDAI/all/PROJ2_3_tbl/PROJ2_3_tbl_BIANMI24_CDAI23_AGE_3_smooth.pdf]

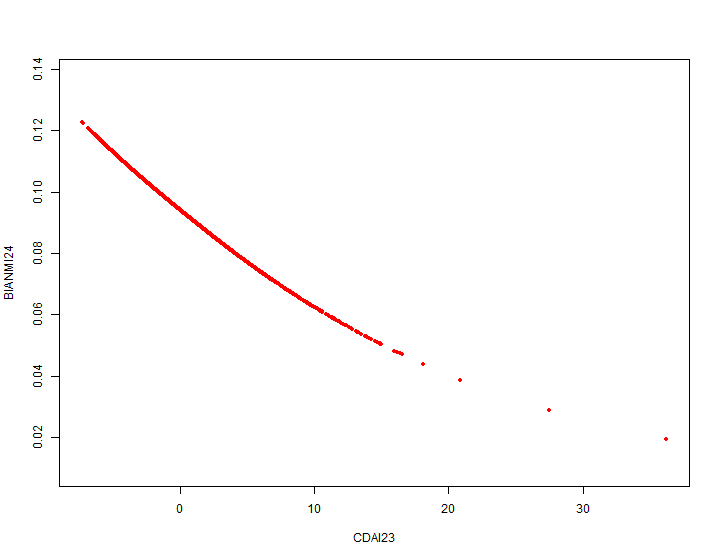

Supplement: S1 File — (ZIP) [file pone.0311168.s001.zip › CDAI/all/PROJ2_3_tbl/PROJ2_3_tbl_BIANMI24_CDAI23_AGE_3_smooth.png]

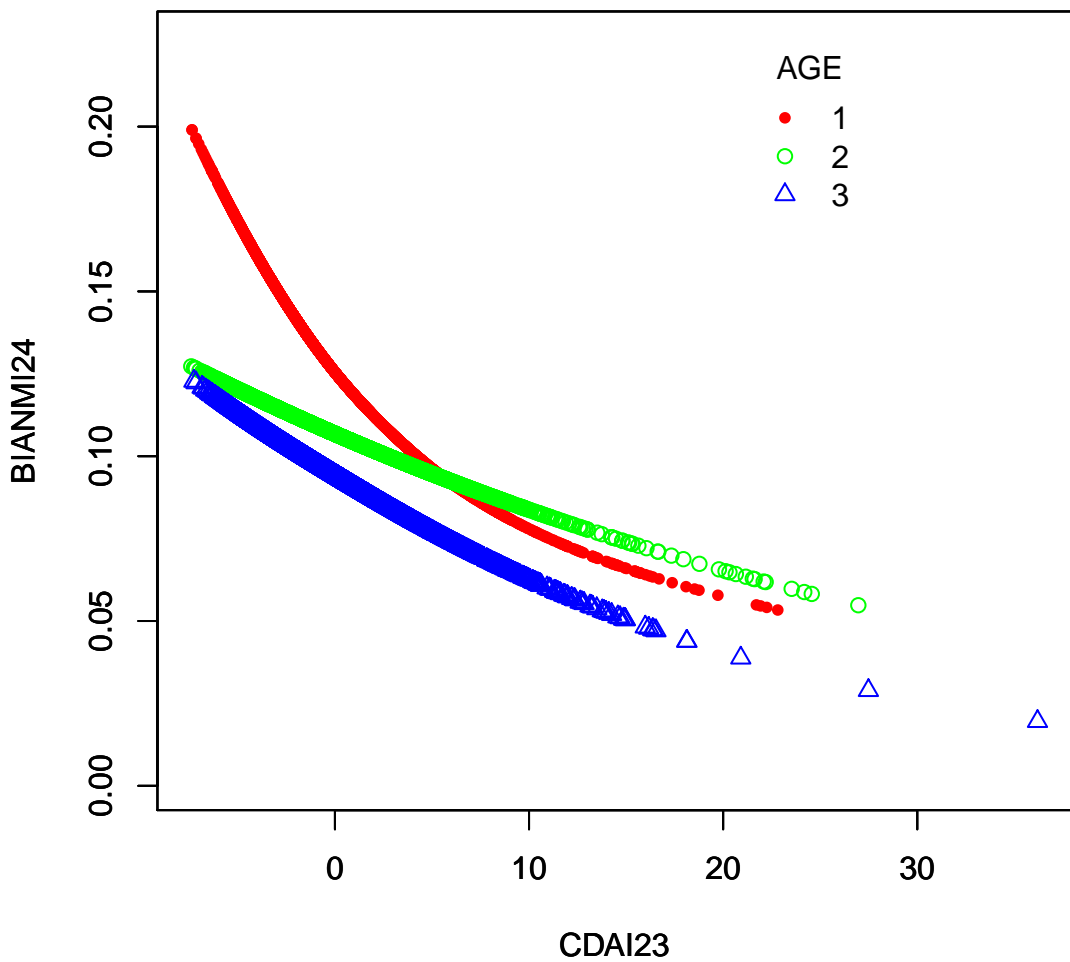

Supplement: S1 File — (ZIP) [file pone.0311168.s001.zip › CDAI/all/PROJ2_3_tbl/PROJ2_3_tbl_BIANMI24_CDAI23_smooth.pdf]

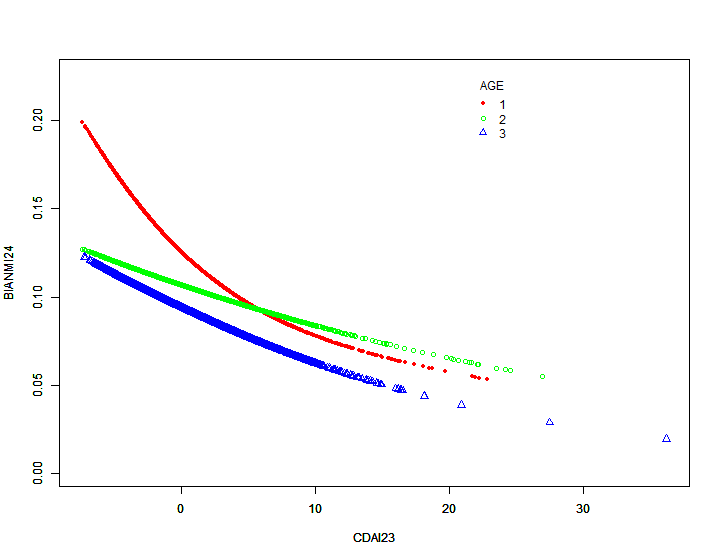

Supplement: S1 File — (ZIP) [file pone.0311168.s001.zip › CDAI/all/PROJ2_3_tbl/PROJ2_3_tbl_BIANMI24_CDAI23_smooth.png]

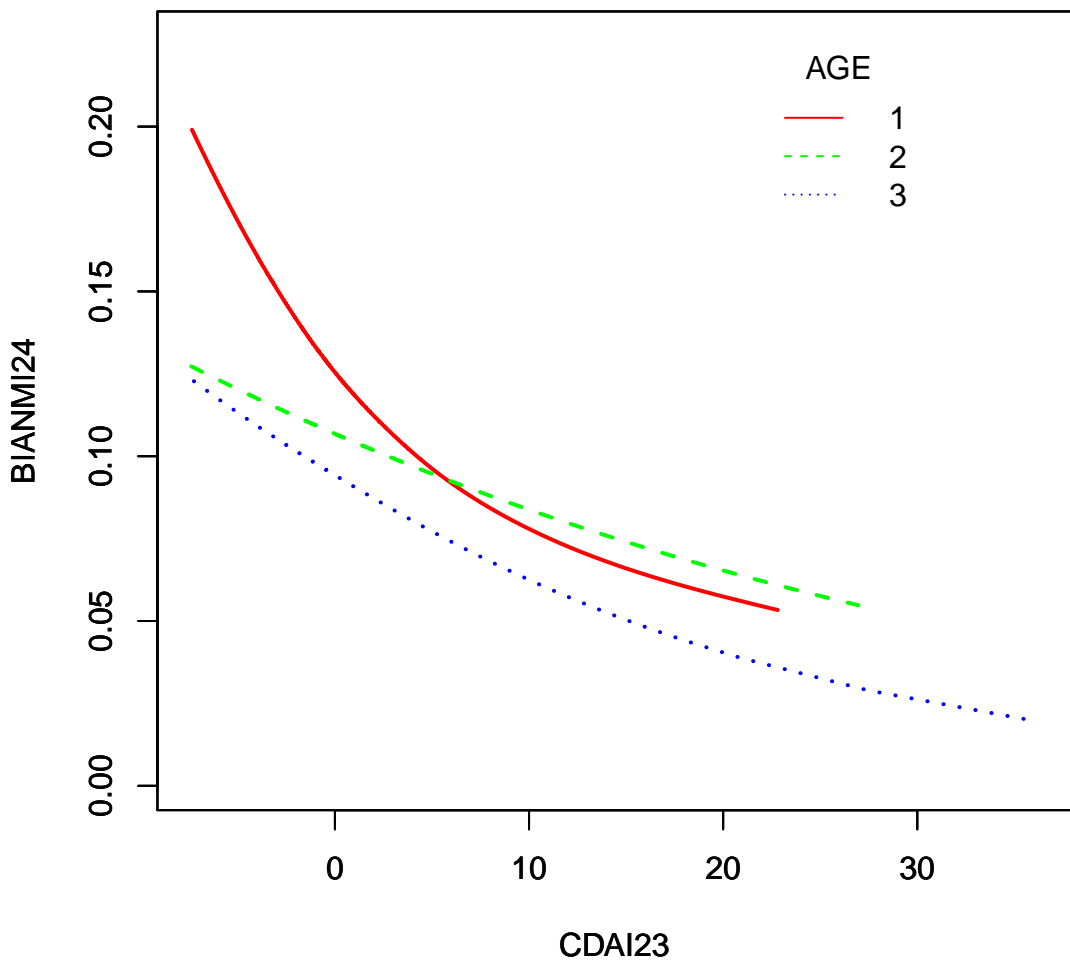

Supplement: S1 File — (ZIP) [file pone.0311168.s001.zip › CDAI/all/PROJ2_3_tbl/PROJ2_3_tbl_BIANMI24_CDAI23_smooth1.pdf]

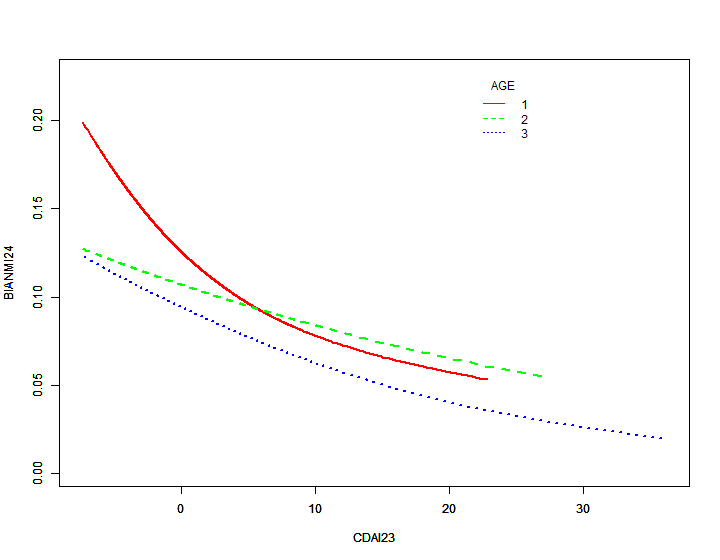

Supplement: S1 File — (ZIP) [file pone.0311168.s001.zip › CDAI/all/PROJ2_3_tbl/PROJ2_3_tbl_BIANMI24_CDAI23_smooth1.png]

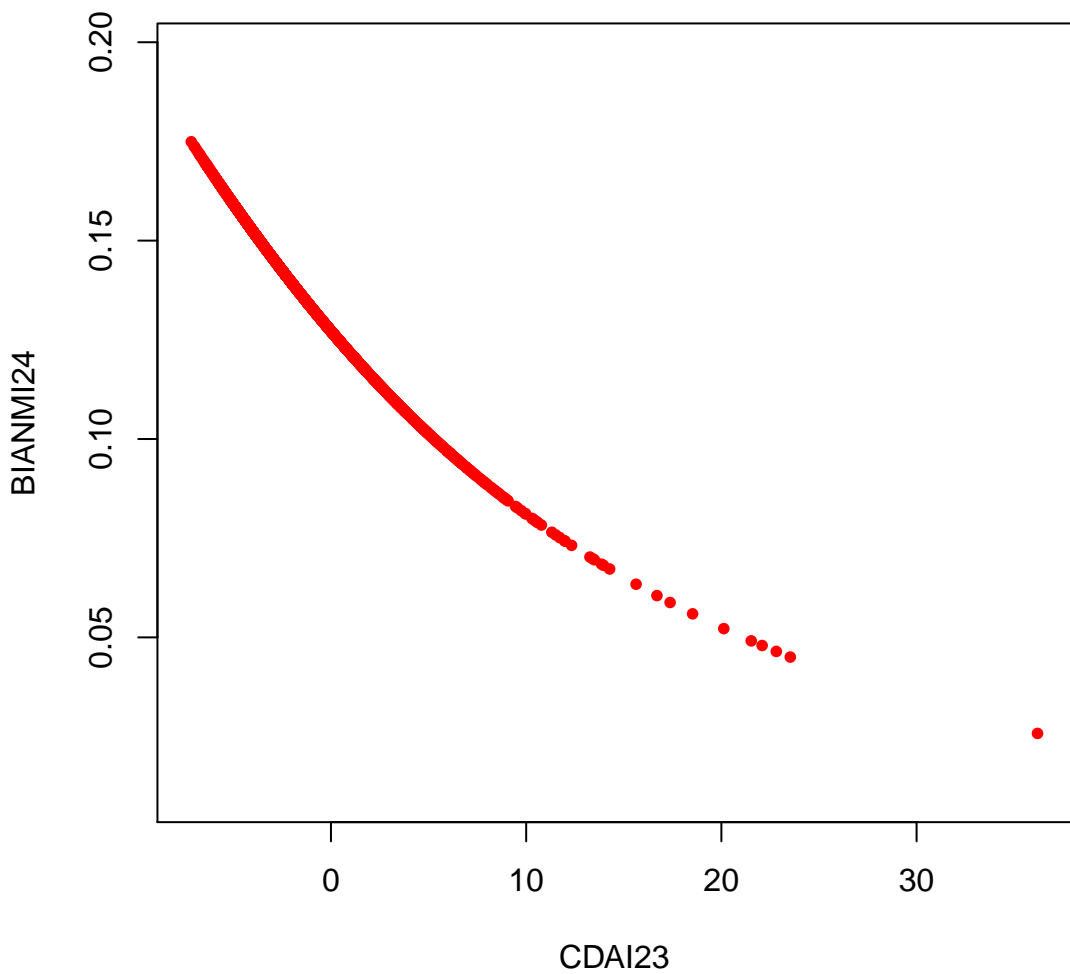

Supplement: S1 File — (ZIP) [file pone.0311168.s001.zip › CDAI/all/PROJ2_4_tbl/PROJ2_4_tbl_BIANMI24_CDAI23_JIAOYU4_1_smooth.pdf]

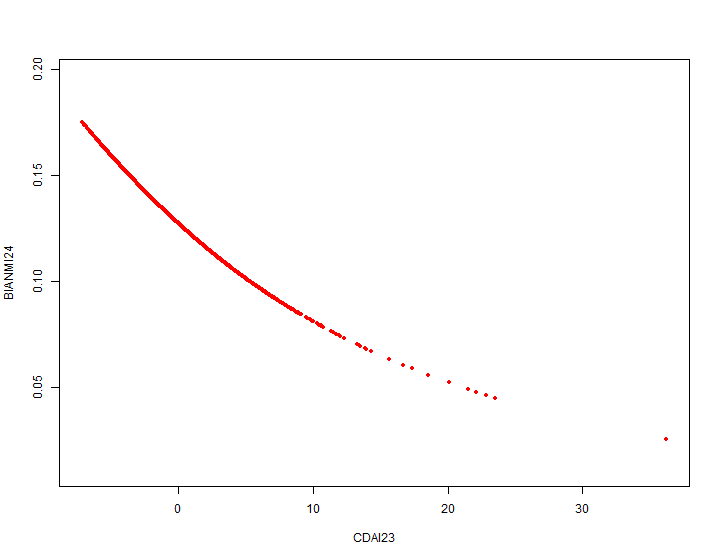

Supplement: S1 File — (ZIP) [file pone.0311168.s001.zip › CDAI/all/PROJ2_4_tbl/PROJ2_4_tbl_BIANMI24_CDAI23_JIAOYU4_1_smooth.png]

BIANMI24

0.15

0.10

0.05

-5

0

5

10

15

20

25

CDAI23

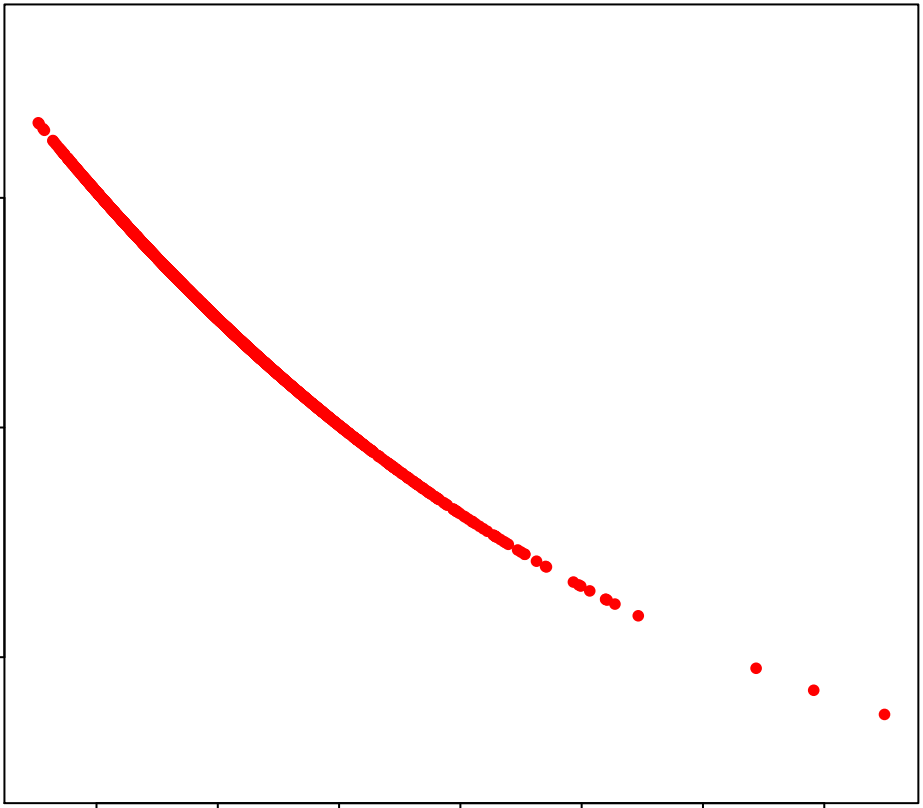

Supplement: S1 File — (ZIP) [file pone.0311168.s001.zip › CDAI/all/PROJ2_4_tbl/PROJ2_4_tbl_BIANMI24_CDAI23_JIAOYU4_2_smooth.pdf]

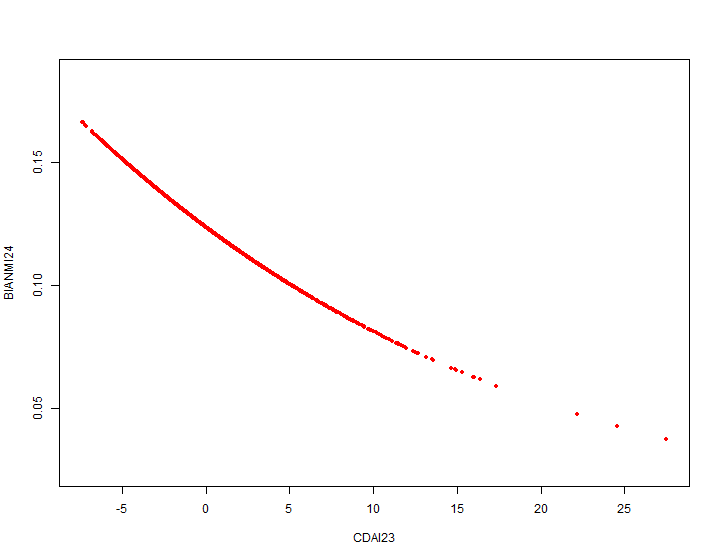

Supplement: S1 File — (ZIP) [file pone.0311168.s001.zip › CDAI/all/PROJ2_4_tbl/PROJ2_4_tbl_BIANMI24_CDAI23_JIAOYU4_2_smooth.png]

BIANMI24

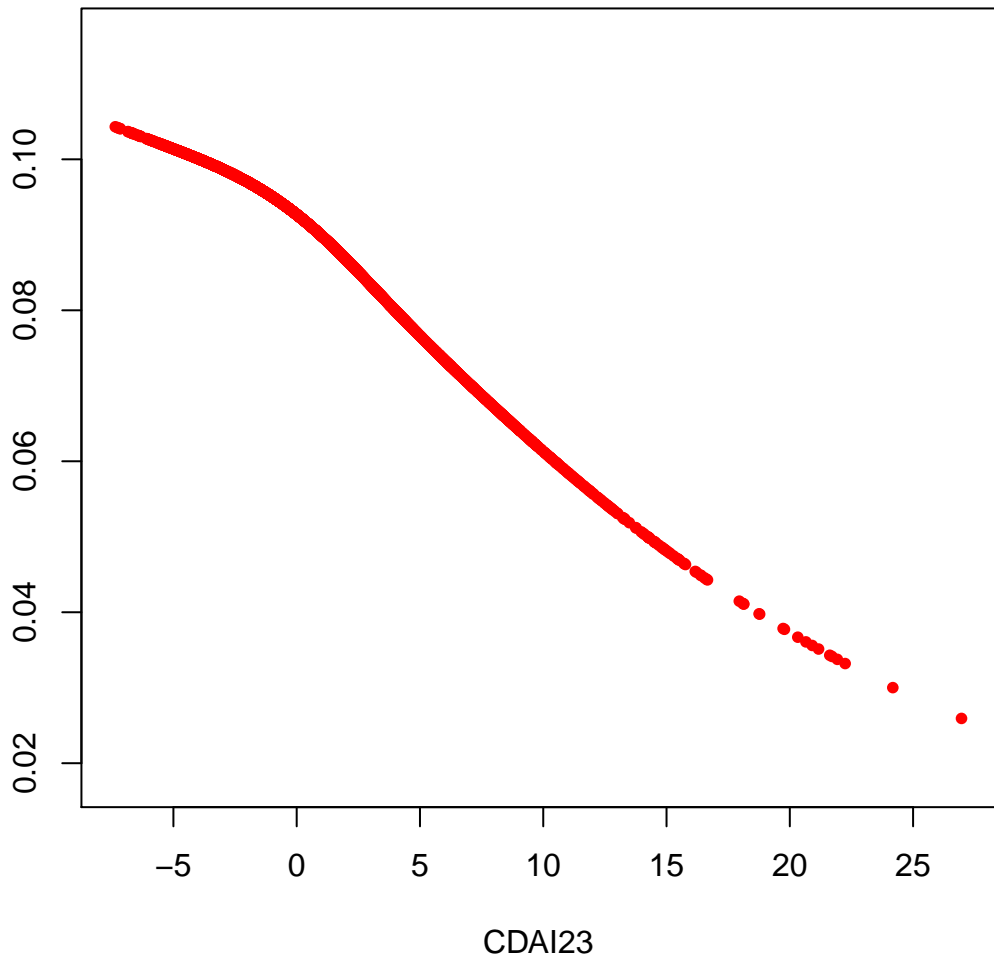

Supplement: S1 File — (ZIP) [file pone.0311168.s001.zip › CDAI/all/PROJ2_4_tbl/PROJ2_4_tbl_BIANMI24_CDAI23_JIAOYU4_3_smooth.pdf]

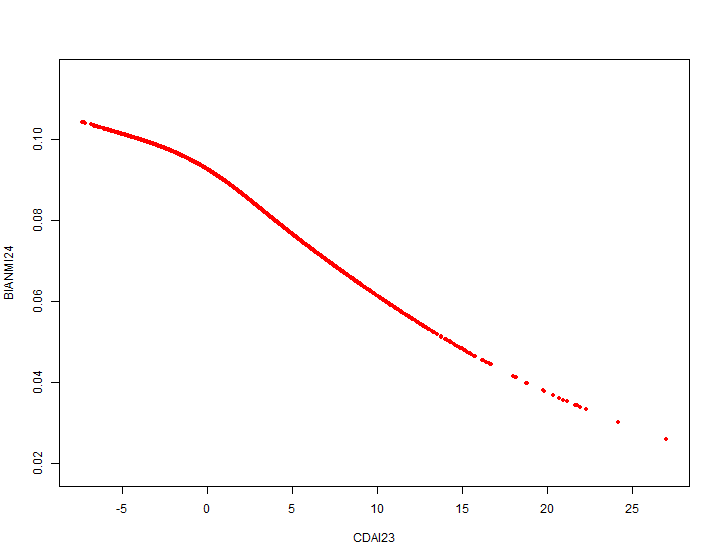

Supplement: S1 File — (ZIP) [file pone.0311168.s001.zip › CDAI/all/PROJ2_4_tbl/PROJ2_4_tbl_BIANMI24_CDAI23_JIAOYU4_3_smooth.png]

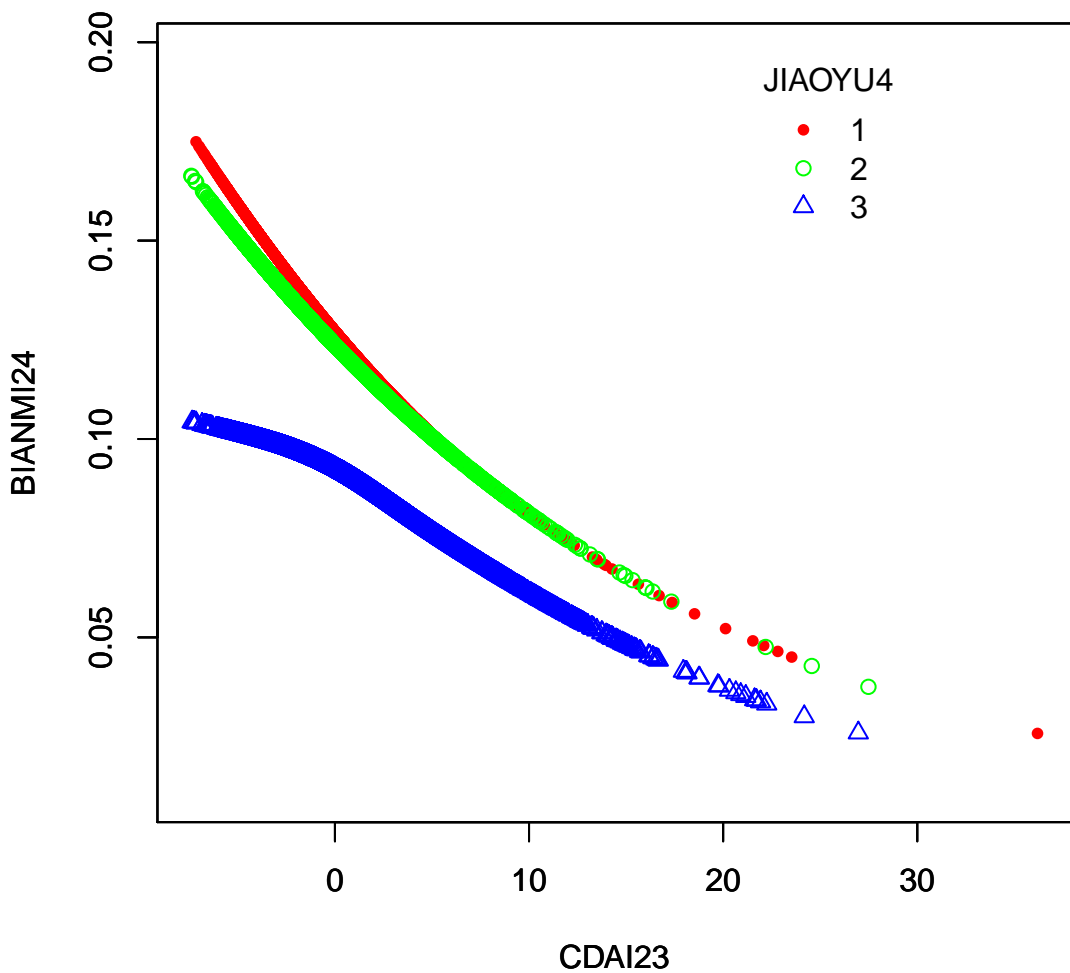

Supplement: S1 File — (ZIP) [file pone.0311168.s001.zip › CDAI/all/PROJ2_4_tbl/PROJ2_4_tbl_BIANMI24_CDAI23_smooth.pdf]

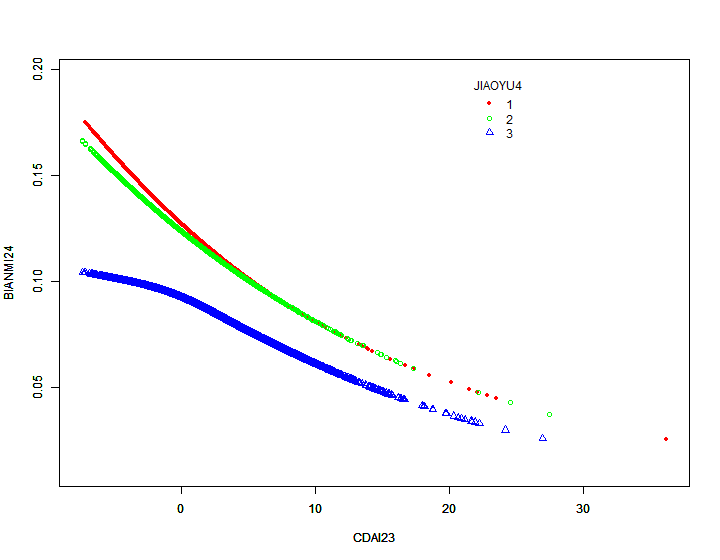

Supplement: S1 File — (ZIP) [file pone.0311168.s001.zip › CDAI/all/PROJ2_4_tbl/PROJ2_4_tbl_BIANMI24_CDAI23_smooth.png]

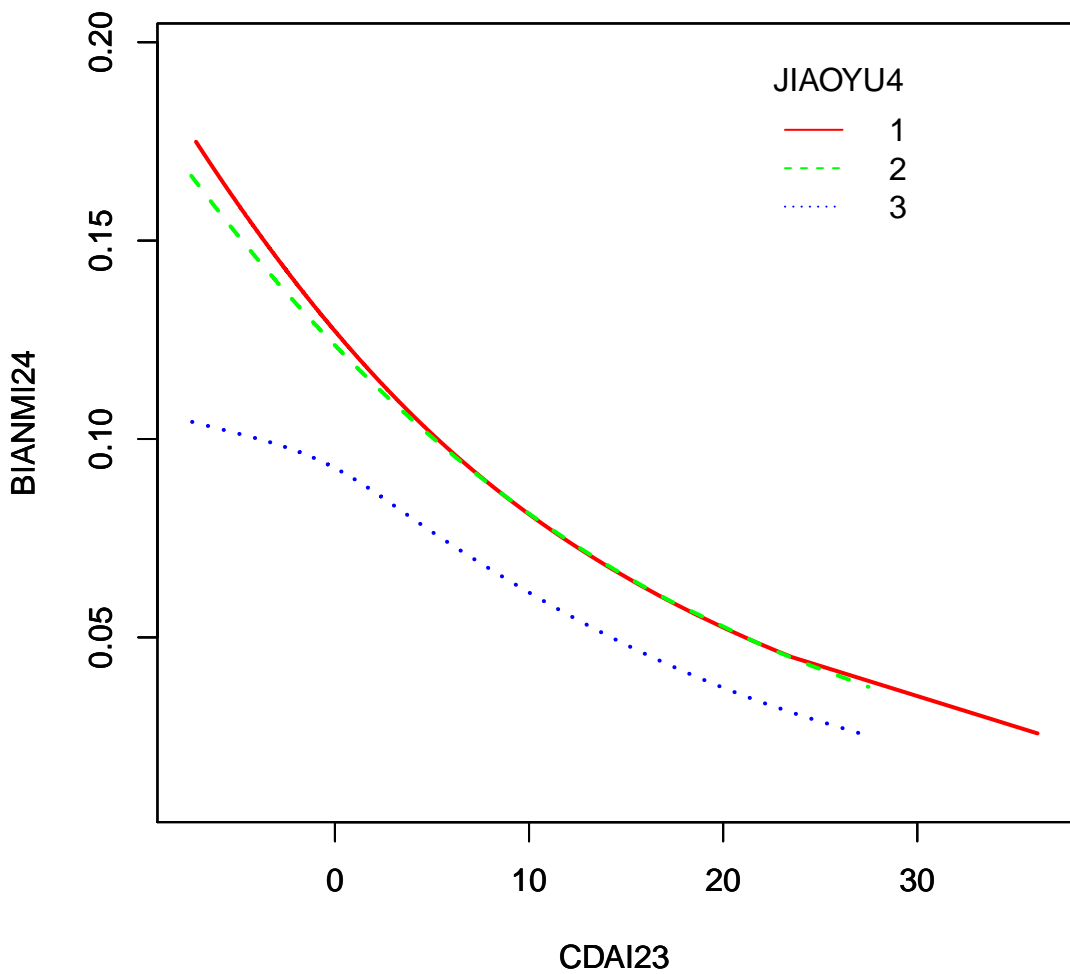

Supplement: S1 File — (ZIP) [file pone.0311168.s001.zip › CDAI/all/PROJ2_4_tbl/PROJ2_4_tbl_BIANMI24_CDAI23_smooth1.pdf]

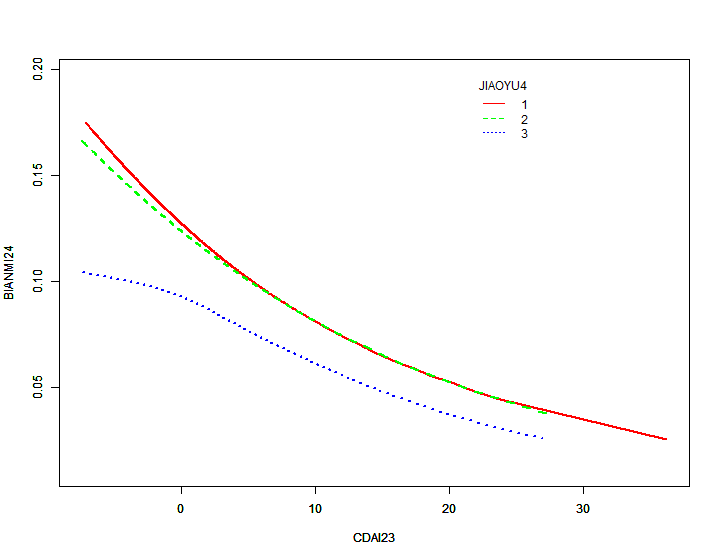

Supplement: S1 File — (ZIP) [file pone.0311168.s001.zip › CDAI/all/PROJ2_4_tbl/PROJ2_4_tbl_BIANMI24_CDAI23_smooth1.png]

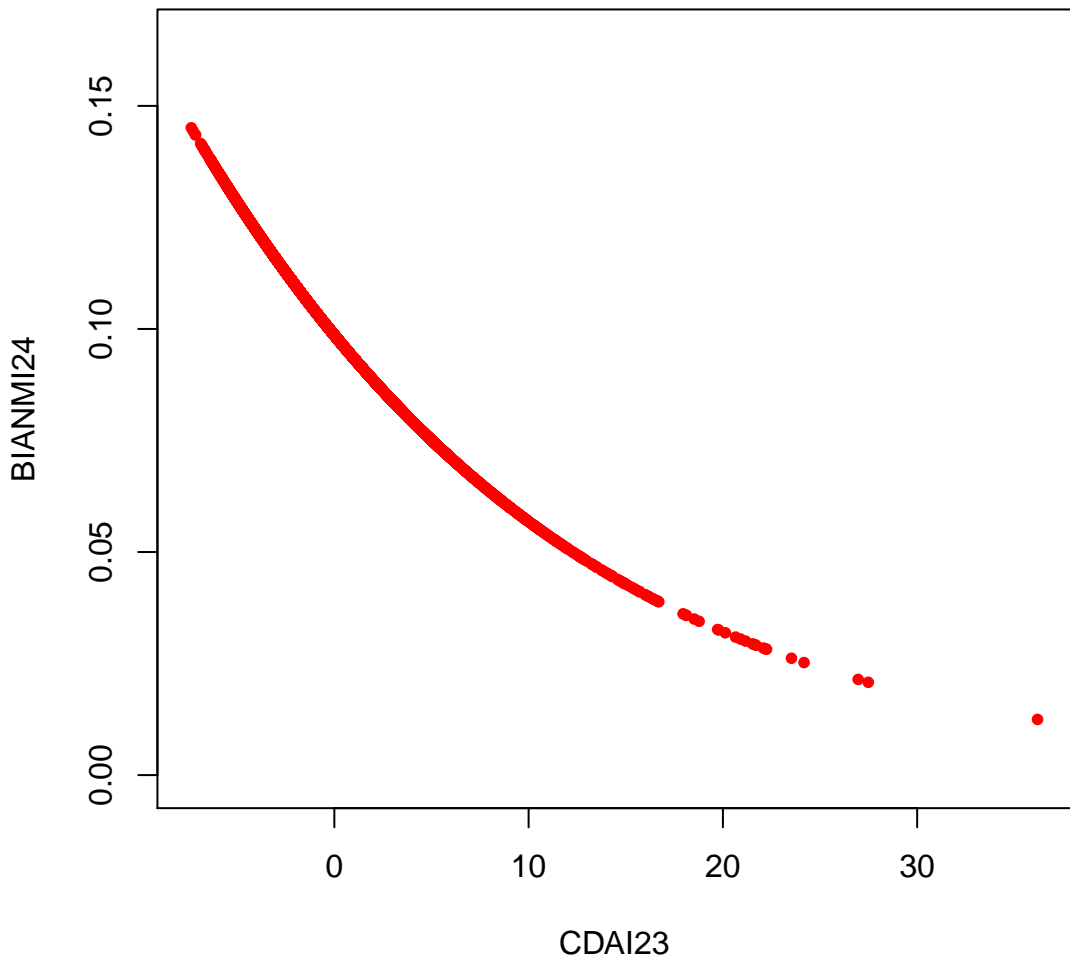

Supplement: S1 File — (ZIP) [file pone.0311168.s001.zip › CDAI/all/PROJ2_5_tbl/PROJ2_5_tbl_BIANMI24_CDAI23_HUNYING5_1_smooth.pdf]

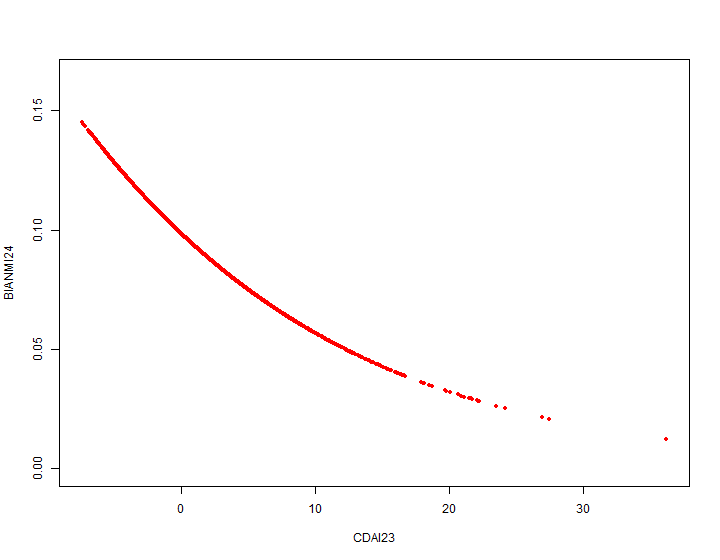

Supplement: S1 File — (ZIP) [file pone.0311168.s001.zip › CDAI/all/PROJ2_5_tbl/PROJ2_5_tbl_BIANMI24_CDAI23_HUNYING5_1_smooth.png]

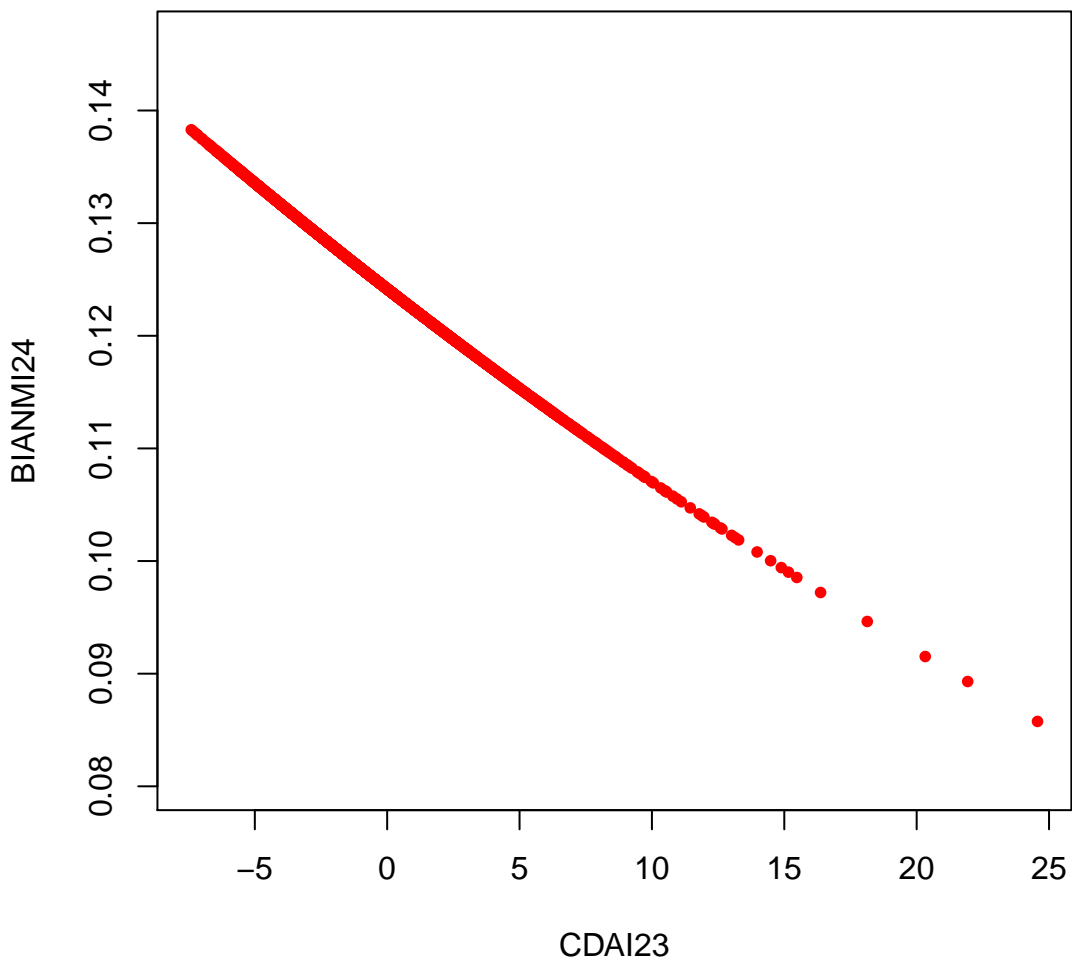

Supplement: S1 File — (ZIP) [file pone.0311168.s001.zip › CDAI/all/PROJ2_5_tbl/PROJ2_5_tbl_BIANMI24_CDAI23_HUNYING5_2_smooth.pdf]

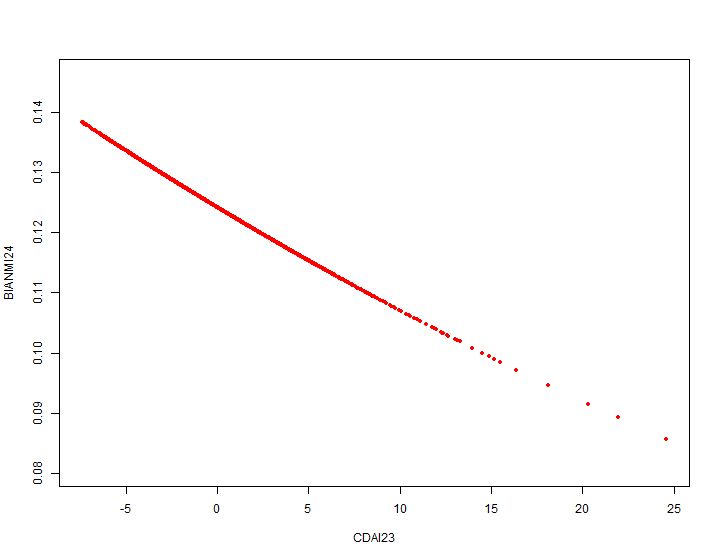

Supplement: S1 File — (ZIP) [file pone.0311168.s001.zip › CDAI/all/PROJ2_5_tbl/PROJ2_5_tbl_BIANMI24_CDAI23_HUNYING5_2_smooth.png]

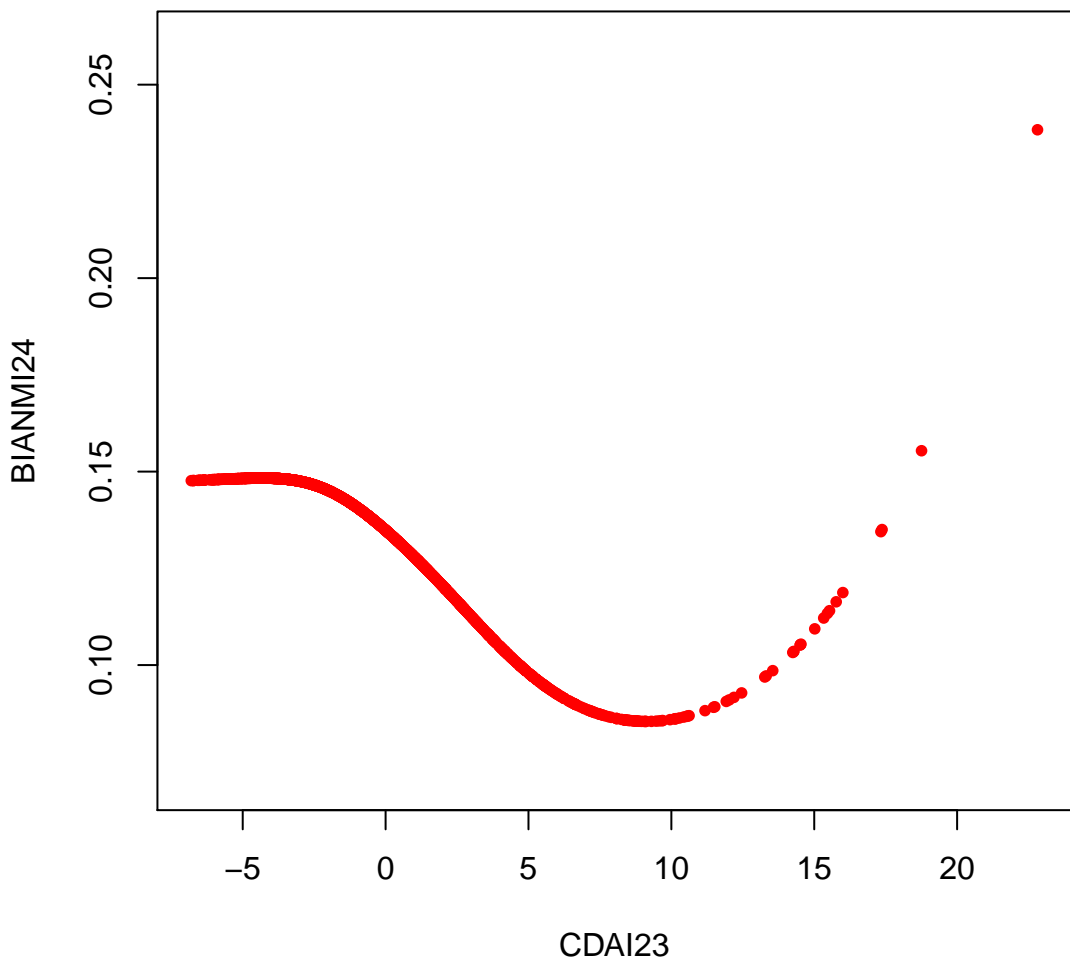

Supplement: S1 File — (ZIP) [file pone.0311168.s001.zip › CDAI/all/PROJ2_5_tbl/PROJ2_5_tbl_BIANMI24_CDAI23_HUNYING5_3_smooth.pdf]

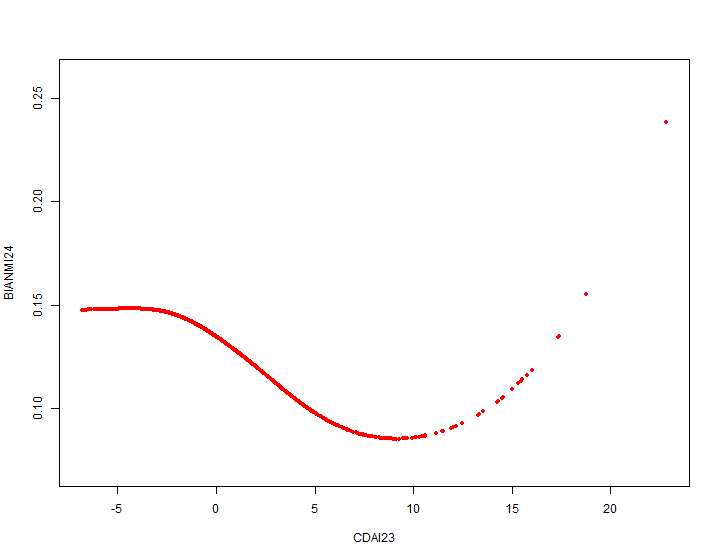

Supplement: S1 File — (ZIP) [file pone.0311168.s001.zip › CDAI/all/PROJ2_5_tbl/PROJ2_5_tbl_BIANMI24_CDAI23_HUNYING5_3_smooth.png]

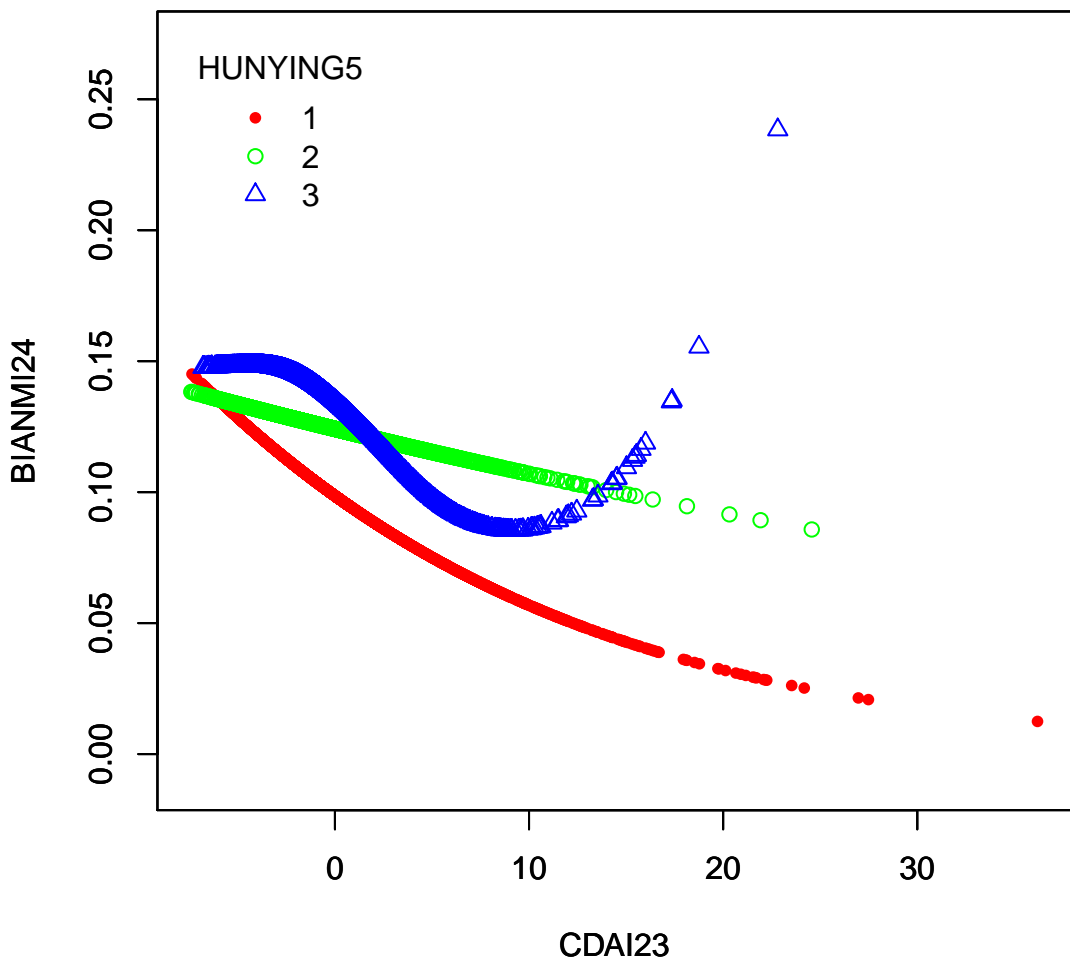

Supplement: S1 File — (ZIP) [file pone.0311168.s001.zip › CDAI/all/PROJ2_5_tbl/PROJ2_5_tbl_BIANMI24_CDAI23_smooth.pdf]

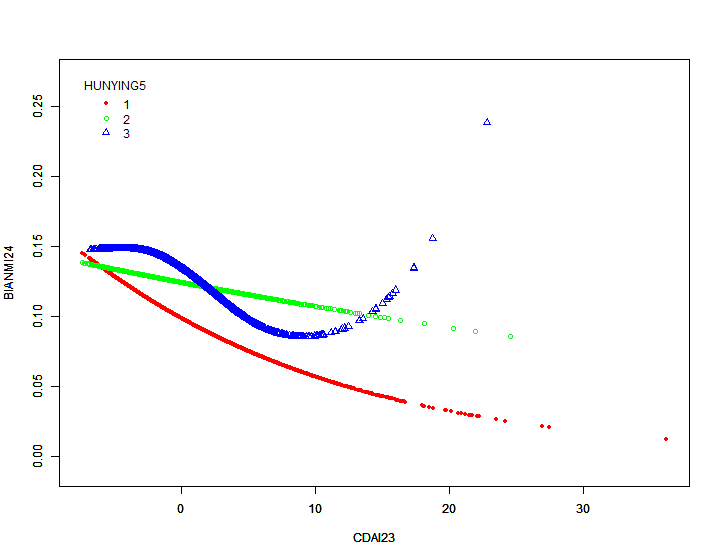

Supplement: S1 File — (ZIP) [file pone.0311168.s001.zip › CDAI/all/PROJ2_5_tbl/PROJ2_5_tbl_BIANMI24_CDAI23_smooth.png]

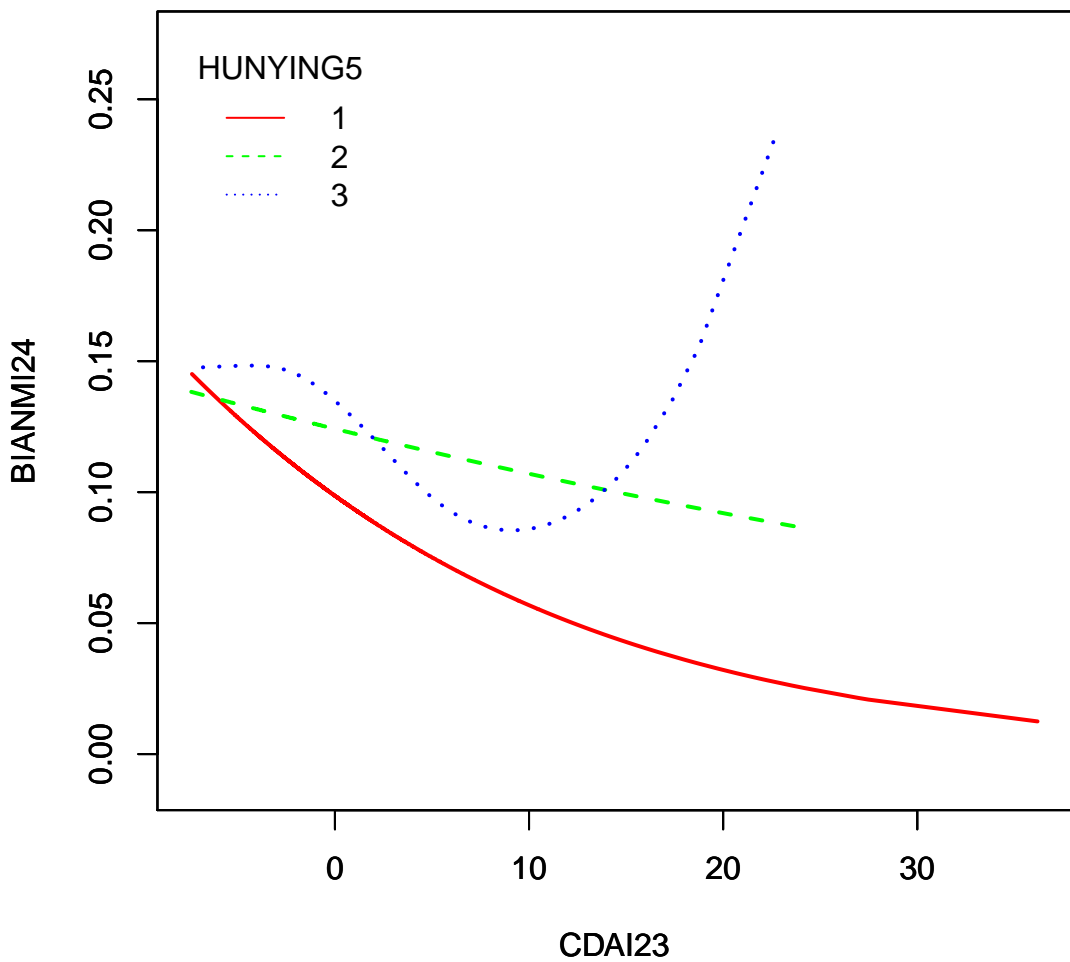

Supplement: S1 File — (ZIP) [file pone.0311168.s001.zip › CDAI/all/PROJ2_5_tbl/PROJ2_5_tbl_BIANMI24_CDAI23_smooth1.pdf]

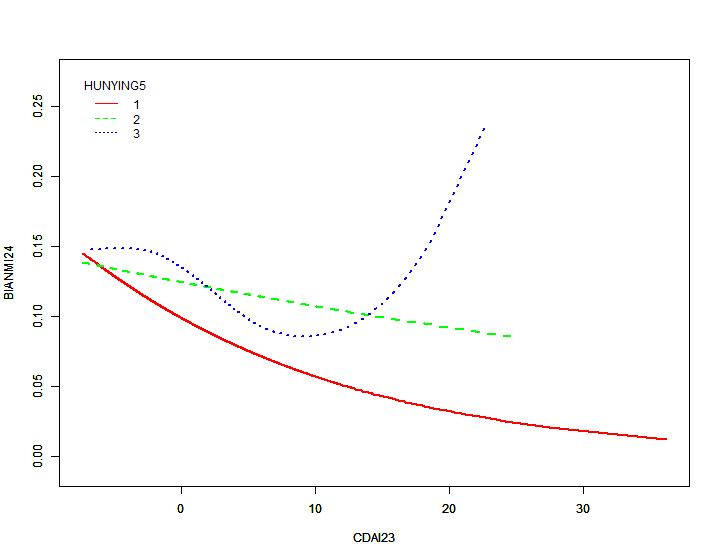

Supplement: S1 File — (ZIP) [file pone.0311168.s001.zip › CDAI/all/PROJ2_5_tbl/PROJ2_5_tbl_BIANMI24_CDAI23_smooth1.png]

BIANMI24

0.15

0.10

0.05

0

10

20

30

CDAI23

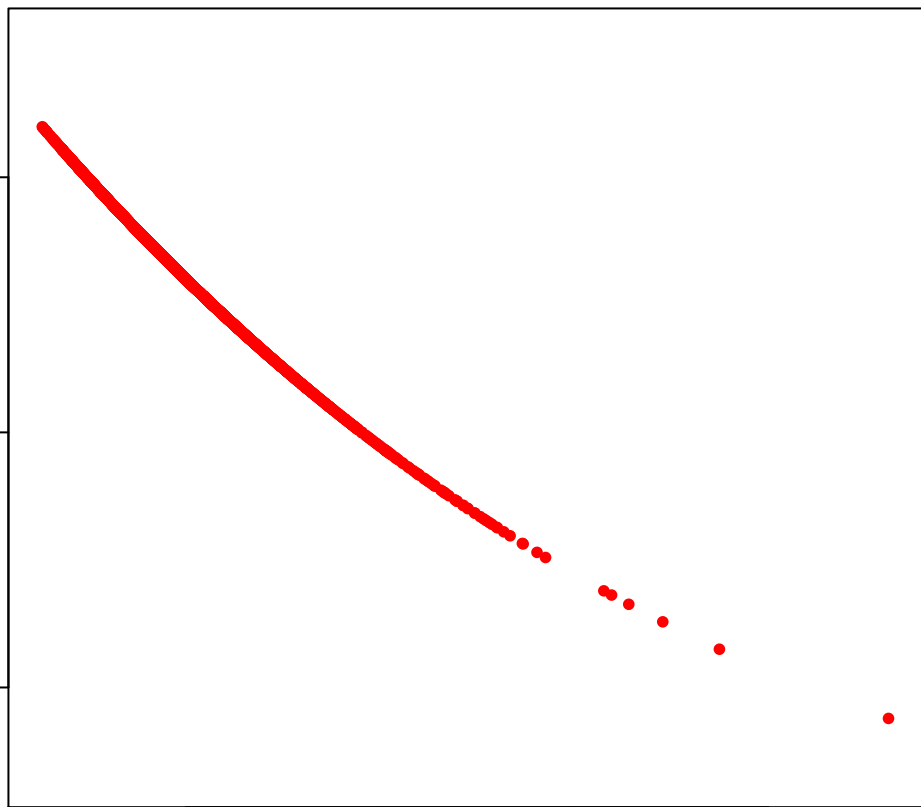

Supplement: S1 File — (ZIP) [file pone.0311168.s001.zip › CDAI/all/PROJ2_6_tbl/PROJ2_6_tbl_BIANMI24_CDAI23_PIR6_1_smooth.pdf]

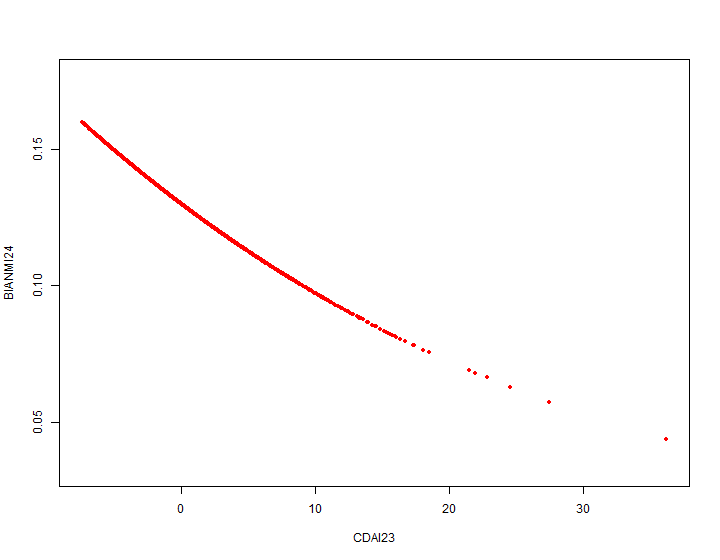

Supplement: S1 File — (ZIP) [file pone.0311168.s001.zip › CDAI/all/PROJ2_6_tbl/PROJ2_6_tbl_BIANMI24_CDAI23_PIR6_1_smooth.png]

BIANMI24

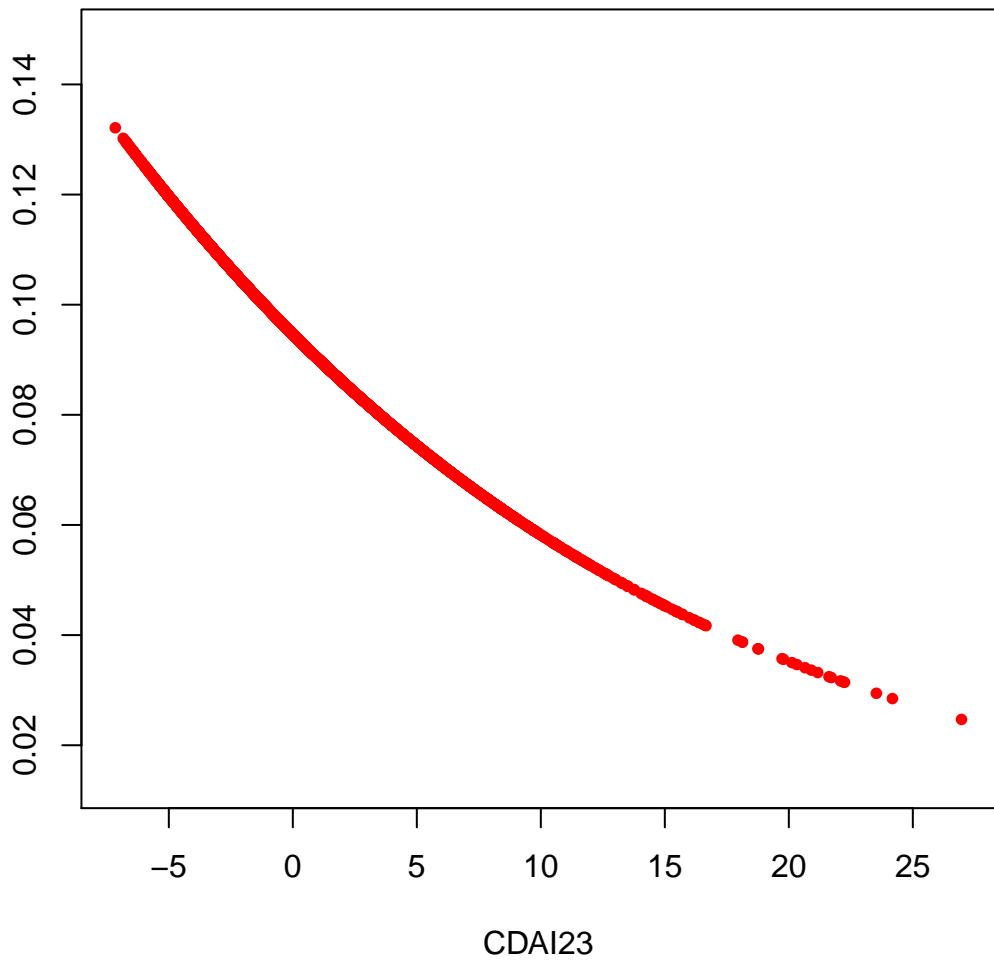

Supplement: S1 File — (ZIP) [file pone.0311168.s001.zip › CDAI/all/PROJ2_6_tbl/PROJ2_6_tbl_BIANMI24_CDAI23_PIR6_2_smooth.pdf]

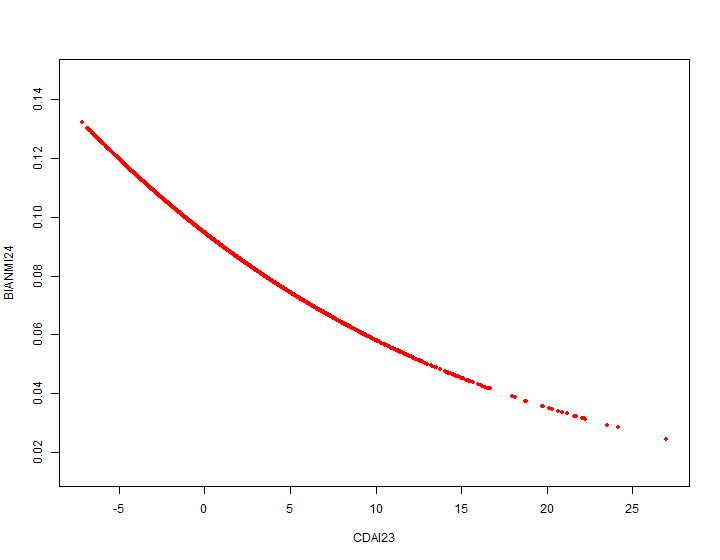

Supplement: S1 File — (ZIP) [file pone.0311168.s001.zip › CDAI/all/PROJ2_6_tbl/PROJ2_6_tbl_BIANMI24_CDAI23_PIR6_2_smooth.png]
