# Supplementary material for: Association between the composite dietary antioxidant index and constipation: Evidence from NHANES 2005–2010
Source: PLoS One. 2024 Sep 27;19(9):e0311168. doi: 10.1371/journal.pone.0311168 (PMC11432863; doi:10.1371/journal.pone.0311168)
Supplement: S1 File — (ZIP) [file pone.0311168.s001.zip › CDAI/all/PROJ2_7_tbl/PROJ2_7_tbl_BIANMI24_CDAI23_smooth.pdf]

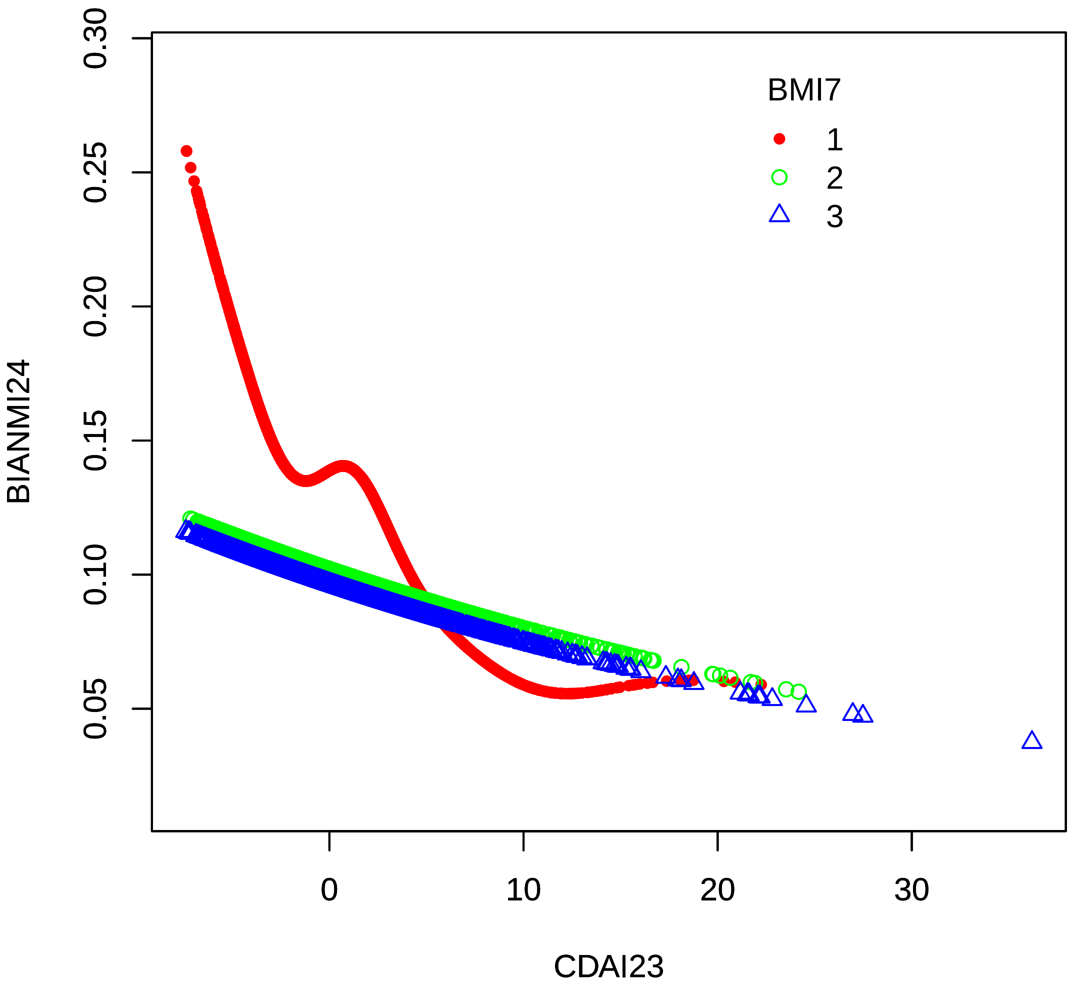
BMI7
1
2
3
0.05
0.10
0.15
0.20
0.25
0.30
BIANMI24
0
10
20
30
CDAI23
