# Supplementary material for: Association between the composite dietary antioxidant index and constipation: Evidence from NHANES 2005–2010
Source: PLoS One. 2024 Sep 27;19(9):e0311168. doi: 10.1371/journal.pone.0311168 (PMC11432863; doi:10.1371/journal.pone.0311168)
Supplement: S1 File — (ZIP) [file pone.0311168.s001.zip › CDAI/all/PROJ2_8_tbl/PROJ2_8_tbl.htm]

BIANMI24
vs.
CDAI23

Generalize additive models
Outcome: BIANMI24
Exposure: CDAI23
Linear terms effect

|  |  |  |  |  |  |  |  |
| --- | --- | --- | --- | --- | --- | --- | --- |
|  | Estimate | Std. Error | z value | Pr(>\|z\|) | exp(est) | 95%CI low | 95%CI upp |
| (Intercept) | -1.9921 | 0.6326 | -3.1492 | 0.0016 | 0.1364 | 0.0395 | 0.4713 |
| factor(YIYU8)2 | -0.598 | 0.1098 | -5.4454 | 0 | 0.5499 | 0.4434 | 0.682 |
| factor(ZHONGZU3)2 | 0.3125 | 0.1314 | 2.3777 | 0.0174 | 1.3669 | 1.0564 | 1.7686 |
| factor(ZHONGZU3)3 | 0.2272 | 0.1049 | 2.1655 | 0.0303 | 1.255 | 1.0218 | 1.5415 |
| factor(ZHONGZU3)4 | 0.5641 | 0.1126 | 5.0076 | 0 | 1.7579 | 1.4096 | 2.1922 |
| factor(ZHONGZU3)5 | 0.111 | 0.1939 | 0.5726 | 0.5669 | 1.1174 | 0.7641 | 1.634 |
| YUNDONG9 | -0.1185 | 0.1003 | -1.1815 | 0.2374 | 0.8882 | 0.7297 | 1.0812 |
| DRINK10 | 0.1099 | 0.0727 | 1.5113 | 0.1307 | 1.1162 | 0.9679 | 1.2873 |
| factor(XIYAN11)2 | -0.1476 | 0.1056 | -1.3976 | 0.1622 | 0.8628 | 0.7015 | 1.0612 |
| factor(XIYAN11)3 | 0.0949 | 0.0864 | 1.0981 | 0.2722 | 1.0996 | 0.9282 | 1.3025 |
| GAOXUEYA12 | 0.1845 | 0.0765 | 2.411 | 0.0159 | 1.2027 | 1.0351 | 1.3973 |
| TANGNIAOBING13 | -0.0118 | 0.1013 | -0.1163 | 0.9074 | 0.9883 | 0.8104 | 1.2053 |
| FEIBING14 | -0.1039 | 0.086 | -1.2079 | 0.2271 | 0.9013 | 0.7614 | 1.0668 |
| XINGZHANGBING15 | -0.3301 | 0.1192 | -2.7697 | 0.0056 | 0.7188 | 0.5691 | 0.908 |
| GANBING16 | 0.226 | 0.1946 | 1.1618 | 0.2453 | 1.2536 | 0.8562 | 1.8356 |
| DANBAIZHI17 | 0.0046 | 0.0026 | 1.7608 | 0.0783 | 1.0046 | 0.9995 | 1.0097 |
| TANSHUI18 | 0.0064 | 0.0015 | 4.2159 | 0 | 1.0064 | 1.0034 | 1.0094 |
| XIANWEI19 | -0.0207 | 0.0065 | -3.1903 | 0.0014 | 0.9795 | 0.9672 | 0.9921 |
| ZHIFANG20 | 0.0059 | 0.0037 | 1.62 | 0.1052 | 1.006 | 0.9988 | 1.0132 |
| SHUIFEN21 | -1e-04 | 0 | -3.3425 | 8e-04 | 0.9999 | 0.9998 | 0.9999 |
| NENGLIANG22 | -0.001 | 4e-04 | -2.6899 | 0.0071 | 0.999 | 0.9983 | 0.9997 |
| XINBIE1 | 0.8944 | 0.0804 | 11.118 | 0 | 2.4458 | 2.089 | 2.8635 |
| AGE2 | -0.0062 | 0.0026 | -2.4119 | 0.0159 | 0.9938 | 0.9888 | 0.9988 |
| factor(JIAOYU4)2 | -0.0545 | 0.0882 | -0.618 | 0.5366 | 0.947 | 0.7967 | 1.1256 |
| factor(JIAOYU4)3 | -0.4016 | 0.0859 | -4.6767 | 0 | 0.6692 | 0.5656 | 0.7919 |
| factor(HUNYING5)2 | 0.0519 | 0.0823 | 0.6304 | 0.5284 | 1.0533 | 0.8963 | 1.2378 |
| factor(HUNYING5)3 | 0.0255 | 0.0933 | 0.2731 | 0.7847 | 1.0258 | 0.8543 | 1.2317 |
| PIR6 | -0.1383 | 0.0694 | -1.9932 | 0.0462 | 0.8708 | 0.7601 | 0.9977 |
| factor(BMI7)2 | -0.1782 | 0.0799 | -2.23 | 0.0257 | 0.8367 | 0.7154 | 0.9786 |
| factor(BMI7)3 | -0.4182 | 0.0828 | -5.0513 | 0 | 0.6582 | 0.5596 | 0.7742 |

Chi-square tests for linear terms

|  |  |  |  |
| --- | --- | --- | --- |
|  | df | Chi.sq | p-value |
| factor(YIYU8) | 1 | 29.6529 | 0 |
| factor(ZHONGZU3) | 4 | 29.7363 | 0 |
| YUNDONG9 | 1 | 1.396 | 0.2374 |
| DRINK10 | 1 | 2.2839 | 0.1307 |
| factor(XIYAN11) | 2 | 7.1689 | 0.0278 |
| GAOXUEYA12 | 1 | 5.8131 | 0.0159 |
| TANGNIAOBING13 | 1 | 0.0135 | 0.9074 |
| FEIBING14 | 1 | 1.459 | 0.2271 |
| XINGZHANGBING15 | 1 | 7.6715 | 0.0056 |
| GANBING16 | 1 | 1.3498 | 0.2453 |
| DANBAIZHI17 | 1 | 3.1003 | 0.0783 |
| TANSHUI18 | 1 | 17.7734 | 0 |
| XIANWEI19 | 1 | 10.1782 | 0.0014 |
| ZHIFANG20 | 1 | 2.6244 | 0.1052 |
| SHUIFEN21 | 1 | 11.1721 | 8e-04 |
| NENGLIANG22 | 1 | 7.2356 | 0.0071 |
| XINBIE1 | 1 | 123.6101 | 0 |
| AGE2 | 1 | 5.8171 | 0.0159 |
| factor(JIAOYU4) | 2 | 27.9998 | 0 |
| factor(HUNYING5) | 2 | 0.4263 | 0.808 |
| PIR6 | 1 | 3.9729 | 0.0462 |
| factor(BMI7) | 2 | 25.6711 | 0 |

Approximate significance of smooth terms

|  |  |  |  |  |
| --- | --- | --- | --- | --- |
|  | edf | Ref.df | Chi.sq | p-value |
| s(CDAI23):factor(YIYU8)1 | 1.5166 | 1.8965 | 3.7705 | 0.1018 |
| s(CDAI23):factor(YIYU8)2 | 1.0074 | 1.0147 | 7.0118 | 0.0086 |

Model statistics

|  |  |
| --- | --- |
| N: | 10904 |
| Adj. r-square: | 0.054 |
| Deviance explained: | 0.0789 |
| UBRE score (sp.criterion): | -0.3611 |
| Scale estimate: | 1 |
| family: | binomial |
| link function: | logit |
