# Supplementary material for: Association between the composite dietary antioxidant index and constipation: Evidence from NHANES 2005–2010
Source: PLoS One. 2024 Sep 27;19(9):e0311168. doi: 10.1371/journal.pone.0311168 (PMC11432863; doi:10.1371/journal.pone.0311168)
Supplement: S1 File — (ZIP) [file pone.0311168.s001.zip › CDAI/all/PROJ2_9_tbl/PROJ2_9_tbl.htm]

BIANMI24
vs.
CDAI23

Generalize additive models
Outcome: BIANMI24
Exposure: CDAI23
Linear terms effect

|  |  |  |  |  |  |  |  |
| --- | --- | --- | --- | --- | --- | --- | --- |
|  | Estimate | Std. Error | z value | Pr(>\|z\|) | exp(est) | 95%CI low | 95%CI upp |
| (Intercept) | -1.4952 | 0.6214 | -2.4063 | 0.0161 | 0.2242 | 0.0663 | 0.7578 |
| factor(YUNDONG9)2 | -0.0879 | 0.1035 | -0.8496 | 0.3956 | 0.9158 | 0.7477 | 1.1218 |
| factor(ZHONGZU3)2 | 0.3079 | 0.1314 | 2.3427 | 0.0191 | 1.3605 | 1.0516 | 1.7602 |
| factor(ZHONGZU3)3 | 0.224 | 0.1049 | 2.1354 | 0.0327 | 1.2511 | 1.0186 | 1.5367 |
| factor(ZHONGZU3)4 | 0.5596 | 0.1126 | 4.9711 | 0 | 1.75 | 1.4035 | 2.1821 |
| factor(ZHONGZU3)5 | 0.1013 | 0.1938 | 0.5227 | 0.6012 | 1.1066 | 0.7568 | 1.6181 |
| DRINK10 | 0.109 | 0.0728 | 1.4981 | 0.1341 | 1.1152 | 0.9669 | 1.2861 |
| factor(XIYAN11)2 | -0.1473 | 0.1056 | -1.3941 | 0.1633 | 0.8631 | 0.7017 | 1.0616 |
| factor(XIYAN11)3 | 0.095 | 0.0864 | 1.0995 | 0.2715 | 1.0997 | 0.9283 | 1.3027 |
| GAOXUEYA12 | 0.1868 | 0.0766 | 2.4401 | 0.0147 | 1.2054 | 1.0374 | 1.4005 |
| TANGNIAOBING13 | -0.012 | 0.1012 | -0.1188 | 0.9054 | 0.988 | 0.8102 | 1.2049 |
| FEIBING14 | -0.1023 | 0.086 | -1.189 | 0.2344 | 0.9027 | 0.7626 | 1.0686 |
| XINGZHANGBING15 | -0.3307 | 0.1192 | -2.7754 | 0.0055 | 0.7184 | 0.5688 | 0.9074 |
| GANBING16 | 0.2305 | 0.1946 | 1.184 | 0.2364 | 1.2592 | 0.8598 | 1.8441 |
| DANBAIZHI17 | 0.0047 | 0.0026 | 1.8113 | 0.0701 | 1.0047 | 0.9996 | 1.0099 |
| TANSHUI18 | 0.0064 | 0.0015 | 4.2399 | 0 | 1.0064 | 1.0034 | 1.0094 |
| XIANWEI19 | -0.0207 | 0.0065 | -3.1951 | 0.0014 | 0.9795 | 0.9671 | 0.992 |
| ZHIFANG20 | 0.006 | 0.0037 | 1.6357 | 0.1019 | 1.006 | 0.9988 | 1.0133 |
| SHUIFEN21 | -1e-04 | 0 | -3.3474 | 8e-04 | 0.9999 | 0.9998 | 0.9999 |
| NENGLIANG22 | -0.001 | 4e-04 | -2.712 | 0.0067 | 0.999 | 0.9983 | 0.9997 |
| XINBIE1 | 0.8904 | 0.0803 | 11.0894 | 0 | 2.4362 | 2.0814 | 2.8514 |
| AGE2 | -0.0061 | 0.0026 | -2.387 | 0.017 | 0.9939 | 0.9889 | 0.9989 |
| factor(JIAOYU4)2 | -0.0517 | 0.0881 | -0.5869 | 0.5573 | 0.9496 | 0.799 | 1.1286 |
| factor(JIAOYU4)3 | -0.4001 | 0.0858 | -4.6622 | 0 | 0.6702 | 0.5665 | 0.793 |
| factor(HUNYING5)2 | 0.0542 | 0.0823 | 0.659 | 0.5099 | 1.0557 | 0.8984 | 1.2406 |
| factor(HUNYING5)3 | 0.0255 | 0.0934 | 0.2736 | 0.7844 | 1.0259 | 0.8543 | 1.2319 |
| PIR6 | -0.1372 | 0.0694 | -1.9757 | 0.0482 | 0.8718 | 0.7609 | 0.9989 |
| factor(BMI7)2 | -0.1826 | 0.0799 | -2.2843 | 0.0224 | 0.8331 | 0.7123 | 0.9744 |
| factor(BMI7)3 | -0.423 | 0.0828 | -5.1103 | 0 | 0.6551 | 0.557 | 0.7705 |
| YIYU8 | -0.6261 | 0.0969 | -6.4601 | 0 | 0.5347 | 0.4422 | 0.6465 |

Chi-square tests for linear terms

|  |  |  |  |
| --- | --- | --- | --- |
|  | df | Chi.sq | p-value |
| factor(YUNDONG9) | 1 | 0.7218 | 0.3956 |
| factor(ZHONGZU3) | 4 | 29.4542 | 0 |
| DRINK10 | 1 | 2.2443 | 0.1341 |
| factor(XIYAN11) | 2 | 7.1581 | 0.0279 |
| GAOXUEYA12 | 1 | 5.9539 | 0.0147 |
| TANGNIAOBING13 | 1 | 0.0141 | 0.9054 |
| FEIBING14 | 1 | 1.4137 | 0.2344 |
| XINGZHANGBING15 | 1 | 7.7029 | 0.0055 |
| GANBING16 | 1 | 1.4019 | 0.2364 |
| DANBAIZHI17 | 1 | 3.2809 | 0.0701 |
| TANSHUI18 | 1 | 17.977 | 0 |
| XIANWEI19 | 1 | 10.2089 | 0.0014 |
| ZHIFANG20 | 1 | 2.6754 | 0.1019 |
| SHUIFEN21 | 1 | 11.2053 | 8e-04 |
| NENGLIANG22 | 1 | 7.3551 | 0.0067 |
| XINBIE1 | 1 | 122.9755 | 0 |
| AGE2 | 1 | 5.698 | 0.017 |
| factor(JIAOYU4) | 2 | 27.9791 | 0 |
| factor(HUNYING5) | 2 | 0.4616 | 0.7939 |
| PIR6 | 1 | 3.9036 | 0.0482 |
| factor(BMI7) | 2 | 26.2488 | 0 |
| YIYU8 | 1 | 41.7331 | 0 |

Approximate significance of smooth terms

|  |  |  |  |  |
| --- | --- | --- | --- | --- |
|  | edf | Ref.df | Chi.sq | p-value |
| s(CDAI23):factor(YUNDONG9)1 | 1.7321 | 2.2133 | 8.4418 | 0.0171 |
| s(CDAI23):factor(YUNDONG9)2 | 1.0028 | 1.0057 | 5.9259 | 0.0152 |

Model statistics

|  |  |
| --- | --- |
| N: | 10904 |
| Adj. r-square: | 0.0544 |
| Deviance explained: | 0.0792 |
| UBRE score (sp.criterion): | -0.3613 |
| Scale estimate: | 1 |
| family: | binomial |
| link function: | logit |
