# Supplementary material for: Association between the composite dietary antioxidant index and constipation: Evidence from NHANES 2005–2010
Source: PLoS One. 2024 Sep 27;19(9):e0311168. doi: 10.1371/journal.pone.0311168 (PMC11432863; doi:10.1371/journal.pone.0311168)
Supplement: S1 File — (ZIP) [file pone.0311168.s001.zip › CDAI/all/PROJ3_1_tbl/PROJ3_1_tbl.htm]

## ½»»¥×÷ÓÃ¼ìÑé

|  |  |  |  |
| --- | --- | --- | --- |
| Model | ALCOHOL.INTAKE: 1 | ALCOHOL.INTAKE: 2 | P interaction |
| Crude | 0.881 (0.859, 0.903) <0.0001 | 0.954 (0.924, 0.985) 0.0039 | 0.0001 |
| Model II | 0.936 (0.904, 0.969) 0.0002 | 0.997 (0.959, 1.036) 0.8722 | 0.0022 |
| Model II\* | 0.935 (0.899, 0.973) 0.0008 | 0.998 (0.951, 1.048) 0.9432 | 0.0428 |

Results in table:
¦Â (95%CI) Pvalue / OR (95%CI) Pvalue
½á¹û±äÁ¿: CONSTIPATION
Î£ÏÕÒòËØ: CDAI
Ð§Ó¦ÐÞÊÎÒò×Ó: ALCOHOL.INTAKE
Model II µ÷ÕûÁË: XINBIE1, AGE2, ZHONGZU3, JIAOYU4, HUNYING5, PIR6, BMI7, YIYU8, YUNDONG9, XIYAN11, GAOXUEYA12, TANGNIAOBING13, FEIBING14, XINGZHANGBING15, GANBING16, DANBAIZHI17, TANSHUI18, XIANWEI19, ZHIFANG20, SHUIFEN21, NENGLIANG22
Model II\* µ÷ÕûÁË: XINBIE1, AGE2, ZHONGZU3, JIAOYU4, HUNYING5, PIR6, BMI7, YIYU8, YUNDONG9, XIYAN11, GAOXUEYA12, TANGNIAOBING13, FEIBING14, XINGZHANGBING15, GANBING16, DANBAIZHI17, TANSHUI18, XIANWEI19, ZHIFANG20, SHUIFEN21, NENGLIANG22 and the interaction terms for following variables: XINBIE1, ZHONGZU3, JIAOYU4, PIR6, YIYU8, XIYAN11, TANGNIAOBING13, XINGZHANGBING15, DANBAIZHI17, TANSHUI18, XIANWEI19, SHUIFEN21, NENGLIANG22
´Ë±íÓÃÒ×õÍ³¼ÆÈí¼þ (www.empowerstats.com) ºÍRÈí¼þÉú³É£¬Éú³ÉÈÕÆÚ£º 2024-06-26
¸÷Ä£ÐÍËùÓÃµÄÑù±¾Á¿

|  |  |  |  |  |  |
| --- | --- | --- | --- | --- | --- |
| Y | Strata | X | Model | 1 | 2 |
| CONSTIPATION | Total | CDAI | Crude | 7838 | 3066 |
| CONSTIPATION | Total | CDAI | Model II | 7838 | 3066 |
| CONSTIPATION | Total | CDAI | Model II\* | 7838 | 3066 |
