# Supplementary material for: Association between the composite dietary antioxidant index and constipation: Evidence from NHANES 2005–2010
Source: PLoS One. 2024 Sep 27;19(9):e0311168. doi: 10.1371/journal.pone.0311168 (PMC11432863; doi:10.1371/journal.pone.0311168)
Supplement: S1 File — (ZIP) [file pone.0311168.s001.zip › CDAI/all/PROJ3_3_tbl/PROJ3_3_tbl.htm]

## ½»»¥×÷ÓÃ¼ìÑé

|  |  |  |  |  |  |  |
| --- | --- | --- | --- | --- | --- | --- |
| Model | ZHONGZU3: 1 | ZHONGZU3: 2 | ZHONGZU3: 3 | ZHONGZU3: 4 | ZHONGZU3: 5 | P interaction |
| Crude | 0.917 (0.872, 0.963) 0.0006 | 0.950 (0.895, 1.008) 0.0891 | 0.866 (0.840, 0.892) <0.0001 | 0.947 (0.913, 0.982) 0.0031 | 0.865 (0.773, 0.969) 0.0120 | 0.0015 (0.9701 #) |
| Model II | 0.975 (0.922, 1.031) 0.3781 | 0.995 (0.933, 1.062) 0.8904 | 0.932 (0.898, 0.967) 0.0002 | 0.987 (0.947, 1.029) 0.5449 | 0.930 (0.829, 1.043) 0.2158 | 0.0767 (0.7798 #) |
| Model II\* | 0.935 (0.857, 1.019) 0.1266 | 0.983 (0.891, 1.084) 0.7336 | 0.952 (0.910, 0.996) 0.0336 | 0.991 (0.936, 1.049) 0.7580 | 0.847 (0.675, 1.062) 0.1496 | 0.5372 (0.5845 #) |

Results in table:
¦Â (95%CI) Pvalue / OR (95%CI) Pvalue
# interaction trend test (treat
ZHONGZU3
as continuous)
½á¹û±äÁ¿: BIANMI24
Î£ÏÕÒòËØ: CDAI23
Ð§Ó¦ÐÞÊÎÒò×Ó: ZHONGZU3
Model II µ÷ÕûÁË: JIAOYU4, HUNYING5, PIR6, BMI7, YIYU8, YUNDONG9, DRINK10, XIYAN11, GAOXUEYA12, TANGNIAOBING13, FEIBING14, XINGZHANGBING15, GANBING16, DANBAIZHI17, TANSHUI18, XIANWEI19, ZHIFANG20, SHUIFEN21, NENGLIANG22, XINBIE1, AGE2
Model II\* µ÷ÕûÁË: JIAOYU4, HUNYING5, PIR6, BMI7, YIYU8, YUNDONG9, DRINK10, XIYAN11, GAOXUEYA12, TANGNIAOBING13, FEIBING14, XINGZHANGBING15, GANBING16, DANBAIZHI17, TANSHUI18, XIANWEI19, ZHIFANG20, SHUIFEN21, NENGLIANG22, XINBIE1, AGE2 and the interaction terms for following variables: JIAOYU4, HUNYING5, PIR6, BMI7, YIYU8, YUNDONG9, DRINK10, GAOXUEYA12, TANGNIAOBING13, FEIBING14, XINGZHANGBING15, DANBAIZHI17, TANSHUI18, XIANWEI19, ZHIFANG20, SHUIFEN21, NENGLIANG22, XINBIE1, AGE2
´Ë±íÓÃÒ×õÍ³¼ÆÈí¼þ (www.empowerstats.com) ºÍRÈí¼þÉú³É£¬Éú³ÉÈÕÆÚ£º 2024-06-24
¸÷Ä£ÐÍËùÓÃµÄÑù±¾Á¿

|  |  |  |  |  |  |  |  |  |
| --- | --- | --- | --- | --- | --- | --- | --- | --- |
| Y | Strata | X | Model | 1 | 2 | 3 | 4 | 5 |
| BIANMI24 | Total | CDAI23 | Crude | 1851 | 902 | 5643 | 2107 | 401 |
| BIANMI24 | Total | CDAI23 | Model II | 1851 | 902 | 5643 | 2107 | 401 |
| BIANMI24 | Total | CDAI23 | Model II\* | 1851 | 902 | 5643 | 2107 | 401 |
