# Supplementary material for: Association between the composite dietary antioxidant index and constipation: Evidence from NHANES 2005–2010
Source: PLoS One. 2024 Sep 27;19(9):e0311168. doi: 10.1371/journal.pone.0311168 (PMC11432863; doi:10.1371/journal.pone.0311168)
Supplement: S1 File — (ZIP) [file pone.0311168.s001.zip › CDAI/all/PROJ3_5_tbl/PROJ3_5_tbl.htm]

## ½»»¥×÷ÓÃ¼ìÑé

|  |  |  |  |  |
| --- | --- | --- | --- | --- |
| Model | XIYAN11: 1 | XIYAN11: 2 | XIYAN11: 3 | P interaction |
| Crude | 0.877 (0.838, 0.917) <0.0001 | 0.863 (0.824, 0.903) <0.0001 | 0.918 (0.895, 0.941) <0.0001 | 0.0284 (0.0261 #) |
| Model II | 0.941 (0.895, 0.989) 0.0166 | 0.924 (0.877, 0.973) 0.0027 | 0.973 (0.941, 1.006) 0.1121 | 0.0881 (0.0827 #) |
| Model II\* | 0.969 (0.911, 1.031) 0.3166 | 0.884 (0.822, 0.952) 0.0010 | 0.975 (0.940, 1.012) 0.1869 | 0.0430 (0.4474 #) |

Results in table:
¦Â (95%CI) Pvalue / OR (95%CI) Pvalue
# interaction trend test (treat
XIYAN11
as continuous)
½á¹û±äÁ¿: BIANMI24
Î£ÏÕÒòËØ: CDAI23
Ð§Ó¦ÐÞÊÎÒò×Ó: XIYAN11
Model II µ÷ÕûÁË: JIAOYU4, HUNYING5, PIR6, BMI7, YIYU8, YUNDONG9, GAOXUEYA12, TANGNIAOBING13, FEIBING14, XINGZHANGBING15, GANBING16, DANBAIZHI17, TANSHUI18, XIANWEI19, ZHIFANG20, SHUIFEN21, NENGLIANG22, XINBIE1, AGE2, ZHONGZU3, DRINK10
Model II\* µ÷ÕûÁË: JIAOYU4, HUNYING5, PIR6, BMI7, YIYU8, YUNDONG9, GAOXUEYA12, TANGNIAOBING13, FEIBING14, XINGZHANGBING15, GANBING16, DANBAIZHI17, TANSHUI18, XIANWEI19, ZHIFANG20, SHUIFEN21, NENGLIANG22, XINBIE1, AGE2, ZHONGZU3, DRINK10 and the interaction terms for following variables: TANSHUI18, XIANWEI19, ZHIFANG20, SHUIFEN21, NENGLIANG22
´Ë±íÓÃÒ×õÍ³¼ÆÈí¼þ (www.empowerstats.com) ºÍRÈí¼þÉú³É£¬Éú³ÉÈÕÆÚ£º 2024-06-24
¸÷Ä£ÐÍËùÓÃµÄÑù±¾Á¿

|  |  |  |  |  |  |  |
| --- | --- | --- | --- | --- | --- | --- |
| Y | Strata | X | Model | 1 | 2 | 3 |
| BIANMI24 | Total | CDAI23 | Crude | 2848 | 2259 | 5797 |
| BIANMI24 | Total | CDAI23 | Model II | 2848 | 2259 | 5797 |
| BIANMI24 | Total | CDAI23 | Model II\* | 2848 | 2259 | 5797 |
