# Supplementary material for: Association between the composite dietary antioxidant index and constipation: Evidence from NHANES 2005–2010
Source: PLoS One. 2024 Sep 27;19(9):e0311168. doi: 10.1371/journal.pone.0311168 (PMC11432863; doi:10.1371/journal.pone.0311168)
Supplement: S1 File — (ZIP) [file pone.0311168.s001.zip › CDAI/all/PROJ3_6_tbl/PROJ3_6_tbl.htm]

## ½»»¥×÷ÓÃ¼ìÑé

|  |  |  |  |
| --- | --- | --- | --- |
| Model | GAOXUEYA12: 1 | GAOXUEYA12: 2 | P interaction |
| Crude | 0.882 (0.856, 0.910) <0.0001 | 0.908 (0.885, 0.932) <0.0001 | 0.1514 |
| Model II | 0.948 (0.913, 0.984) 0.0054 | 0.965 (0.933, 0.999) 0.0458 | 0.3540 |
| Model II\* | 0.977 (0.936, 1.020) 0.2937 | 0.942 (0.905, 0.982) 0.0044 | 0.2292 |

Results in table:
¦Â (95%CI) Pvalue / OR (95%CI) Pvalue
½á¹û±äÁ¿: BIANMI24
Î£ÏÕÒòËØ: CDAI23
Ð§Ó¦ÐÞÊÎÒò×Ó: GAOXUEYA12
Model II µ÷ÕûÁË: JIAOYU4, HUNYING5, PIR6, BMI7, YIYU8, YUNDONG9, TANGNIAOBING13, FEIBING14, XINGZHANGBING15, GANBING16, DANBAIZHI17, TANSHUI18, XIANWEI19, ZHIFANG20, SHUIFEN21, NENGLIANG22, XINBIE1, AGE2, ZHONGZU3, DRINK10, XIYAN11
Model II\* µ÷ÕûÁË: JIAOYU4, HUNYING5, PIR6, BMI7, YIYU8, YUNDONG9, TANGNIAOBING13, FEIBING14, XINGZHANGBING15, GANBING16, DANBAIZHI17, TANSHUI18, XIANWEI19, ZHIFANG20, SHUIFEN21, NENGLIANG22, XINBIE1, AGE2, ZHONGZU3, DRINK10, XIYAN11 and the interaction terms for following variables: DANBAIZHI17, XIANWEI19, NENGLIANG22
´Ë±íÓÃÒ×õÍ³¼ÆÈí¼þ (www.empowerstats.com) ºÍRÈí¼þÉú³É£¬Éú³ÉÈÕÆÚ£º 2024-06-24
¸÷Ä£ÐÍËùÓÃµÄÑù±¾Á¿

|  |  |  |  |  |  |
| --- | --- | --- | --- | --- | --- |
| Y | Strata | X | Model | 1 | 2 |
| BIANMI24 | Total | CDAI23 | Crude | 5493 | 5411 |
| BIANMI24 | Total | CDAI23 | Model II | 5493 | 5411 |
| BIANMI24 | Total | CDAI23 | Model II\* | 5493 | 5411 |
