# Supplementary material for: Association between the composite dietary antioxidant index and constipation: Evidence from NHANES 2005–2010
Source: PLoS One. 2024 Sep 27;19(9):e0311168. doi: 10.1371/journal.pone.0311168 (PMC11432863; doi:10.1371/journal.pone.0311168)
Supplement: S1 File — (ZIP) [file pone.0311168.s001.zip › CDAI/all/PROJ3_7_tbl/PROJ3_7_tbl.htm]

## ½»»¥×÷ÓÃ¼ìÑé

|  |  |  |  |
| --- | --- | --- | --- |
| Model | TANGNIAOBING13: 1 | TANGNIAOBING13: 2 | P interaction |
| Crude | 0.871 (0.822, 0.924) <0.0001 | 0.902 (0.883, 0.920) <0.0001 | 0.2774 |
| Model II | 0.937 (0.879, 0.997) 0.0414 | 0.961 (0.931, 0.991) 0.0110 | 0.4178 |
| Model II\* | 0.916 (0.838, 1.002) 0.0552 | 0.964 (0.933, 0.995) 0.0230 | 0.2873 |

Results in table:
¦Â (95%CI) Pvalue / OR (95%CI) Pvalue
½á¹û±äÁ¿: BIANMI24
Î£ÏÕÒòËØ: CDAI23
Ð§Ó¦ÐÞÊÎÒò×Ó: TANGNIAOBING13
Model II µ÷ÕûÁË: JIAOYU4, HUNYING5, PIR6, BMI7, YIYU8, YUNDONG9, FEIBING14, XINGZHANGBING15, GANBING16, DANBAIZHI17, TANSHUI18, XIANWEI19, ZHIFANG20, SHUIFEN21, NENGLIANG22, XINBIE1, AGE2, ZHONGZU3, DRINK10, XIYAN11, GAOXUEYA12
Model II\* µ÷ÕûÁË: JIAOYU4, HUNYING5, PIR6, BMI7, YIYU8, YUNDONG9, FEIBING14, XINGZHANGBING15, GANBING16, DANBAIZHI17, TANSHUI18, XIANWEI19, ZHIFANG20, SHUIFEN21, NENGLIANG22, XINBIE1, AGE2, ZHONGZU3, DRINK10, XIYAN11, GAOXUEYA12 and the interaction terms for following variables: JIAOYU4, DANBAIZHI17, XIANWEI19, SHUIFEN21
´Ë±íÓÃÒ×õÍ³¼ÆÈí¼þ (www.empowerstats.com) ºÍRÈí¼þÉú³É£¬Éú³ÉÈÕÆÚ£º 2024-06-24
¸÷Ä£ÐÍËùÓÃµÄÑù±¾Á¿

|  |  |  |  |  |  |
| --- | --- | --- | --- | --- | --- |
| Y | Strata | X | Model | 1 | 2 |
| BIANMI24 | Total | CDAI23 | Crude | 1601 | 9303 |
| BIANMI24 | Total | CDAI23 | Model II | 1601 | 9303 |
| BIANMI24 | Total | CDAI23 | Model II\* | 1601 | 9303 |
