# Supplementary material for: Association between the composite dietary antioxidant index and constipation: Evidence from NHANES 2005–2010
Source: PLoS One. 2024 Sep 27;19(9):e0311168. doi: 10.1371/journal.pone.0311168 (PMC11432863; doi:10.1371/journal.pone.0311168)
Supplement: S1 File — (ZIP) [file pone.0311168.s001.zip › CDAI/all/PROJ3_9_tbl/PROJ3_9_tbl.htm]

## ¶à¸ö»Ø¹é·½³Ì

|  |  |  |
| --- | --- | --- |
| Exposure | Non-adjusted | Adjust |
| HULUOBUSU30 ËÄ·Ö×é |  |  |
| Q1 | 1.0 | 1.0 |
| Q2 | 0.704 (0.598, 0.830) 0.00003 | 0.844 (0.711, 1.002) 0.05289 |
| Q3 | 0.755 (0.642, 0.887) 0.00062 | 1.041 (0.873, 1.240) 0.65718 |
| Q4 | 0.521 (0.437, 0.621) <0.00001 | 0.802 (0.656, 0.979) 0.03046 |

±íÖÐÊý¾Ý£º
¦Â (95%CI) Pvalue / OR (95%CI) Pvalue
½á¹û±äÁ¿: BIANMI24
±©Â¶±äÁ¿: HULUOBUSU30 ËÄ·Ö×é
 Non-adjusted model adjust for: None
 Adjust model adjust for: XINBIE1; AGE2; ZHONGZU3; JIAOYU4; HUNYING5; PIR6; BMI7; YIYU8; YUNDONG9; DRINK10; XIYAN11; GAOXUEYA12; TANGNIAOBING13; FEIBING14; XINGZHANGBING15; GANBING16; DANBAIZHI17; TANSHUI18; XIANWEI19; ZHIFANG20; SHUIFEN21; NENGLIANG22
´Ë±íÓÃÒ×õÍ³¼ÆÈí¼þ (www.empowerstats.com) ºÍRÈí¼þÉú³É£¬Éú³ÉÈÕÆÚ£º 2024-06-24
¸÷Ä£ÐÍËùÓÃµÄÑù±¾Á¿

|  |  |  |  |
| --- | --- | --- | --- |
| Outcome | Exposure | Non-adjusted | Adjust |
| BIANMI24 | HULUOBUSU30 ËÄ·Ö×é | 10904 | 10904 |
