# Supplementary material for: Association between the composite dietary antioxidant index and constipation: Evidence from NHANES 2005–2010
Source: PLoS One. 2024 Sep 27;19(9):e0311168. doi: 10.1371/journal.pone.0311168 (PMC11432863; doi:10.1371/journal.pone.0311168)
Supplement: S1 File — (ZIP) [file pone.0311168.s001.zip › CDAI/PLOTS ONE/Cover letter.docx]

Dear Editor-in-Chief,

I am pleased to submit our manuscript entitled "The Relationship Between Composite Dietary Antioxidant Index and Constipation Among Adults in the United States" for consideration for publication in PLOS ONE. We believe our findings provide significant insights into the role of dietary antioxidants in managing chronic constipation and contribute to the broader understanding of dietary factors in gastrointestinal health.

Our study investigates the potential link between the Composite Dietary Antioxidant Index (CDAI) and chronic constipation using data from the National Health and Nutrition Examination Survey (NHANES) collected between 2005 and 2010. By employing multiple statistical methods, including multivariable logistic regression, subgroup analysis, and smooth curve fitting analysis, we found that higher CDAI is significantly associated with a lower risk of chronic constipation. Notably, our results indicate that individuals in the highest quartile of CDAI have a significantly reduced prevalence of constipation compared to those in the lowest quartile. Additionally, our analysis revealed that gender, alcohol intake, and smoking status may influence this association.

The findings underscore the importance of dietary antioxidants in maintaining gut health and suggest that increasing dietary antioxidant intake could be a viable strategy for reducing constipation prevalence. We believe our study provides comprehensive guidance for clinical and public health practices.

We confirm that this manuscript has not been published elsewhere and is not under consideration by another journal. All authors have approved the manuscript and agree with its submission to PLOS ONE. We have no conflicts of interest to disclose.

We hope that the novel insights presented in our study will be of interest to the readership of PLOS ONE. Thank you for considering our submission. We look forward to your positive response.

Sincerely,

Shouxin Wei

Department of Gastrointestinal Surgery,Suining Central Hospital,Suining,China

Email:1079656665@qq.com
