# Supplementary material for: Association between the composite dietary antioxidant index and constipation: Evidence from NHANES 2005–2010
Source: PLoS One. 2024 Sep 27;19(9):e0311168. doi: 10.1371/journal.pone.0311168 (PMC11432863; doi:10.1371/journal.pone.0311168)
Supplement: S1 File — (ZIP) [file pone.0311168.s001.zip › CDAI/PLOTS ONE/PLOSOne_formatting_sample_main_body zh.pdf]

订阅DeepL Pro以翻译大型文件。
欲了解更多信息，请访问www.DeepL.com/pro。

PLOS ONE

稿件正文格式指南

摘要

Lorem ipsum dolor sit amet, consectetur adipiscing elit. 雌激素分泌旺盛，胎儿发育不良 interdum。牆壁上沒有絲毫的痕跡。前庭 "......"(《梵蒂冈城》)躯干无光泽。纤细 "......""我的心在颤抖。Nam lacus felis, fermentum sit amet nulla ac， tristique ultrices tellus.整幅画的结构清晰可见，eu fermentum magna pellentesque vitae.整体半透明 vel pulvinar.悬挂的鳞片上有一圈黑色的鳞片。前胸 在这一点上，我们可以看到，"寓意 "与 "寓意 "是相辅相成的。前庭 在这之前，"箭在弦上"，"蓄势待发"。无 dictum consectetur leo.Ut vulputate ipsum purus, a interdum nibh 1……"(《梵文诗歌》) Nulla interdum accumsan lectus, sed auctor elit accumsan a. Suspendisse quis rhoncus nibh.The verum est de illic.

导言

Lorem ipsum dolor sit amet, consectetur adipiscing elit. 雌激素分泌旺盛，胎儿发育不良 interdum。牆壁上沒有絲毫的痕跡。前庭 躯干臃肿，身体虚弱。

材料和方法

Lorem ipsum dolor sit amet, consectetur adipiscing elit. Vestibulum adipiscing urna ut lectus gravida, vitae (Fig 1) interdum。牆壁上沒有絲毫的痕跡。前庭 "......"(《梵蒂冈城》)躯干无光泽。纤细 图 1 和图 2。Nam sit amet nulla lacus a, 图 1 和图 2 ultrices tellus.Integer rutrum aliquet sapien, eu fermentum magna pellentesque vitae.

图 1。这是图 1 标题。这是图 1 图例。

图 2。这是图 2 标题。这是图 2 图例。

数字文件命名

- 图表文件应保存为 "Fig1.tif"、"Fig2.eps "等。
- 可接受的数字文件格式有".tif"、".tiff "和 ".eps"。
- 图表应作为单独文件单独上传。
- PLOS ONE 图表指南可在此处查阅：http://journals.plos.org/plosone/s/figures

第 1 级标题

- 所有主要部分（摘要、引言、材料与方法、结果、讨论等）均使用 1 级标题。
- 粗体，18pt 字体。
- 仅在需要时使用斜体和文本格式（如属名和种名、基因等）。
- 标题应使用句子大小写（仅大写标题的第一个单词、副标题的第一个单词以及任何专有名词和属名）。

注：请勿在摘要中引用图、表、辅助信息或参考文献。

图表引用

- 在引用图表时，请注明 "图 1"、"图 2 "等。
- 按顺序引用图和表。
- 不要在 "图 1 "之前引用 "图 2"。
- 多图引用时应注明 "图 1 和图 2"、"图 1-3 "等。

图表说明

- 每幅图的标题应直接出现在首次引用的段落之后。
- 标题中不要包含表格。
- 图表标题使用粗体字。

Lorem ipsum dolor sit amet, consectetur adipiscing elit. 雌激素分泌旺盛，胎儿发育不良 interdum。Donec p^2 et q^2 tincidunt porta sem nec hendrerit.

$$p^2 + 2pq + q^2 = 1 \qquad (1)$$

Vestibulum nec pharetra quam, vitae convallis nunc.躯干 in mattis sapien.纤尘不染。Nam lacus felis、发酵的物质无处藏身，三层结构纤毫毕现。整体 aliquet sapien, eu fermentum magna pellentesque vitae.整体 $(p+q)^2 = 1$。

基因分型

Lorem ipsum dolor sit amet, consectetur adipiscing elit. 雌激素分泌旺盛，胎儿发育不良 interdum。岂能苟且偷生？Omnes tuum basi sunt pertinent ad nos.无光泽。纤尘不染 "(《梵蒂冈城宣言》)Nam lacus felis, fermentum sit amet nulla ac 、 三层楼高的空间。茎干粗壮，有鞘，茎干发酵 magna pellentesque vitae.Integer semper viverra mauris vel pulvinar et alst.

全基因组 RFLP 分析

Lorem ipsum dolor sit amet, consectetur adipiscing elit. 雌激素分泌旺盛，胎儿发育不良 interdum。墙壁上沒有絲毫的痕跡。前庭 "......"(《梵蒂冈城》)躯干无光泽。纤细

显示/编号公式

- 在 Mathtype 或方程工具中格式化显示方程。
- 请勿使用图形对象。

sodales vulputate auctor.Numquam iens dare tibi up.

注：为便于使用，本文件采用单倍行距段落格式。请以双倍行距段落格式提交稿件。

内联方程

- 在 Mathtype 或 Equation Tools 中以常规文本或内联方程的形式进行格式化。
- 请勿使用符号字体。
- 请勿使用图形对象。

二级标题

- 主要章节的小节使用二级标题。
- 粗体，16pt 字体。
- 仅在必要时使用斜体和文本格式。
- 使用句子的情况。

三级标题

- 二级标题内的小节使用三级标题。
- 粗体，14pt 字体。
- 仅在必要时使用斜体和文本格式。
- 使用句子的情况。

结果和讨论

Lorem ipsum dolor sit amet, consectetur adipiscing elit. Vestibulum adipiscing urna ut lectus gravida, et bland 表 1 我们的 "巢穴 "在这里，我们的 "巢穴 "在这里，我们的 "巢穴 "在这 quam, vitae convalli.Fido nemo.

表 1。这就是表 1 的标题。

	化学 W	化学 X	化学 Y	化学 Z
**化学 1**	反应 1W	反应 1X	反应 1Y	反应 1Z
**化学 2**	反应 2W	反应 2X	反应 2Y	反应 2Z
**化学 3**	反应 3W[a]	反应 3X	反应 3Y[b]	反应 3Z
**化学 4**	反应 4W	反应 4X	反应 4Y	反应 4Z
**化学 5**	反应 5W	反应 5X	反应 5Y	反应 5Z

这就是表 1 的图例。
[a]此处为表格脚注。
[b]脚注应在表格中使用相应的符号。

表格和表格引用

- 表格应注明为 "表 1"、" 表 2 "等。
- 将多个表格引用为 "表 1 和表 2"、"表 1-3 "等。
- 表格应直接放在首次引用表格的段落之后。
- 表格必须以 Microsoft Word 单元格为基础，或嵌入 Microsoft Excel。
- 不要使用空行来创建间距。
- 请勿包含图形对象、图像或彩色文字。
- 有关更完整的说明，请参见《PLOS ONE 表格指南》：http://journals.plos.org/plosone/s/tables

结论

Loremipsum dolor sit amet, consectetur adipiscing [1-5]。雌激素分泌旺盛，胎儿发育不良 interdum。牆壁上沒有絲毫的痕跡。前庭 "......"(《梵蒂冈城》)躯干无光泽。纤细 图 S1 和 S2 表。

参考文献

- 在括号中引用参考文献（如 "[1]"或"[2-5]" 或"[3,7,9]"）。
- 参考文献必须按首次提及的顺序引用。

佐证资料引用

- 以 "S1 图"、"S1 表 "等格式引用佐证资料。
- 将多个文件引用为 "S1 和 S2 图"、"S1-S3 图 "等。

3

- 不要求引用每个佐证资料文件。
- 证明资料应作为单独文件单独上传。

致谢

Lorem ipsum dolor sit amet, consectetur adipiscing elit. 雌激素分泌旺盛，胎儿发育不良 interdum。

致谢

- 请勿在 "致谢 "中包含资助或竞争利益信息。

参考文献

1. Doe J、Data A、van Stats J、Testperson M、Ribosome D Jr、McBio GHT, et al. 这是文章标题。PLoS ONE. 2017;12(12):e00000. Doi: 10.1371/journal.pone.0000000
2. Doe J、Data A、van Stats J、Testperson M、Ribosome D Jr、McBio GHT 等人.卡通景观中的兔子动态。PLoS ONE.2017 年即将出版。

参考资料

- 参考文献应列在正文之后，辅助信息之前。
- 超过六位作者的参考文献应列出前六位作者姓名，后加 "等"。
- 请点击此处查看 PLOS ONE 参考文献指南: http://journals.plos.org/plosone/s/submission-guidelines#loc-references

辅助信息

这是 S1 图标题。这是 S1 图的图例。

这是 S2 图标题。这是 S2 图例。

S1 表。这是 S1 表标题。这是 S1 表图例。

S2 表格。这是 S2 表标题。这是 S2 表图例。

S1 文件。这是 S1 文件标题。这是 S1 文件图例。

佐证资料标题

- 在稿件末尾的 "辅助信息 "部分列出辅助信息标题。
- 使用 1 级标题。
- 标题使用粗体字。
- 辅助信息文件不需要完整的标题；只需要完整的标签（"S1 图"）。

佐证资料的文件命名

- 佐证资料文件应保存为 "S1_Fig.tif"、"S1_File.pdf "等。
- 支持所有文件类型。
