# Supplementary material for: Association between the composite dietary antioxidant index and constipation: Evidence from NHANES 2005–2010
Source: PLoS One. 2024 Sep 27;19(9):e0311168. doi: 10.1371/journal.pone.0311168 (PMC11432863; doi:10.1371/journal.pone.0311168)
Supplement: Attachment — Submitted filename: Response to Reviewers.docx [file pone.0311168.s002.docx]

**Response to Reviewers**

Dear Editor and Reviewers,

We would like to thank you for the time and effort you have put into reviewing our manuscript, titled " Association between the composite dietary antioxidant index and constipation: evidence from NHANES 2005-2010." We appreciate the valuable feedback and suggestions provided, which have greatly helped us improve our work. We have carefully considered all comments and have made the necessary revisions to address each of them. We highlighted all the revisions in red colour.Below, we provide a detailed point-by-point response to each comment.

### ****Reviewer #1:****

1. **Comment:** Define abbreviations in their first use and make sure that abbreviated forms are being used after the definition.

**Response:** Thank you for this suggestion. We have amended the article to define abbreviations the first time they are used and to ensure that the abbreviated form is used after the definition.

**Changes in Manuscript:** **For example, we removed more than definitions in the introductory section, making sure to use the abbreviated form after the first definition**

2. **Comment:** Mention the clinical utility of your findings for a primary care physician to the discussion section.

**Response:** We agree with the reviewer's suggestion and have increased clinical relevance for PCPs in the discussion sections. This change enhances the manuscript's applicability to primary care settings by providing more targeted insights and practical recommendations that are directly relevant to primary care physicians.

**Changes in Manuscript: We added clinical utility for the study's primary care physicians in the discussion section.**

3. **Comment:** In the conclusions, suggest future studies on this topic to overcome your limitations.

**Response:** Thank you for bringing this to our attention. We have added future research directions to address our research shortcomings.

**Changes in Manuscript:** In the concluding section, we emphasise the importance of future research in different regions and populations.

### ****Reviewer #2:****

1. **Comment:** A paragraph summarizing the clinical take-home message of this manuscript should be added to the discussion.

**Response:** Thank you for this suggestion. In our article, we added a paragraph to discuss the clinical points of the study in terms of clinical prevention and treatment of constipation.

**Changes in Manuscript:** **In the discussion section, we emphasised the importance of antioxidant diet, exercise and other factors in the prevention of constipation, and stressed that holistic health management is an important strategy for the prevention and relief of constipation.**

2. **Comment:** The references prior to 2010 could be updated with those after 2010 since they provide more up-to-date findings.

**Response:** Thank you for highlighting this. We have made changes to the citations.The updated manuscript now reflects this improvement.

**Changes in Manuscript: References prior to 2010 have been updated with more recent studies.**

3. **Comment:** The first paragraph of the discussion should mention the main findings of the manuscript.

**Response:** We appreciate this suggestion and have revised the first paragraph of the discussion to ensure that the first paragraph of the discussion section refers to the main findings of the paper.

**Changes in Manuscript:** Revise the main findings of the study to the first paragraph so that the reader can see the results at a glance.

We believe these revisions have significantly strengthened the manuscript, and we hope that it now meets your expectations for publication. We are grateful for the constructive feedback and remain at your disposal for any further questions or clarifications.

Thank you once again for your consideration of our work.

Sincerely,

Shouxin Wei

Department of Gastrointestinal Surgery,Suining Central Hospital,Suining,China
